# Supplementary material for: Highly efficient recycling of polyester wastes to diols using Ru and Mo dual-atom catalyst
Source: Nat Commun. 2024 Jul 4;15:5630. doi: 10.1038/s41467-024-49880-z (PMC11224329; doi:10.1038/s41467-024-49880-z)
Supplement: Supplementary file 1 — Supplementary Information [file 41467_2024_49880_MOESM1_ESM.pdf]

Supplementary Information

Highly Efficient Recycling of Polyester Wastes to Diols Using Ru and Mo Dual-Atom Catalyst

Minhao Tang[1,2,6], Ji Shen[3,6], Yiding Wang[1,2], Yanfei Zhao[1,2*], Tao Gan[4], Xusheng Zheng[5], Dingsheng Wang[3*], Buxing Han[1,2], Zhimin Liu[1,2*]

Affiliations:

[1]Beijing National Laboratory for Molecular Sciences, CAS Laboratory of Colloid and Interface and Thermodynamics, CAS Research/Education Center for Excellence in Molecular Sciences, Center for Carbon Neutral Chemistry, Institute of Chemistry, Chinese Academy of Sciences, Beijing, 100190, China.

[2]University of Chinese Academy of Sciences, Beijing, 100049, China.

[3]Department of Chemistry, Tsinghua University, Beijing, 100084, China.

[4]Shanghai Synchrotron Radiation Facility, Shanghai Advanced Research Institute, Chinese Academy of Sciences, Shanghai, 201204, China.

[5]National Synchrotron Radiation Laboratory, University of Science and Technology of China, Hefei, Anhui, 230029, China.

[6] These authors contributed equally: Minhao Tang, Ji Shen.

*Corresponding authors. Email: lianyi302@iccas.ac.cn, wangdingsheng@mail.tsinghua.edu.cn, liuzm@iccas.ac.cn;

Characterizations

XRD analysis was performed on a Rigaku D/Max-2500 (Rigaku Co., Japan) diffractometer equipped with Cu Kα1 radiation (λ = 1.54056 Å). X-ray photoelectron spectroscopy (XPS) study was carried out on an X-ray photoelectron spectrometer (ESCALab 250Xi, Thermo Fisher Scientific, USA) using 200 W Al-Kα radiation. The base pressure was about 3 × 10^{-10} mbar, with 284.8 eV hydrocarbon C *1s* line from exogenous carbon as an energy reference. The contents of metal elements in the catalysts were determined by inductively coupled plasma optical emission spectrometer (ICP-OES, Agilent 5110, Agilent Technologies Inc., USA). The metal contents were determined by ICP analysis on FlashSmart elemental analyzer. Fourier Transform Infrared (FTIR) spectra were collected on Bruker Invenio-s. In situ Raman spectra were recorded using a Raman spectrometer equipped with a 532 nm laser, with a spectral Raman shift range of 200 to 4000 cm^{-1}, a resolution of 1.3 cm^{-1}, a 1200/mm grating, and a collection time of 60 seconds. The high-resolution transmission electron microscopy (HR-TEM) was used to observe the morphology of samples. HR-TEM images were collected using a JEOL JEM-2100F TEM. The Aberration-corrected high-angle circular dark-field scanning transmission electron microscopy (AC HAADF-STEM) characterization was performed on a Spectra 300 S/TEM transmission electron microscope with spherical aberration corrector. Electron paramagnetic resonance values were collected using a JEOL JES-FA 200. ^{1}H NMR spectra were recorded on Bruker Avance 400 HD spectrometer equipped with 5 mm pulsed-field-gradient (PFG) probes. The composition of the reaction products was analyzed by means of GC (Agilent 4890D) with an FID detector and a nonpolar capillary column (DB-5) (30 m × 0.25 mm × 0.25 μm). The GC-MS analysis was performed using gas chromatography-mass spectrometry (GC-MS, 7890A and 5975C, Agilent).

Supplementary Figures and Tables

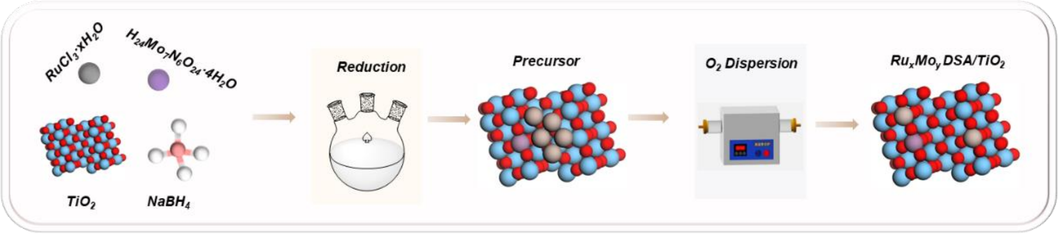

Supplementary Fig. 1 Schematic diagram of catalyst synthesis.

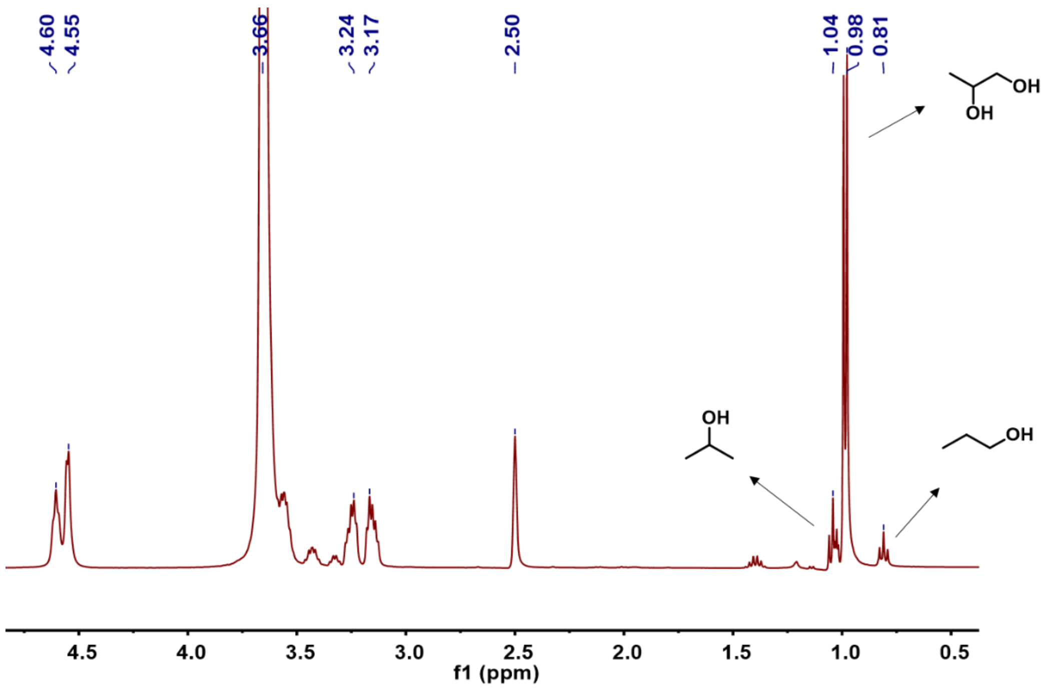

Supplementary Fig. 2 ^{1}H NMR spectrum of the reaction solution of PLA decomposition in the presence of H_2 in water using Ru_6/TiO_2 catalyst. Conditions: polylactic acid (PLA) 72 mg; Ru_6/TiO_2, 10 mg; H_2O, 0.5 mL; H_2 4 MPa, 160 °C, 12 h.

From the ^{1}H NMR spectrum, it is obvious that besides 1,2-propanediol, byproducts including 2-propanol and 1-propanol were obtained as well, indicating the hydrodeoxygenation of 1,2-propanediol occurred using Ru_6/TiO_2 under the experimental conditions.

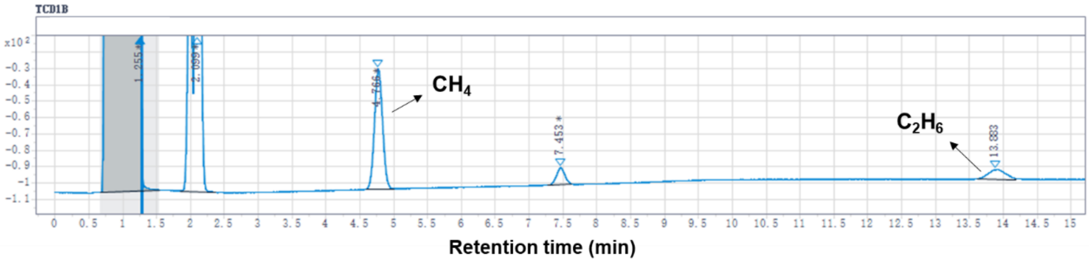

Supplementary Fig. 3 GC spectrum of the reaction solution of PLA decomposition in the presence of H_2 in water using Ru_6/TiO_2 catalyst. Conditions: PLA 72 mg; Ru_6/TiO_2, 10 mg; H_2O, 0.5 mL; H_2 4 MPa, 160 °C, 12 h.

From the GC spectrum, it is obvious that byproducts including methane and ethane were obtained, indicating that hydrogenolysis of 1,2-propanediol occurred using Ru_6/TiO_2 under the experimental conditions.

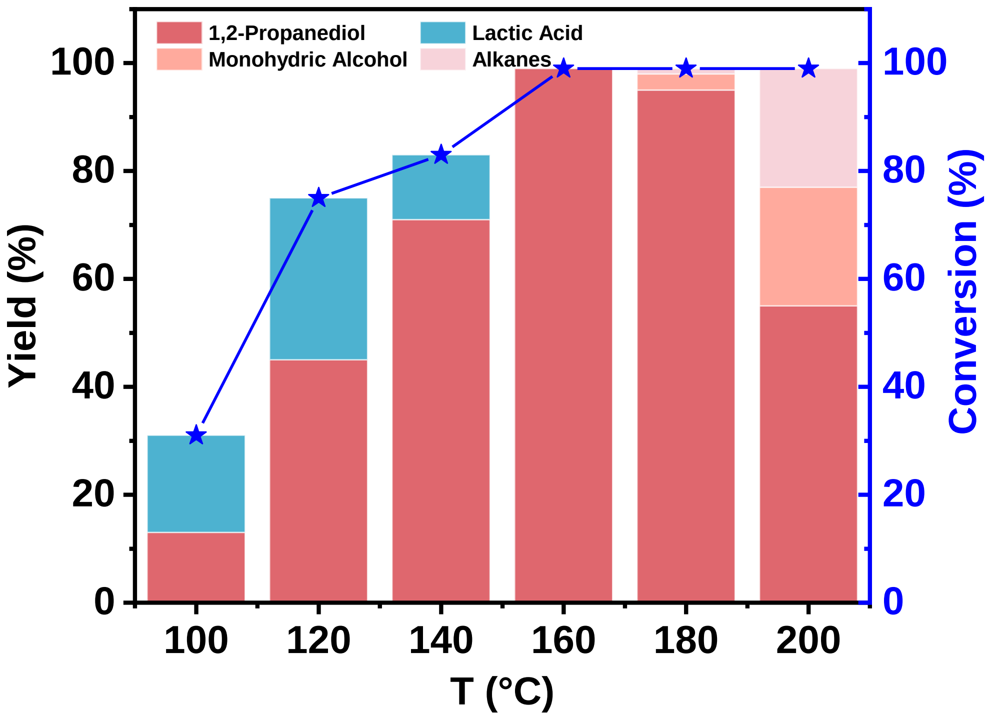

Supplementary Fig. 4 Temperature influence on the catalytic activity of Ru_4Mo_1/TiO_2 for PLA degradation. Reaction conditions: PLA 72 mg, Ru_4Mo_1/TiO_2 mg, H_2O 0.5 mL, H_2 4 MPa, 12 h.

Obviously, the decomposition of PLA could occur at a temperature of 100 ºC, and lactic acid (LA) and 1,2-propanediol were obtained in comparable yields. At this temperature, the hydrolysis of the polymer commenced, resulting in the formation of LA, which was simultaneously hydrogenated to produce 1,2-propanediol. The polymer hydrolysis and the subsequent LA hydrogenation was found to escalate with temperature. In the temperature of 100-160 ºC, only 1,2-propanediol was detected as the hydrogenated product, and at 160 ºC LA was hardly detectable. Increasing temperature to 180 ºC, byproducts including monohydric alcohols and alkanes were detected, resulting in decline in the yield of 1,2-propanediol. These findings underscore the influence of temperature on the degradation of PLA, providing valuable insights into the underlying mechanisms governing the transformation of PLA into desired products.

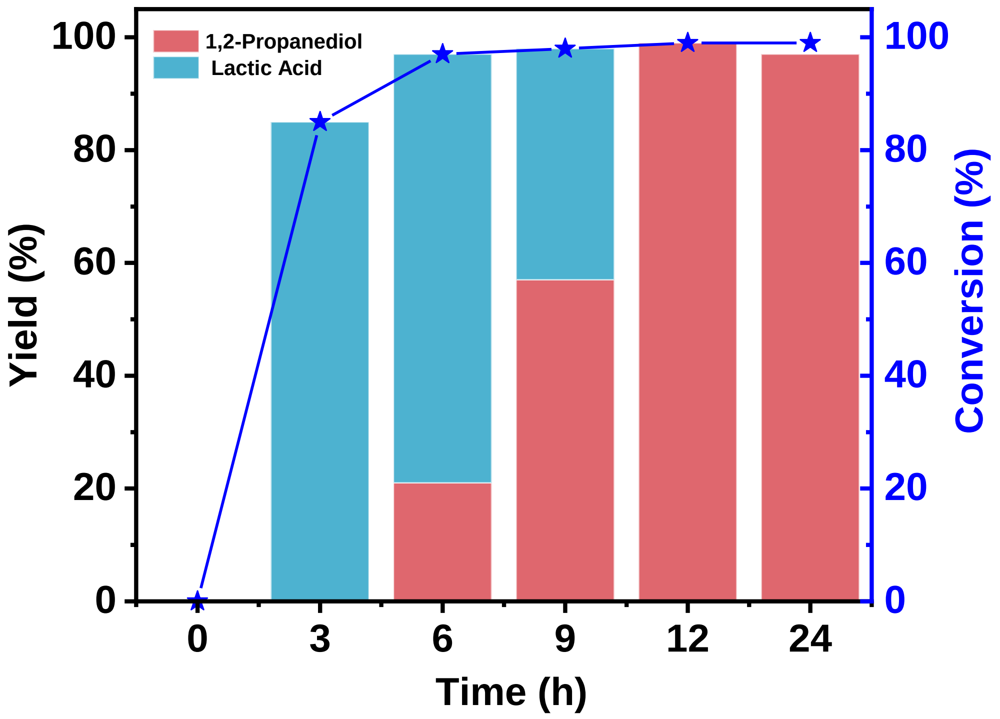

Supplementary Fig. 5 Reaction time influence on the catalytic activity of Ru_4Mo_1/TiO_2 for the PLA degradation. Reaction condition: PLA 1mmol, Ru_4Mo_1/TiO_2 10 mg, H_2O 0.5 ml, H_2 4 MPa, 160°C.

At this temperature, the hydrolysis of the polymer predominated initially, and a LA yield over 80% was achieved within 3 h, without 1,2-propanediol detectable. As the reaction time prolonged, the hydrogenation of LA ensued, yielding 1,2-propanediol as the sole hydrogenated product, and its yields increased with time. As reaction proceeded for 12 h, LA was hardly detectable, and the 1,2-propanediol yield approached 100%. Further prolonging time to 24 h, small amount of byproducts were obtained. These findings highlight the efficiency and selectivity of the hydrogenation process, underscoring the favorable reaction kinetics and the absence of undesired byproducts during the conversion of LA to 1,2-propanediol.

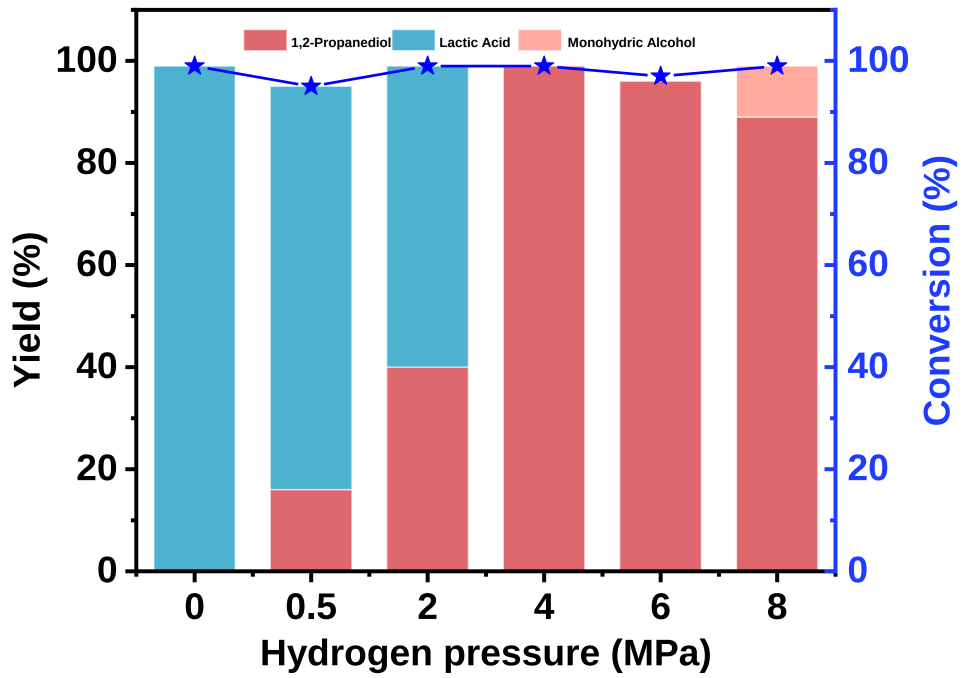

Supplementary Fig. 6 H_2 pressure influence on the catalytic activity of Ru_4Mo_1/TiO_2 for the PLA degradation. Reaction condition: PLA 1mmol, Ru_4Mo_1/TiO_2 10 mg, H_2O 0.5 mL, 160 °C, 12 h.

In the absence of hydrogen, the catalyst facilitated the thorough hydrolysis of PLA, leading to the complete conversion of PLA into LA. At low H_2 pressures (e.g., 0.5 MPa), the hydrogenation of LA occurred, and at an optimized pressure of 4 MPa, LA was completely converted into 1,2-propenadiol. However, as the H_2 pressure further increased beyond the optimum point, excessive hydrogenation took place, resulting in the formation of undesired by-products. These findings underscore the significance of precise control over H_2 pressure as a crucial parameter in achieving selective hydrogenation of LA while minimizing the occurrence of side reactions.

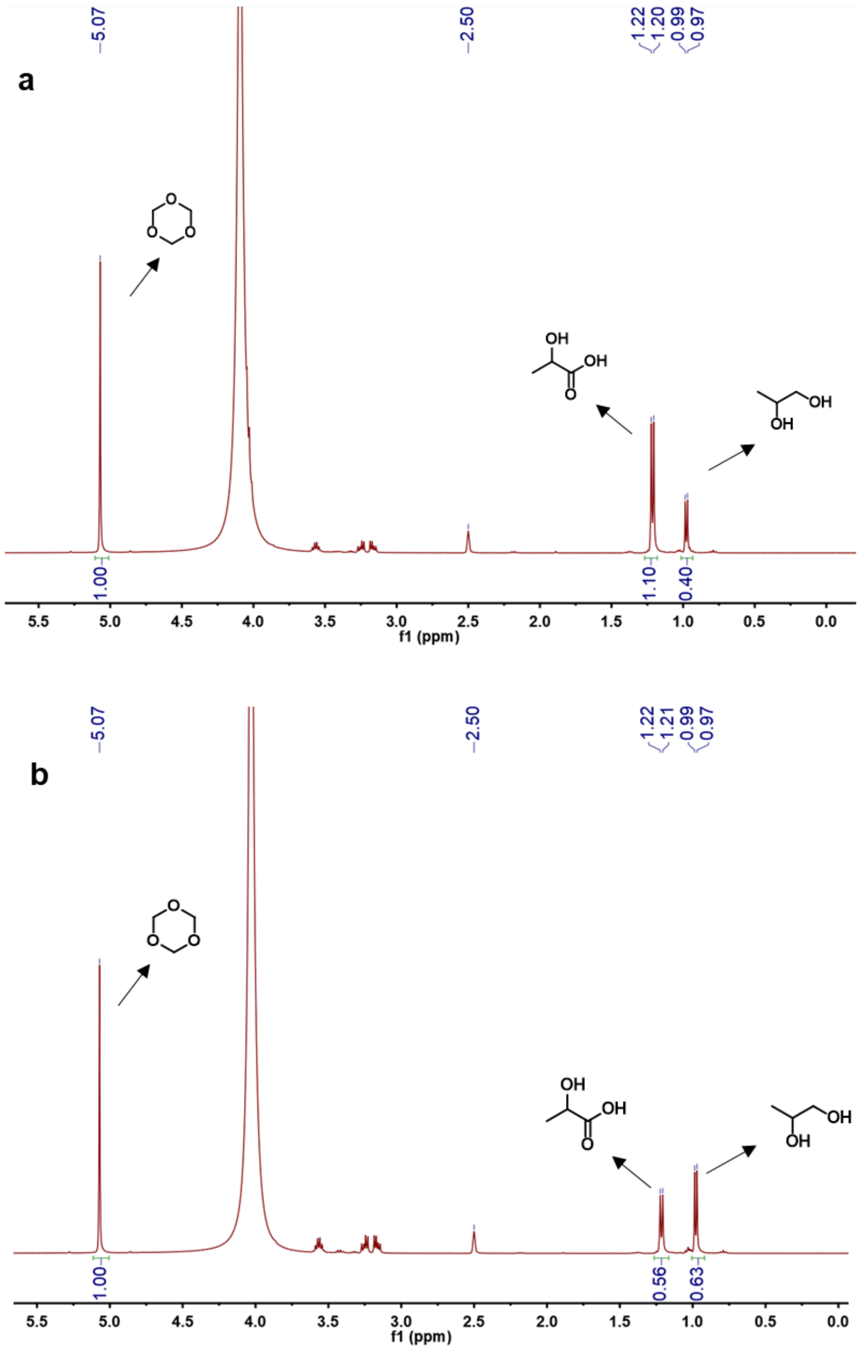

Supplementary Fig. 7 ^{1}H NMR spectra of the reaction solution of PLA depolymerization in the presence of H_2 in water using Ru_4Mo_1/TiO_2 under (a) 6 and (b) 9 hours. Conditions: PLA, 72 mg; Ru_4Mo_1/TiO_2, 10 mg; H_2O, 0.5 mL; H_2, 4 MPa; 160 °C. Paraformaldehyde was employed as an internal standard for ^{1}H NMR analysis. The cumulative yields of LA and 1,2-propanediol reached up to 100% within 6 h, indicating complete depolymerization of PLA into monomers. As the reaction proceeded for 9 h, the yield of LA decreased, while the yield of 1,2-propanediol increased accordingly, without any other discernible byproducts. These results strongly suggest that 1,2-propanediol was accessed through hydrogenation of LA, and no side reaction occurred.

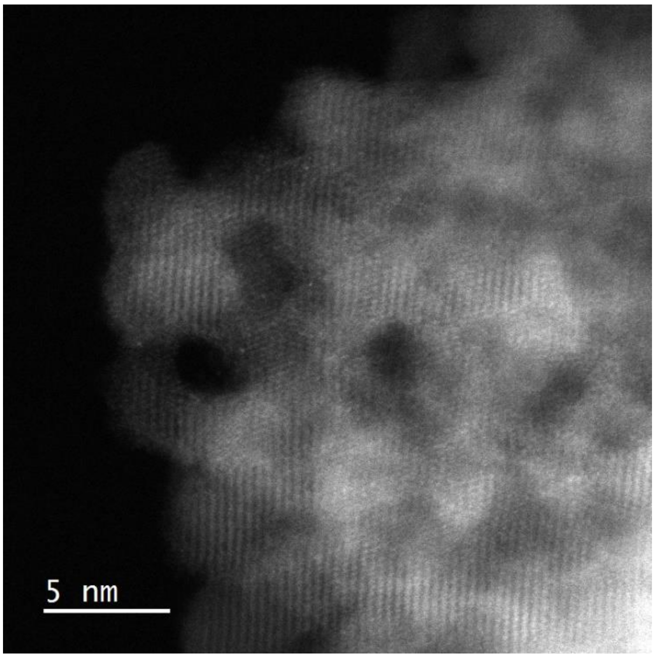

Supplementary Fig. 8 AC HAADF-STEM image of Mo_2/TiO_2.

This image provides compelling evidence that no visible MoO_x particles are observed in Mo_2/TiO_2. Furthermore, the presence of solitary bright spots in the image serves as a definitive indication that Mo was indeed atomically dispersed within the TiO_2 matrix.

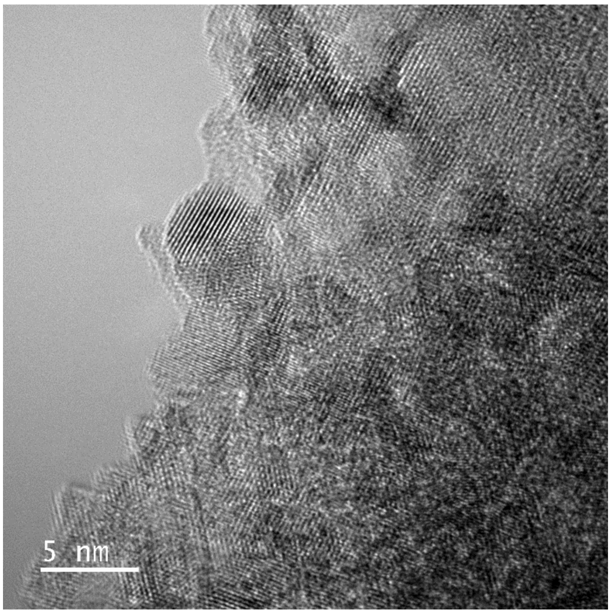

Supplementary Fig. 9 HR-TEM image of $Ru_4Mo_{0.1}/TiO_2$.

This image reveals the absence of conspicuous Ru and MoOx particles in $Ru_4Mo_{0.1}/TiO_2$. No discernible alterations in the lattice of TiO_2 nanoparticles were observed. These findings offer preliminary evidence on the atomic-level dispersion of Ru and Mo in the catalyst.

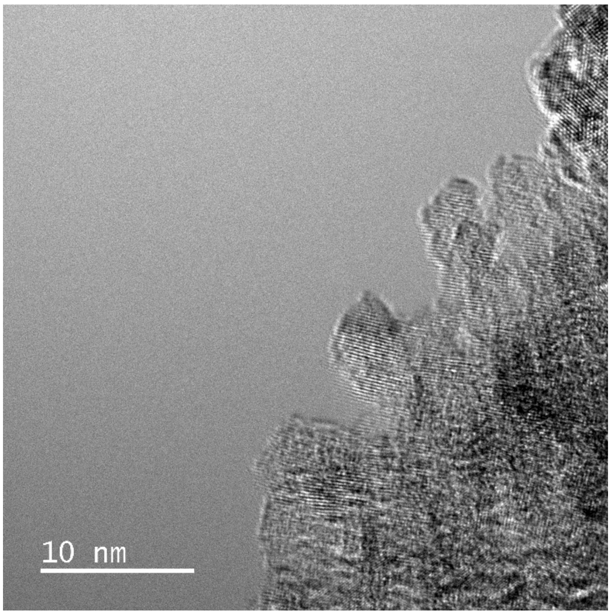

Supplementary Fig. 10 HR-TEM image of Ru_2Mo_1/TiO_2.

This image reveals the absence of conspicuous Ru and MoOx particles in Ru_2Mo_1/TiO_2. No discernible alterations in the lattice of TiO_2 nanoparticles were observed. These findings offer preliminary evidence on the atomic-level dispersion of Ru and Mo in the catalyst.

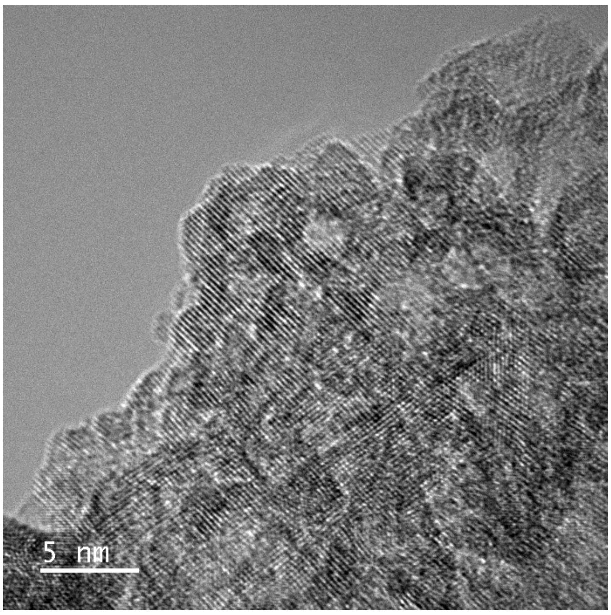

Supplementary Fig. 11 HR-TEM image of Ru_3Mo_{10}/TiO_2.

This image reveals the absence of conspicuous Ru and MoOx particles on Ru_3Mo_{10}/TiO_2. No discernible alterations in the lattice of TiO_2 nanoparticles were observed. These findings offer preliminary evidence on the atomic-level dispersion of Ru and Mo in the catalyst.

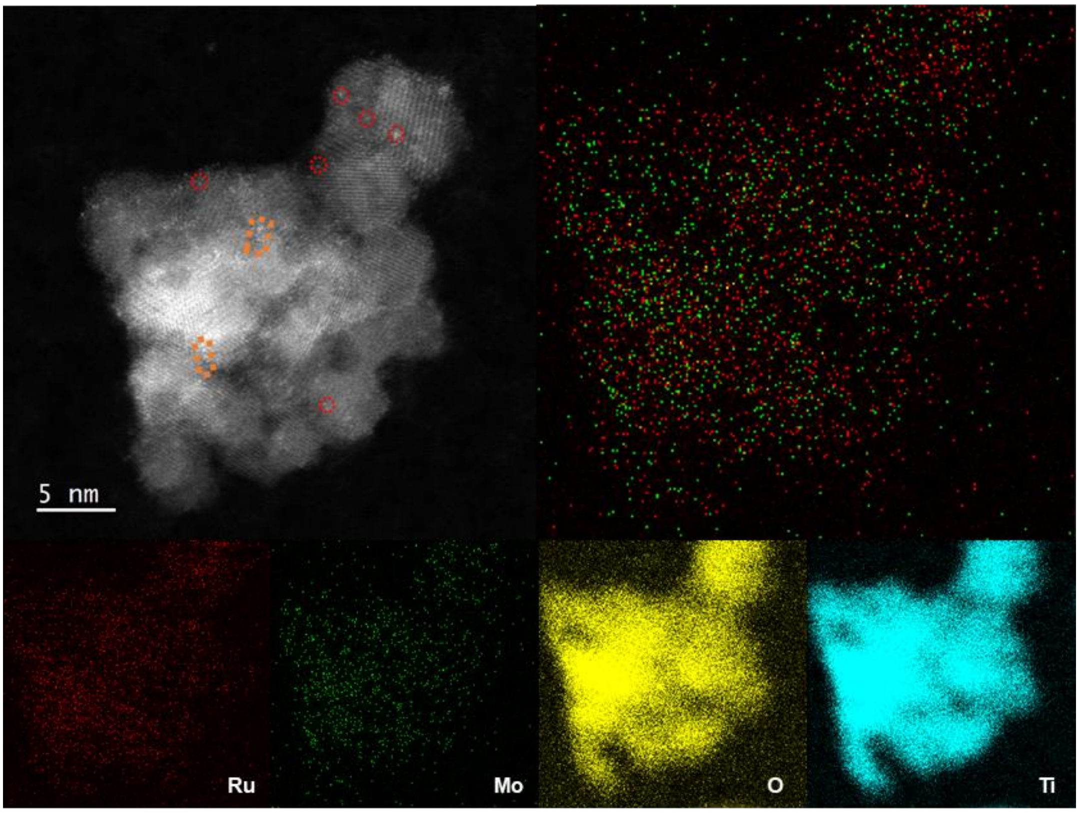

Supplementary Fig. 12 AC HAADF-STEM image and EDS mapping of Ru_4Mo_1/TiO_2. AC HAADF-STEM and EDS mapping were used to provide the information on element distribution of Ru_4Mo_1/TiO_2. This image provides compelling evidence that no particles are present in Ru_4Mo_1/TiO_2. Furthermore, solitary bright spots in the AC HAADF-STEM image indicate that Ru and Mo was indeed atomically dispersed on the TiO_2 support.

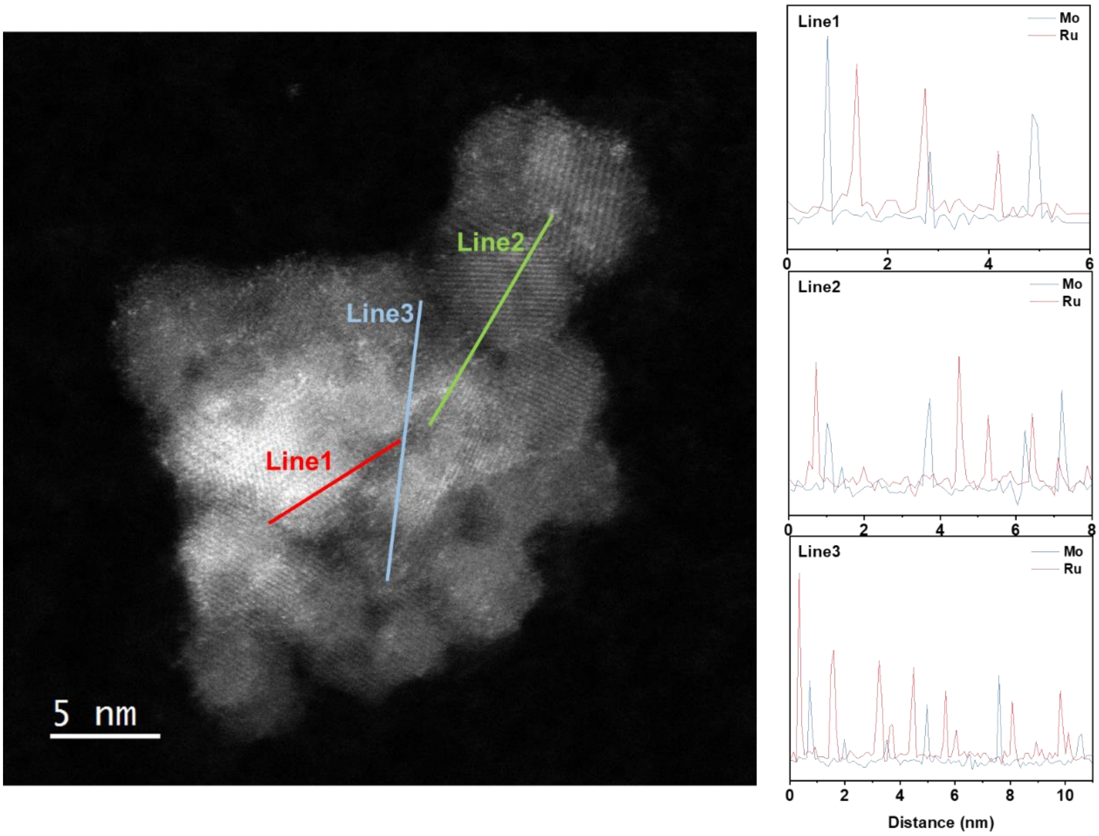

Supplementary Fig. 13 EDS line-scan of Ru_4Mo_1/TiO_2.

The line-scan results show that both Ru SAs and Ru-Mo DAs sites are present in the catalyst.

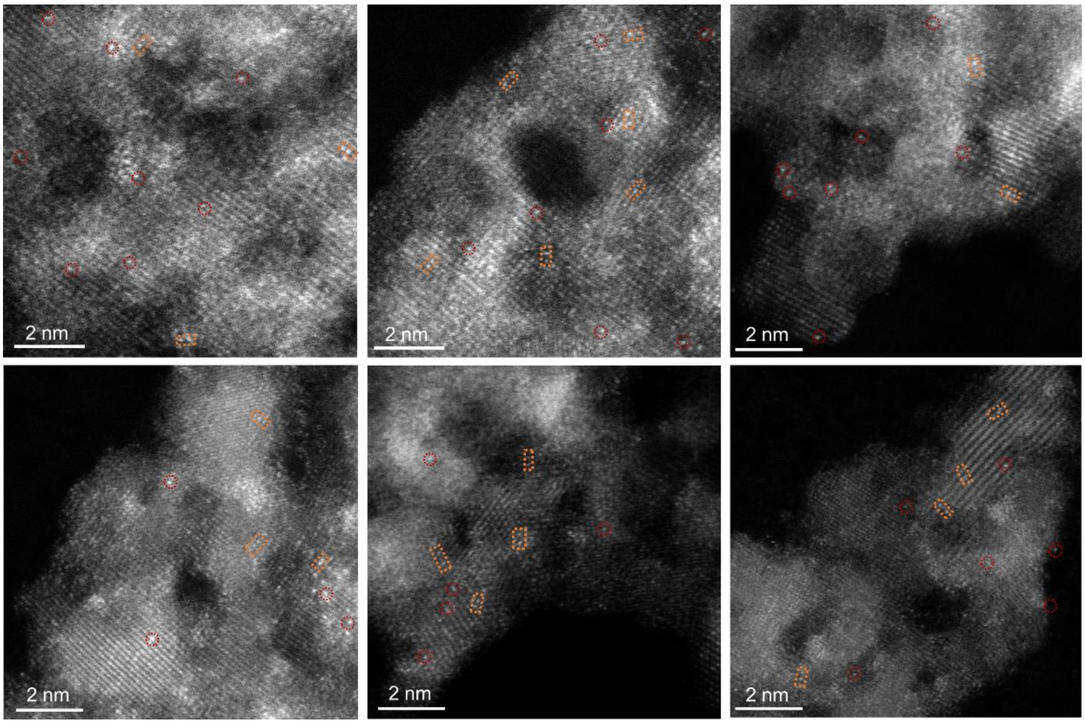

Supplementary Fig. 14 AC HAADF-STEM image of Ru_4Mo_1/TiO_2.

To provide a more comprehensive illustration of the formation of Ru-O-Mo DAs and Ru SAs, Ru_4Mo_1/TiO_2 was observed by AC HAADF-STEM from multiple angles and locations. These images provide solid evidences on the coexistence of Ru SAs and Ru-Mo DAs.

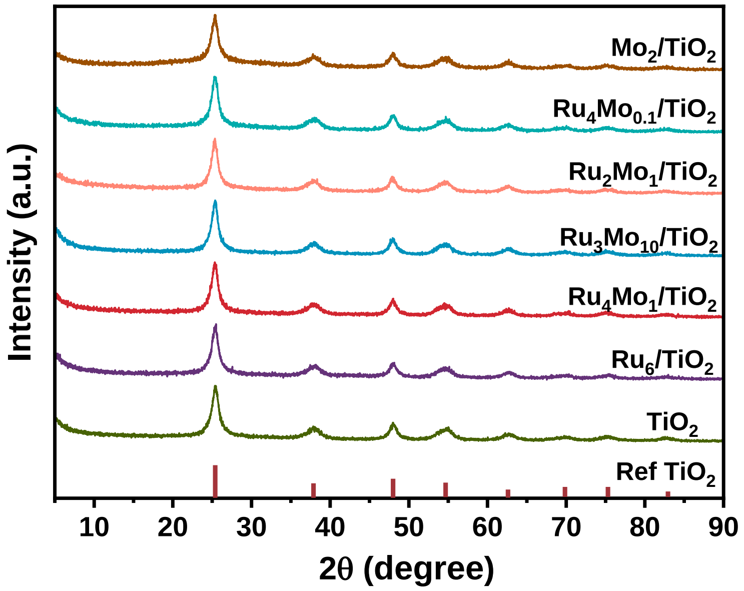

Supplementary Fig. 15 XRD patterns of the catalysts.

All the as-prepared samples showed similar XRD patterns to the TiO_2 support, without the signals assigning to Ru or Mo species, suggesting that there is no crystalline Ru or Mo species in these samples.

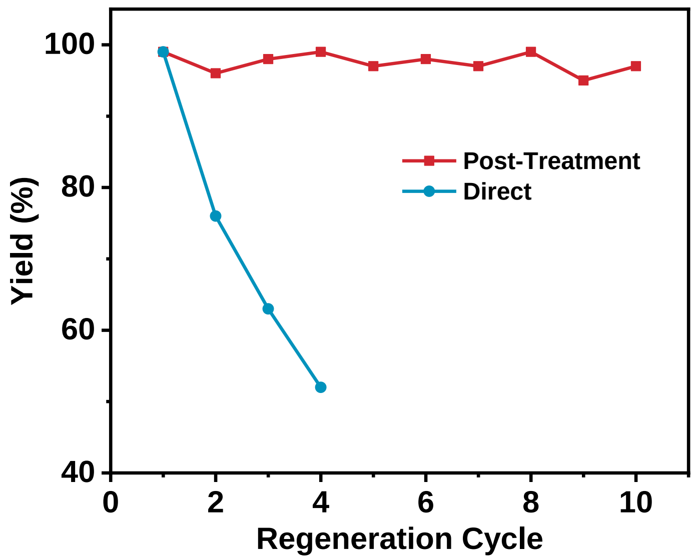

Supplementary Fig. 16 The chemical upcycling performance of Ru_4Mo_1/TiO_2 under the PLA decomposition in the presence of H_2 in water. Conditions: polylactic acid (PLA), 72 mg; Ru_4Mo_1/TiO_2, 10 mg; H_2O, 0.5 mL; H_2 4 MPa, 160 °C, 12 h.

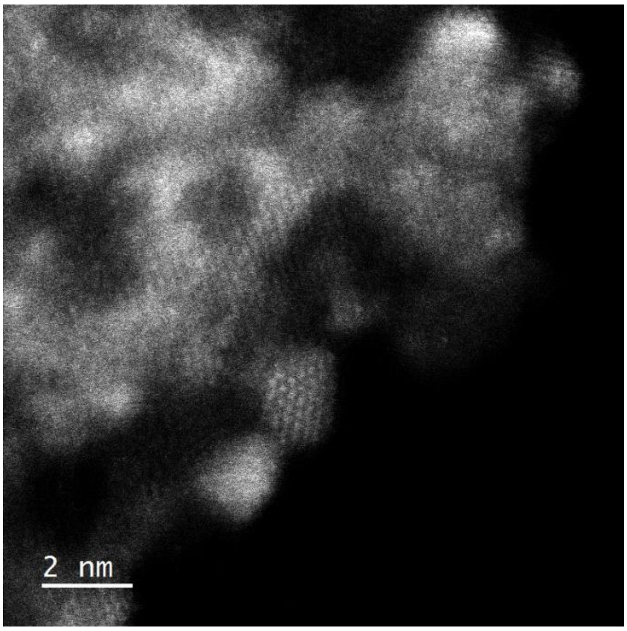

Supplementary Fig. 17 AC HAADF-STEM of the used Ru_4Mo_1/TiO_2 for PLA degradation in the presence of H_2 in water.

From the image, besides single atoms, some clusters or particles with size around 2.0 nm were observed. This means that some Ru SAs in the Ru_4Mo_1/TiO_2 aggregated into nanoparticles in the reaction process.

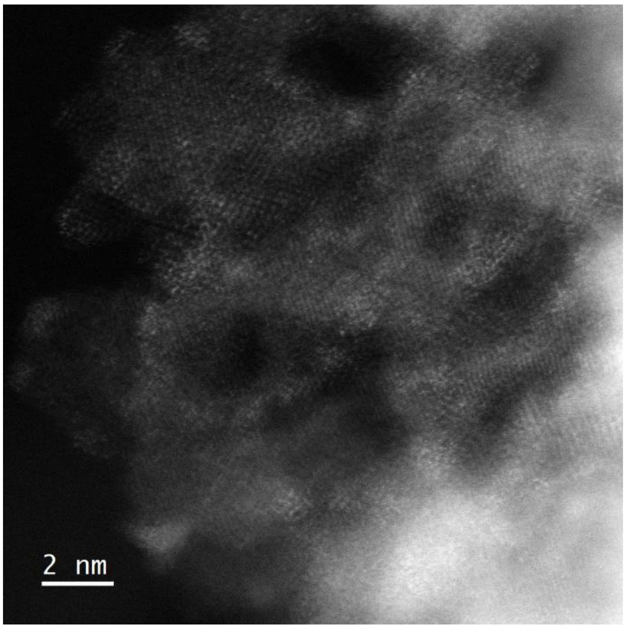

Supplementary Fig. 18 AC HAADF-STEM of the used Ru_4Mo_1/TiO_2 after regeneration. From the image, it is clear that there is no Ru clusters or particles, meaning that the formed Ru clusters or particles were converted into SAs in the post-treatment in O_2 atmosphere. This can explain why the regenerated Ru_4Mo_1/TiO_2 displayed the similar activity to the refresh one.

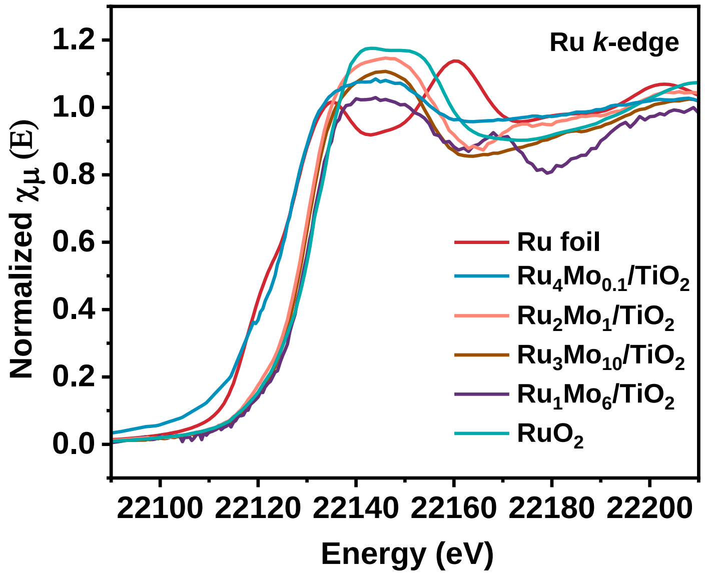

Supplementary Fig. 19 Ru K-edge X-ray absorption near-edge structure (XANES) spectra.

The rising edges of the Ru_xMo_y/TiO_2 spectra lay between those of Ru foil and RuO_2, indicating that the valences of Ru in the catalysts are in the range of 0 ~ +4. As the Mo:Ru ratio increases, a noticeable upward shift of the near-edge absorption energies is observed, indicating a progressive increase in the valence state of Ru. This compelling observation serves as compelling evidence, substantiating the pivotal role played by the addition of Mo in facilitating the oxidation of Ru.

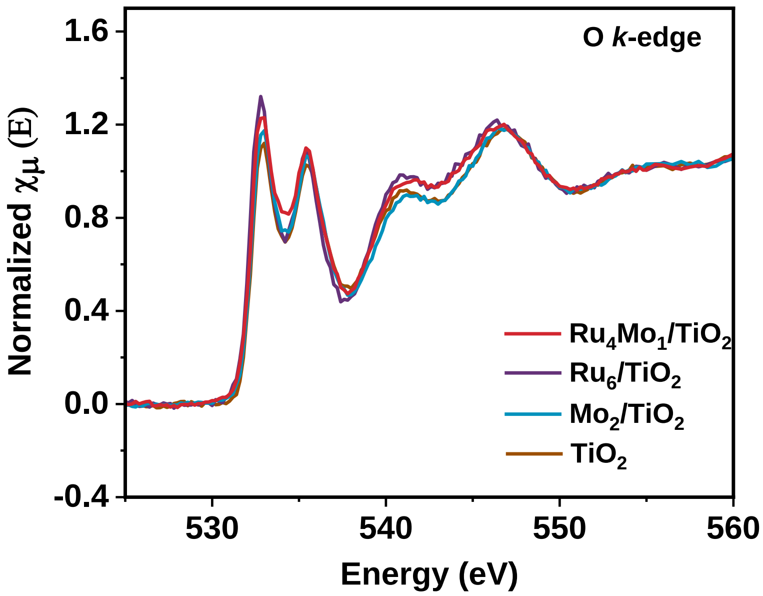

Supplementary Fig. 20 Soft X-ray absorption spectra of Ru_4Mo_1/TiO_2, Ru/TiO_2, Mo_2/TiO_2, and TiO_2.

These soft X-ray absorption spectra indicate that the absorption intensity of the edge-front peak of Ru_4Mo_1/TiO_2 decreased compared to that of Ru_6/TiO_2, suggesting an elevated valence state of surface oxygen in Ru_4Mo_1/TiO_2.

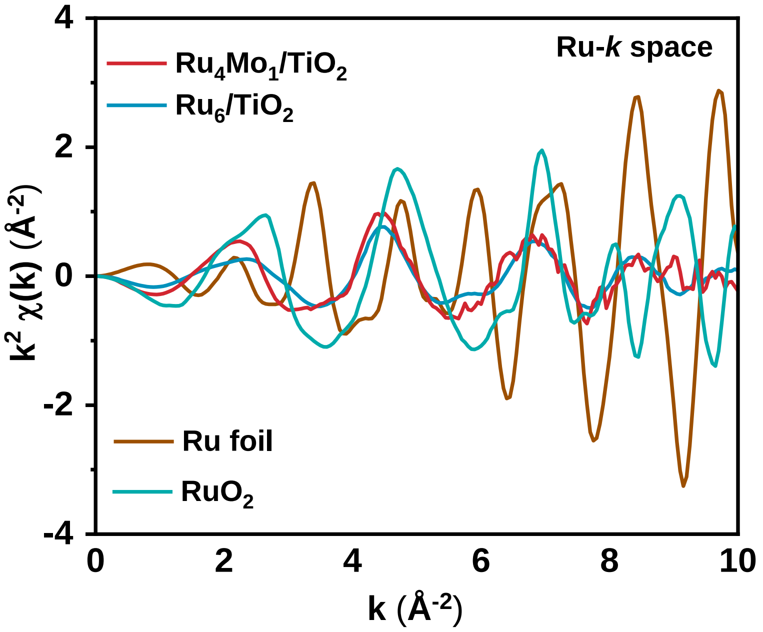

Supplementary Fig. 21 K^2 -weight EXAFS spectra of catalysts and Ru references. K^2 -weight EXAFS spectra of Ru_4Mo_1/TiO_2 and Ru_6/TiO_2 matched the *R*-space results in the manuscript (Fig. 3c).

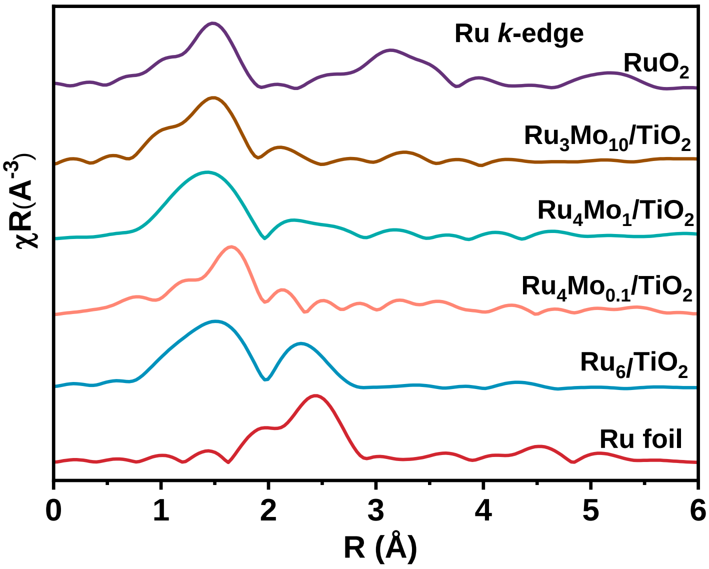

Supplementary Fig. 22 Ru K-edge *R*-space X-ray absorption near-edge structure spectra.

The strong peak at 1-2 Å in R-space corresponds to the first coordination shell of Ru-O bond. The Ru foil exhibits a peak situated within 2-3 Å ascribed to the Ru-Ru bond. The fitting results show that partial oxidation of Ru occurs in the Ru_6/TiO_2 catalyst. However, the presence of Ru-Ru bond gives rise to a discernible nanoparticle structure. In contrast, all the Ru_xMo_y/TiO_2 samples exclusively exhibit Ru-O bond, suggesting the O-stabilized Ru SA dispersion throughout the catalysts.

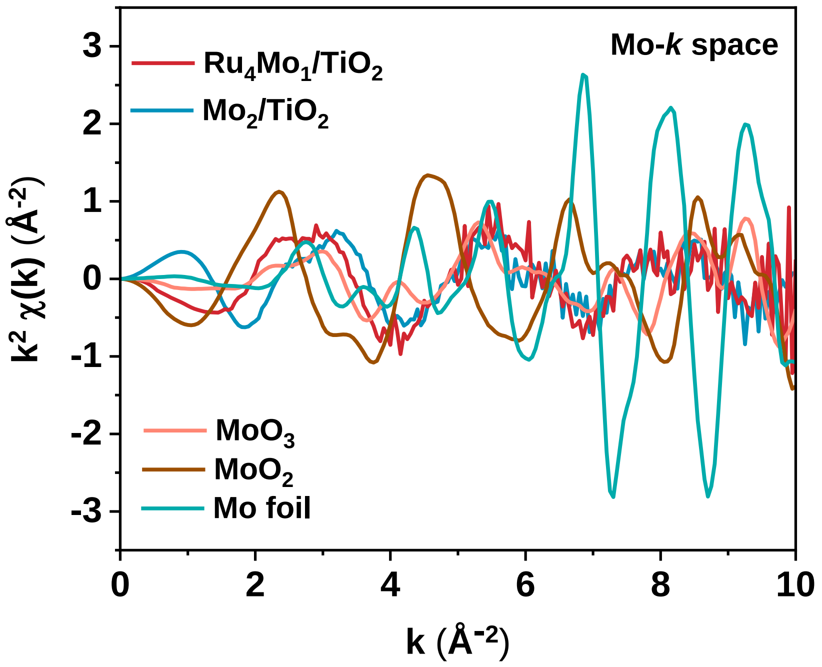

Supplementary Fig. 23 K^2 -weight EXAFS spectra of catalysts and Mo references. K^2 -weight EXAFS spectra of Ru_4Mo_1/TiO_2 and Mo_2/TiO_2 matched the *R*-space results in the manuscript (Fig. 3d).

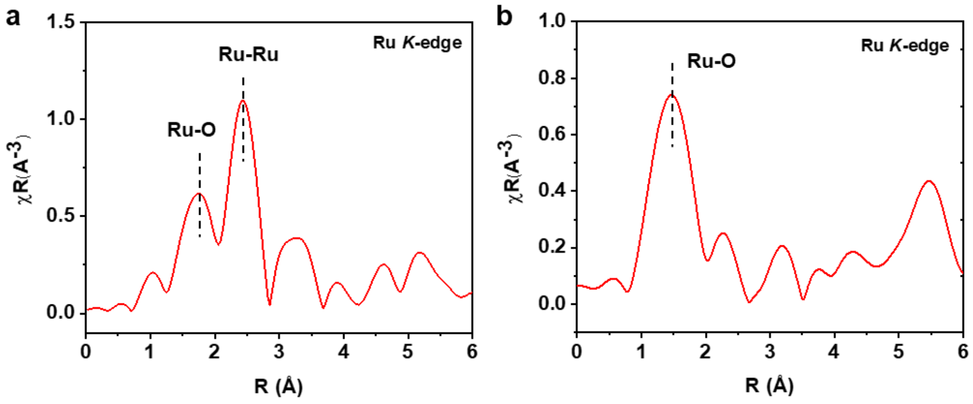

Supplementary Fig. 24 Ru K-edge *R*-space X-ray absorption near-edge structure spectra of (a) used and (b) regenerated catalyst.

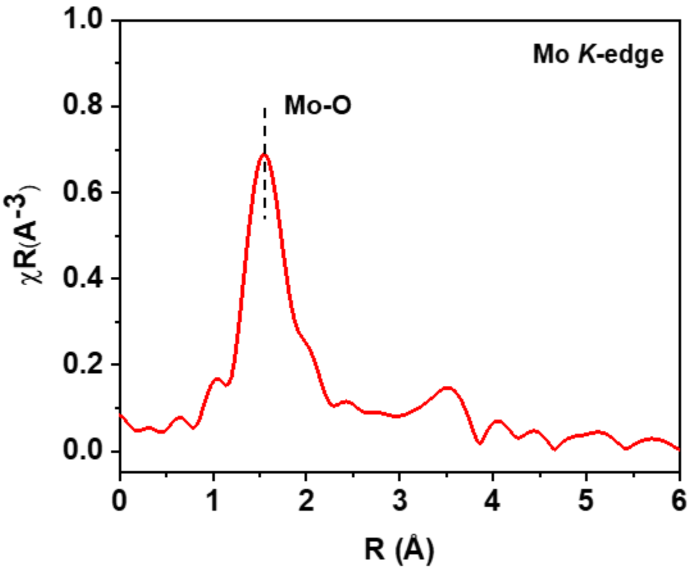

Supplementary Fig. 25 Mo K-edge *R*-space X-ray absorption near-edge structure spectrum of used catalyst.

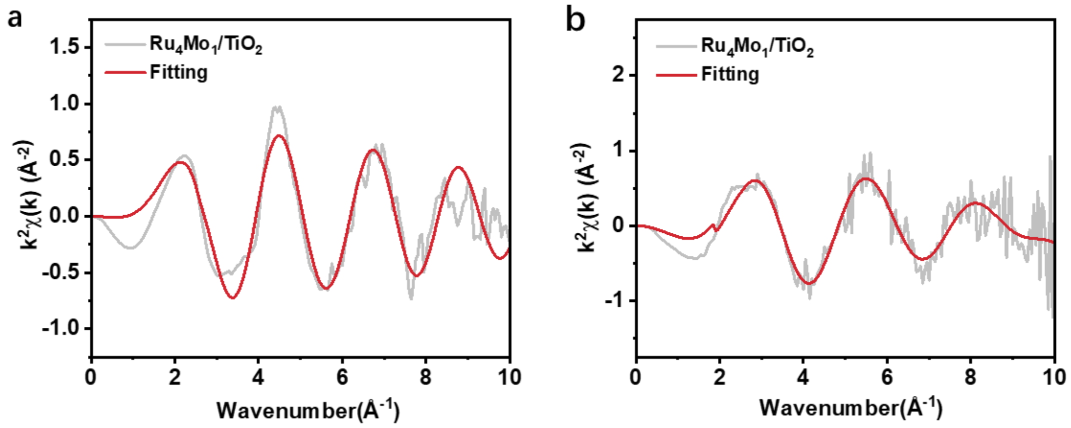

Supplementary Fig. 26 ***K*-space EXAFS fitting for (a) Ru and (b) Mo of Ru_4Mo_1/TiO_2.**

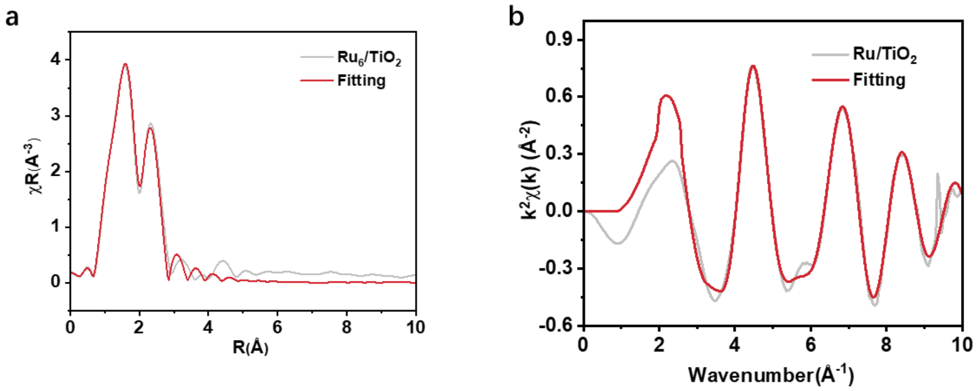

Supplementary Fig. 27 (a) *R*-space and (b) *k*-space EXAFS fitting for Ru of Ru_6/TiO_2.

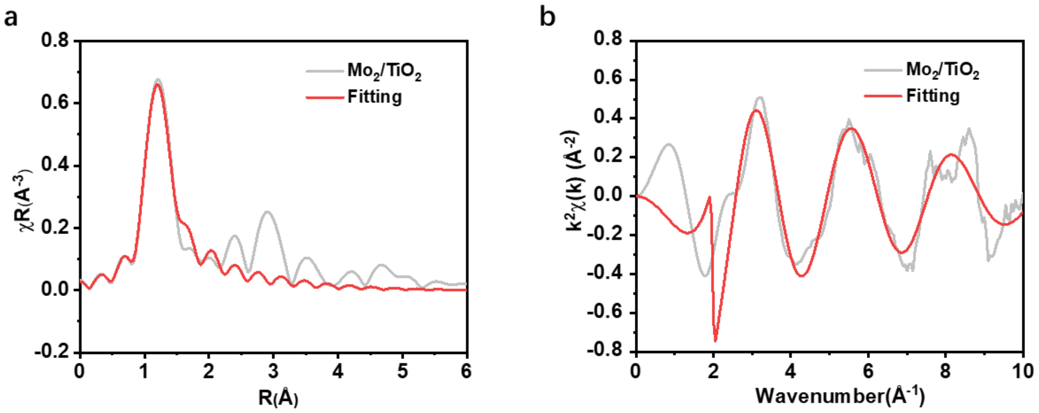

Supplementary Fig. 28 (a) *R*-space and (b) *k*-space EXAFS fitting for Mo of Mo_2/TiO_2.

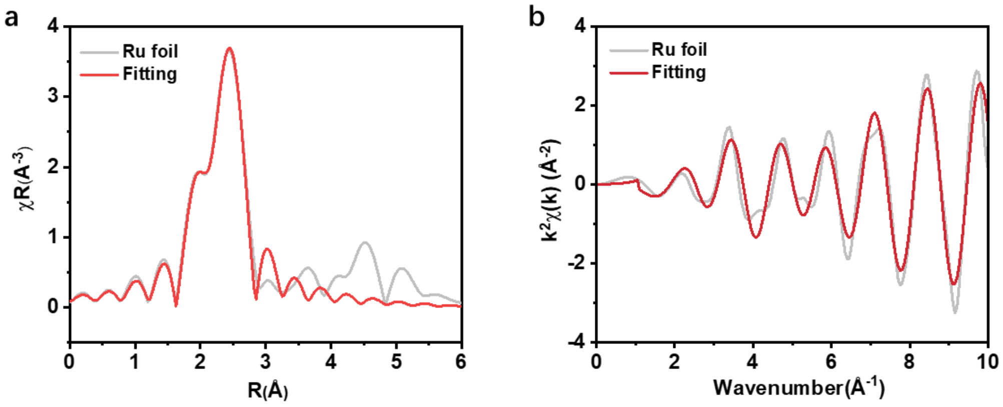

Supplementary Fig. 29 (a) *R*-space and (b) *k*-space EXAFS fitting for Ru of Ru foil.

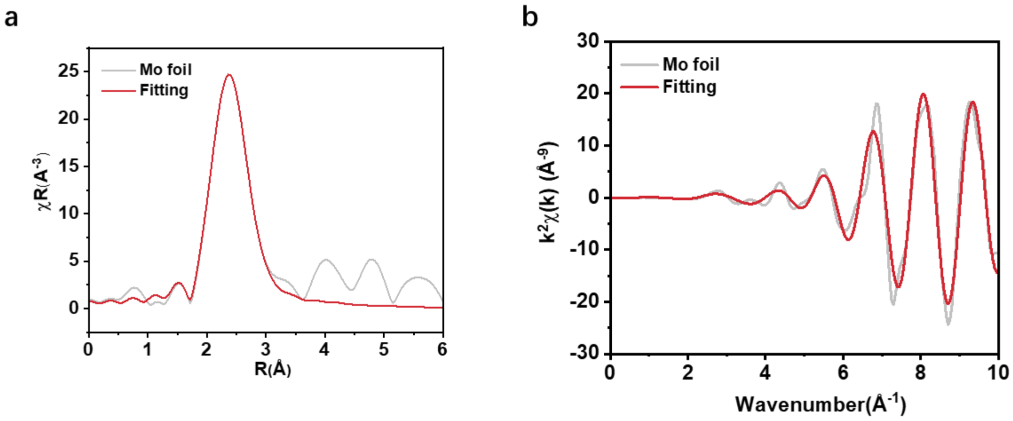

Supplementary Fig. 30 (a) *R*-space and (b) *k*-space EXAFS fitting for Mo of Mo foil.

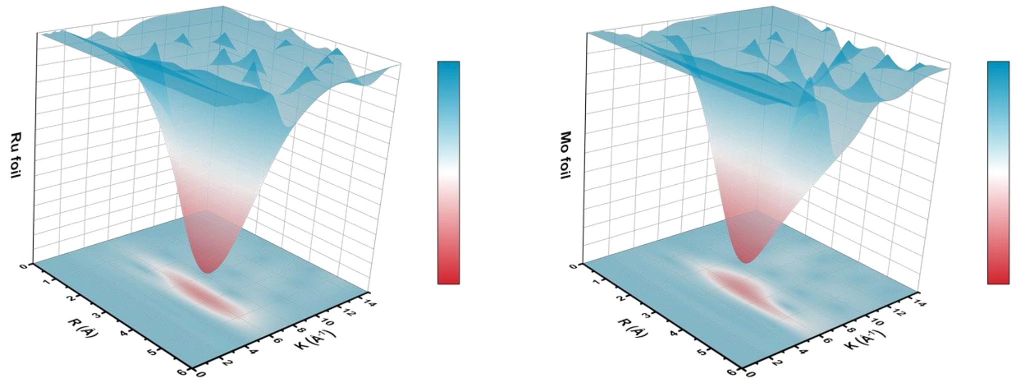

Supplementary Fig. 31 3D contour maps of WT-XAFS spectra for Ru foil and Mo foil. From the Wavelet Transform analysis, the χ(k) exported from Athena was imported into the Hama Fortran code. The parameters were listed as follow: *R* range, 0~6 Å, *k* range, 0~15 $Å^{-1}$ for samples; k weight, 2; and Morlet function with κ = 10, σ = 1 was used as the mother wavelet to provide the overall distribution.

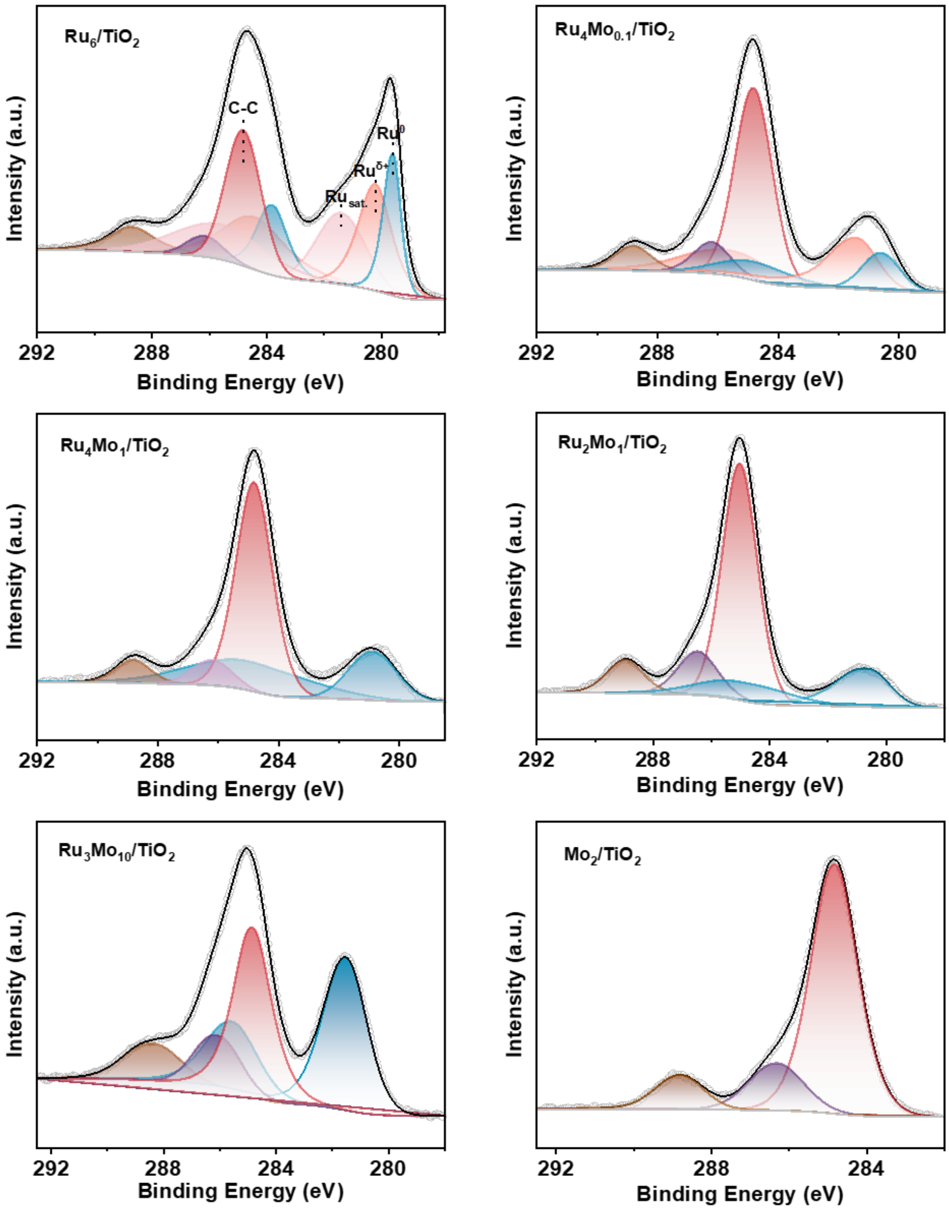

Supplementary Fig. 32 C *1s* and Ru *3d* XPS spectra of the resultant samples. The C-C peak was calibrated to 284.8 eV.

The Ru *3d* XPS spectrum shows binding energies (BEs) at 279.6, 280.2 and 281.4 eV, which are assigned to Ru^0, $Ru^{\delta+}$ and Ru sat., respectively. With the progressive increase in Mo loading, the emergence of $Ru^{\delta+}$ exclusively at 280.6 eV signifies the successful dispersion of Ru in its single-atomic form. Conversely, as the Ru:Mo ratio decreases, there is a gradual rise in the BEs to 281.5 eV, indicative of the efflux of Ru electrons.

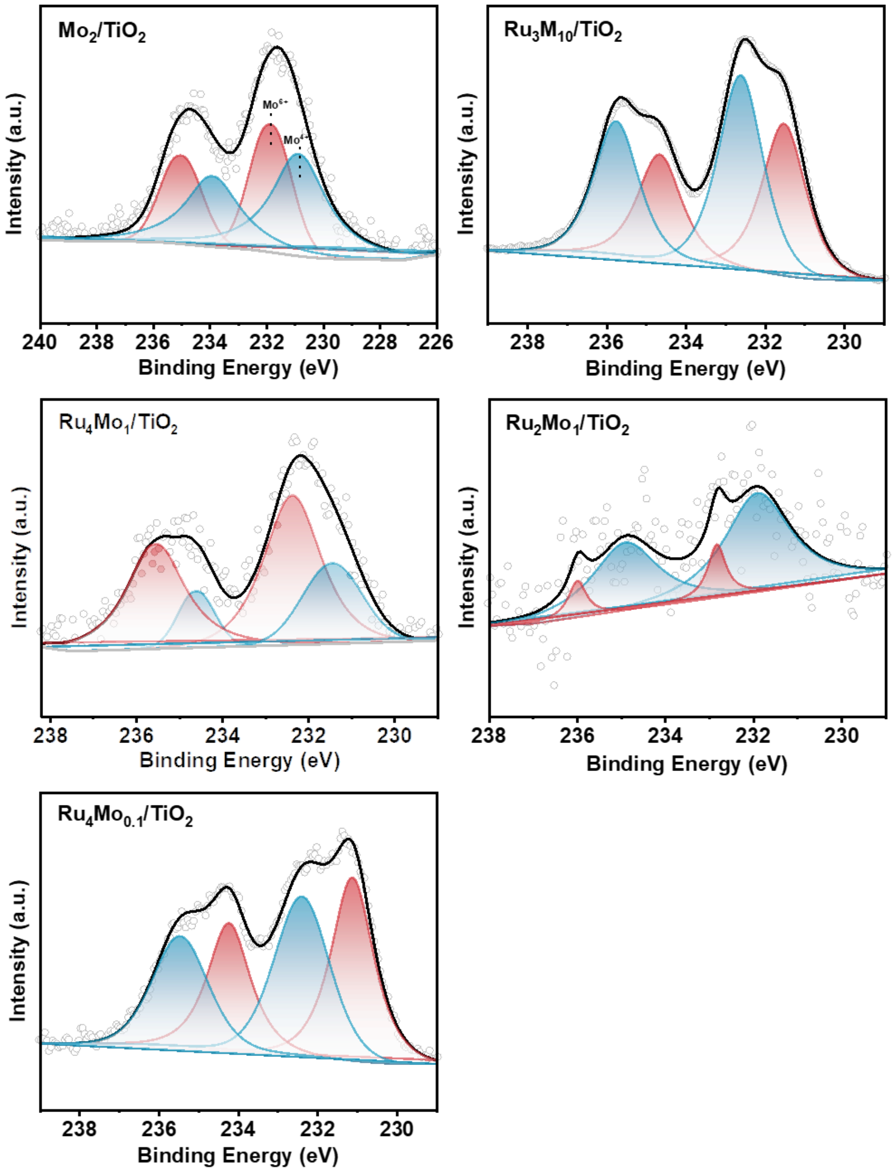

Supplementary Fig. 33 Mo *3d* XPS spectra of the resultant samples.

The Mo^{6+} and Mo^{4+} XPS spectra of Mo_2/TiO2 show binding energies at 231.82 and 230.84 eV, respectively, indicating that Mo in this sample exists in an oxide state. As the Ru:Mo ratio increases, there is a gradual rise in the BEs to 232.38 and 231.11 eV, respectively, ascribing to the strong electronic interactions between Ru and Mo.

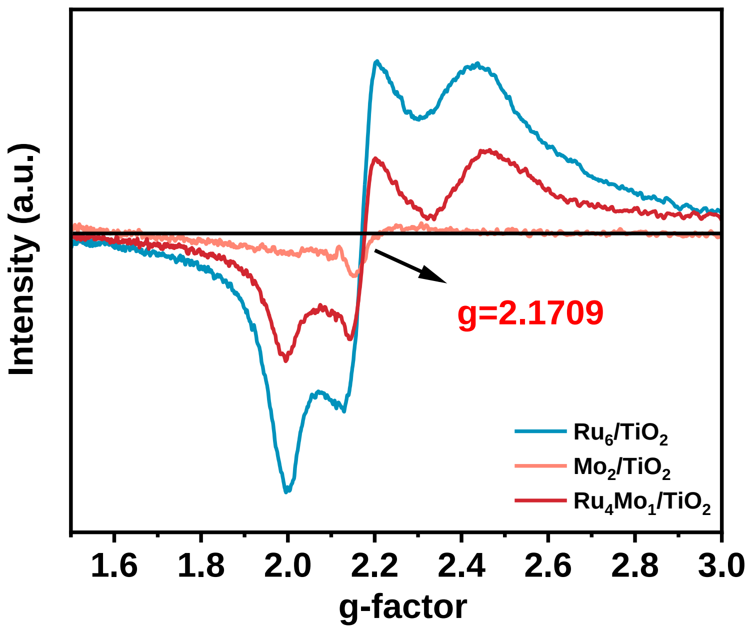

Supplementary Fig. 34 EPR spectra of Ru_6/TiO_2, Mo_2/TiO_2 and Ru_4Mo_1/TiO_2.

EPR (Electron Paramagnetic Resonance) *g*-value variations represent the changes in the electronic environment and magnetic properties of a system. The measured g-values of Ru_6/TiO_2, Mo_2/TiO_2, and Ru_4Mo_1/TiO_2 were determined to be 2.1767, 2.2151, and 2.1709, respectively. The obvious decrease in the g-values signifies a modification in the spin state of the electrons, implying that the Ru electrons experience alterations due to interactions between electrons, electron spin coupling, or electron spin-orbit interactions. These changes could potentially arise from localized electric-field effects associated with Mo.

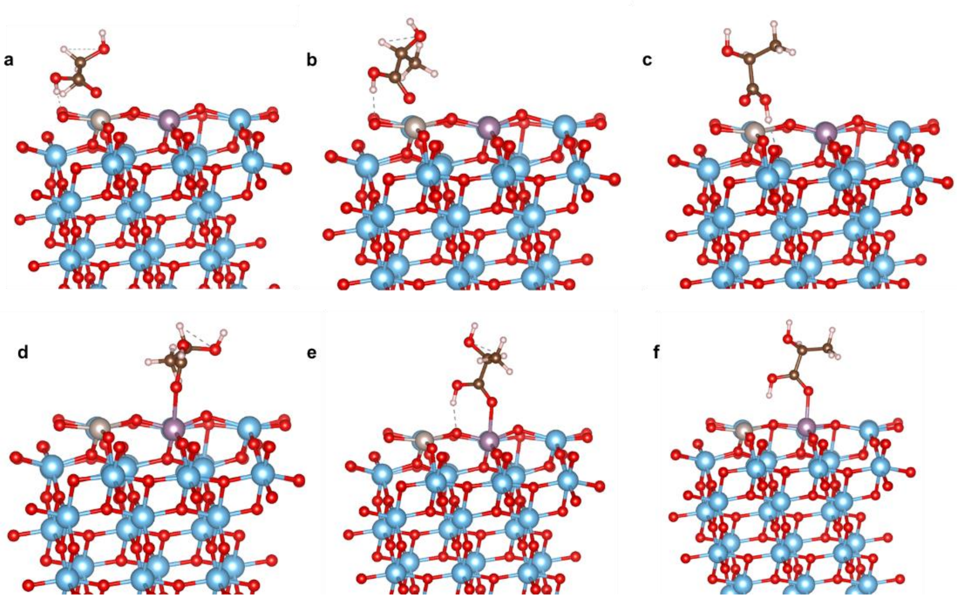

Supplementary Fig. 35 DFT calculation-optimized configurations of LA on Ru_4Mo_1/TiO_2. Gray: Ru, purple: Mo, red: O, blue: Ti and pink: H.

The adsorption energies of the LA on the catalyst surface are -0.59 eV (a), -0.49 eV (b), -0.89 eV (c), -1.09 eV (d), -1.12 eV (e), and -1.76 eV (f), respectively. It is indicated that LA is preferentially absorbed at the Mo site of Ru_4Mo_1/TiO_2.

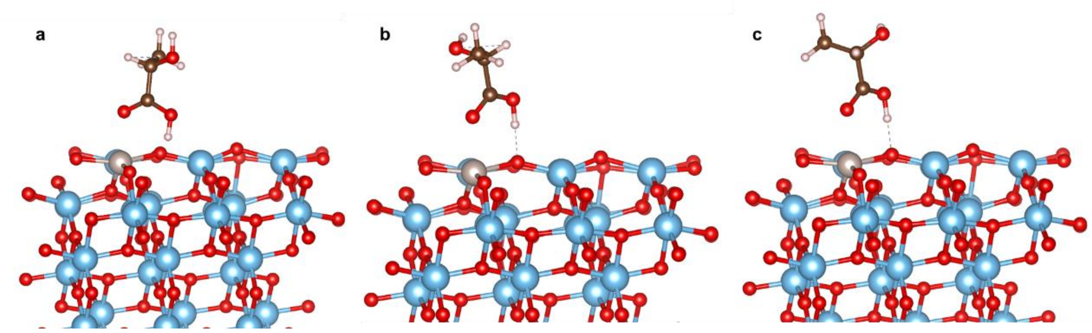

Supplementary Fig. 36 Screening of the adsorption structure of LA on the Ru SA site. Gray: Ru, purple: Mo, red: O, blue: Ti and pink: H.

The adsorption energies of the LA on the different sites of the catalyst surface are -1.30 eV (a), -1.38 eV (b), and -1.40 eV (c), respectively. It is indicated that LA forms hydrogen bonds with the lattice oxygen, but cannot bond effectively with Ru SA.

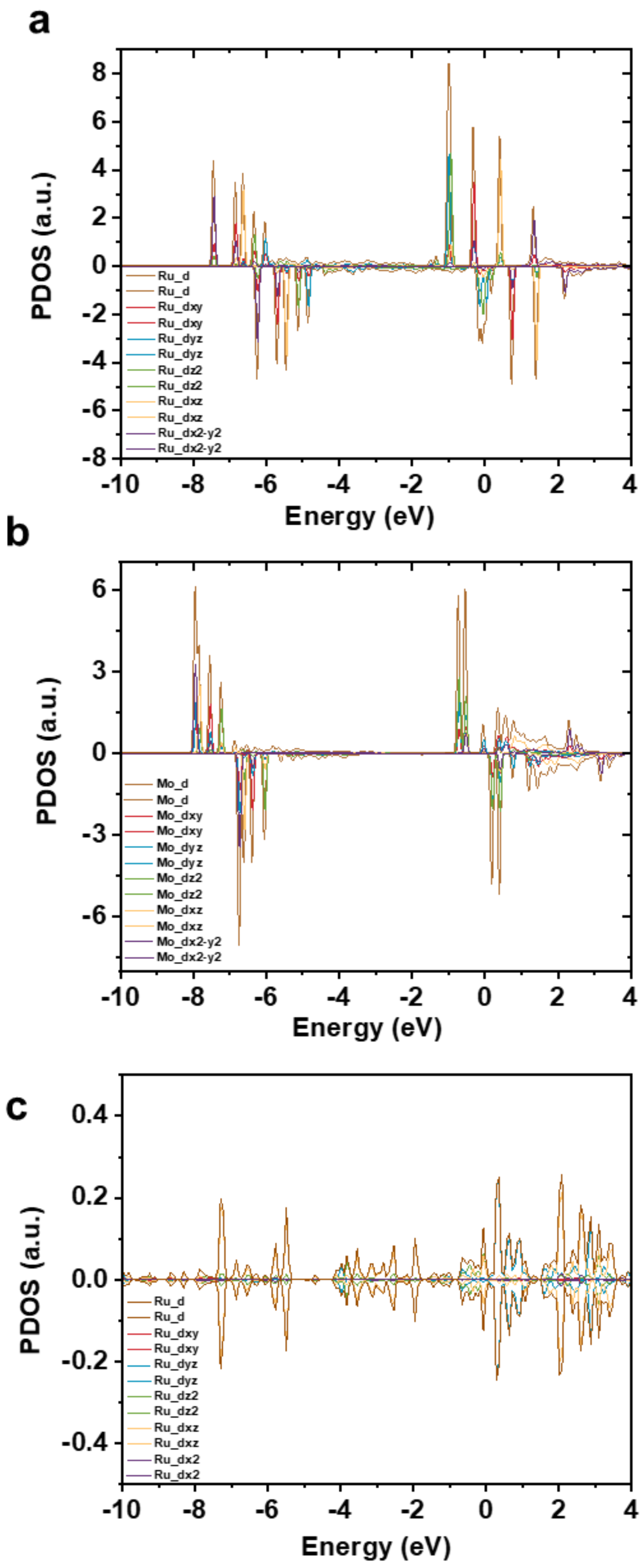

Supplementary Fig. 37 PDOS of (a) Ru *4d* in Ru SA/TiO_2, (b) Mo *4d* in the Mo SA/TiO_2 and (c) Ru *4d* in Ru_6/TiO_2.

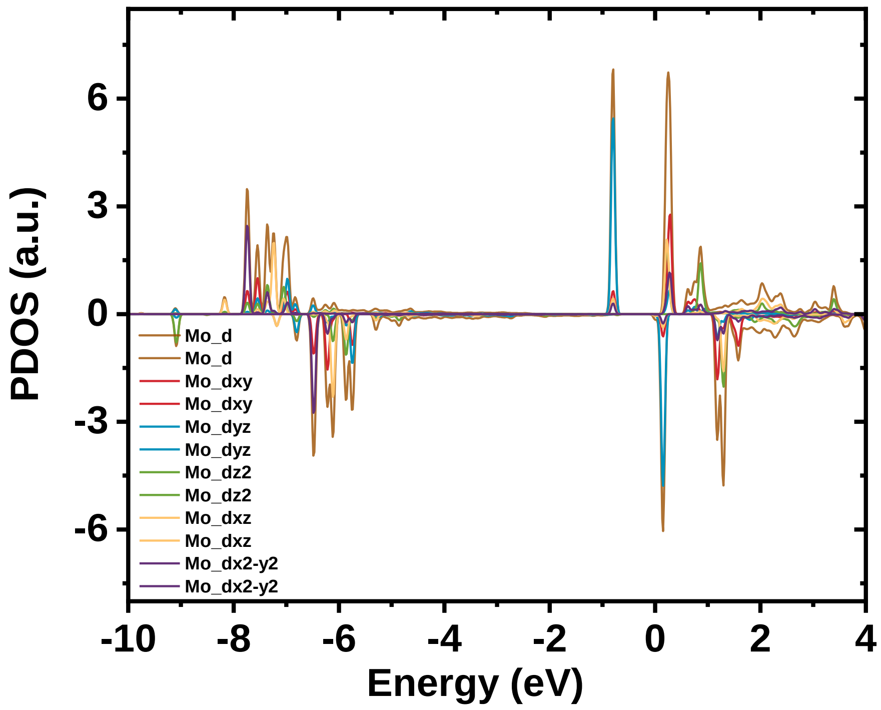

Supplementary Fig. 38 PDOS of Mo *4d* in Mo SA/TiO_2 after LA adsorption.
The distribution of *4d* orbitals before and after the adsorption of LA changed significantly, indicating that Mo SA adsorbs LA well.

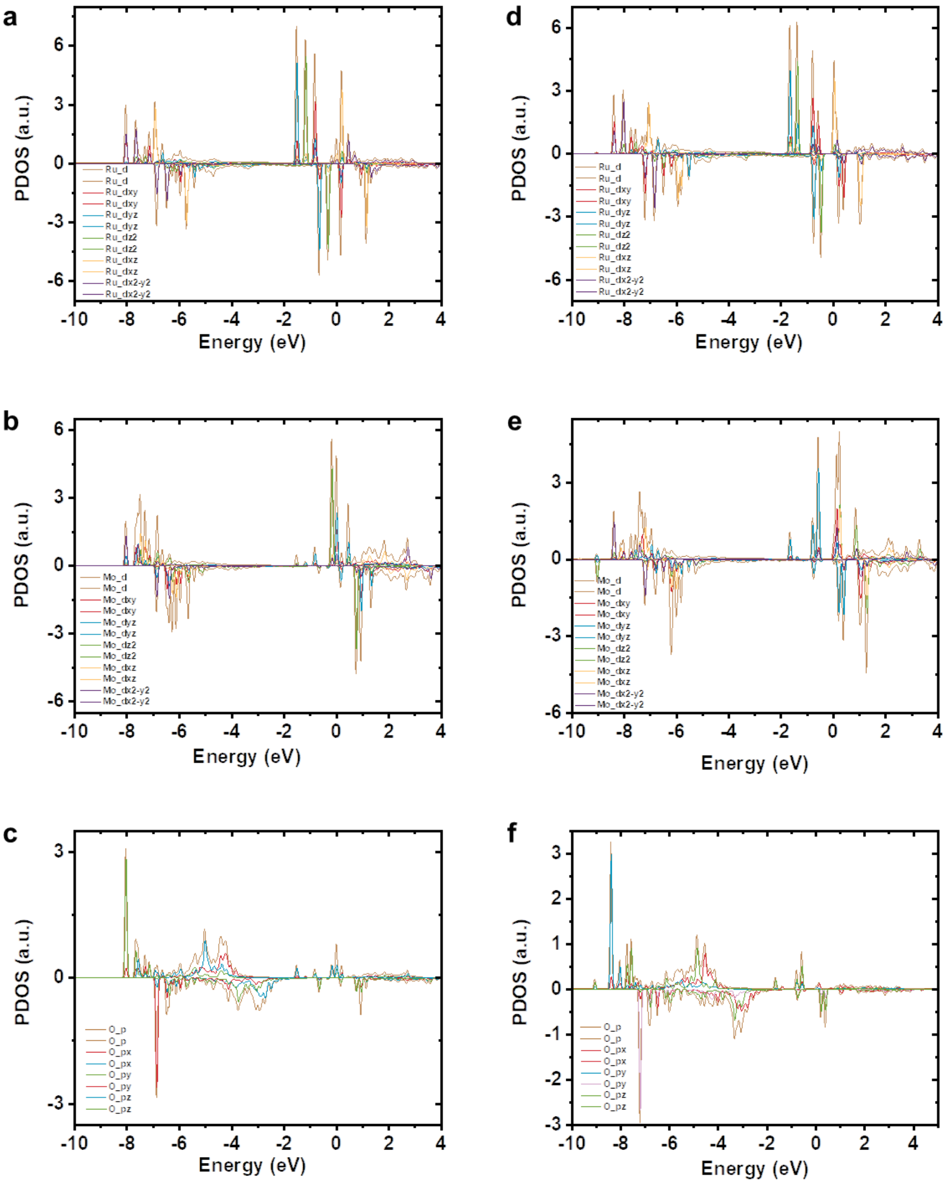

Supplementary Fig. 39 PDOS of (a) Ru *4d*, (b) Mo *4d* and (c) O *2p* of the Ru-O-Mo site, (d) Ru *4d*, (e) Mo *4d* and (f) O *2p* of the Ru-O-Mo site after LA adsorption.

As further revealed in the density of states (DOS) profiles, the *d*-band of Ru atom overlaps with the d-band of Mo atoms and the *p*-band of O atoms in $Ru4Mo_1/TiO_2$, suggesting obvious hybridization of Ru *4d* with Mo 4d and O *2p* orbitals. These demonstrate that the synergistic modifications of Ru and O atoms onto the Mo site could effectively enhance the interaction between the reactants and Mo active site and thus favor the adsorption of LA and its further conversion. For the Mo site, the distribution of *4d* orbitals after the adsorption of LA is the same as it of Mo SA/TiO_2, indicating that Mo SA adsorbs LA well. While for the Ru SA site, the distribution of *4d* orbitals before and after the adsorption of LA remains unchanged, indicating that Ru SA hardly adsorbs LA, which is consistent with the calculation results. However, the Ru d*xy* orbital of Ru-O-Mo varies significantly between -2 and 1 eV after LA adsorption, indicating that Ru is also influenced by LA adsorption at the Mo site.

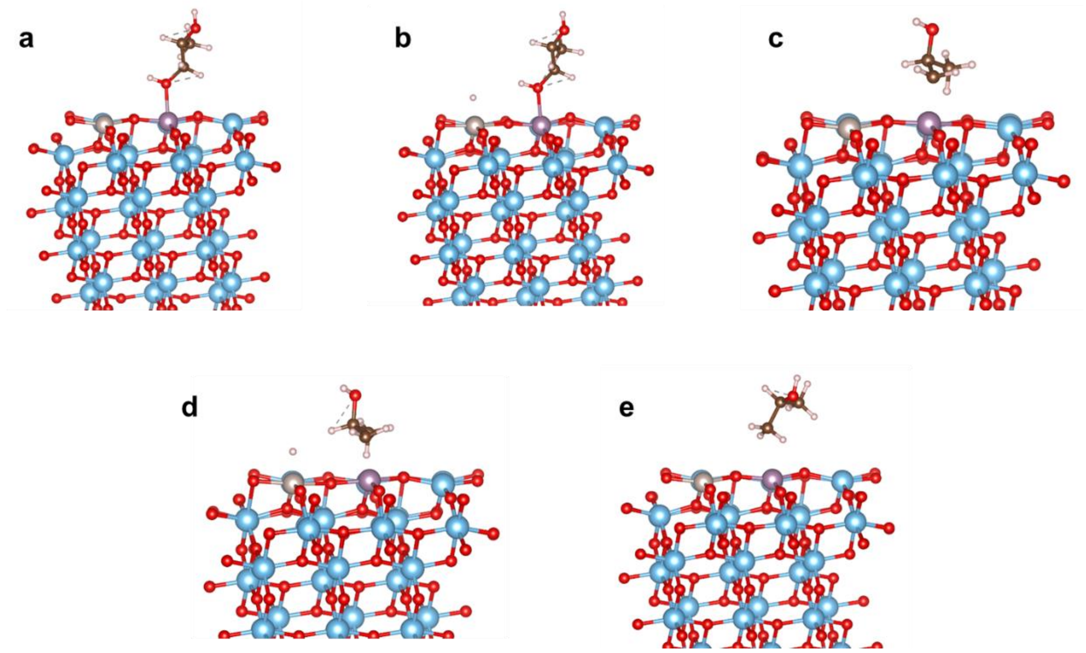

Supplementary Fig. 40 DFT calculation-optimized adsorption configurations of 1,2-propanediol and hydrogenated species on the Ru_4Mo_1/TiO_2. Gray: Ru, purple: Mo, red: O, blue: Ti and pink: H.

The adsorption energies of these three compounds on the catalyst surface are -1.68 eV (a), -2.16 eV (c), and -0.54 eV (e), respectively. Figures b and d represent the adsorption of one proton onto the catalysts shown in a and c, respectively. The adsorption energies of H_2 on Ru, Mo and Ti were estimated to be -0.31, -0.21 and -0.30 eV, and the dissociation energies of H_2 on Ru, Mo and Ti were estimated to be 0.56, 2.51 and 3.74 eV, respectively. The hydrodeoxygenation reaction of 1,2-propanediol over Ru_4Mo_1/TiO_2 was calculated, and the surface Mo sites act as the active center in favor of -OH activation. For the route $C_3H_6\text{-}2OH \xrightarrow{H^*} C_3H_6\text{-}2OH^*+H^* \rightarrow C_3H_6\text{-}OH^*+H_2O \xrightarrow[-H_2O]{H^*} C_3H_6\text{-}OH^*+H^* \rightarrow C_3H_7\text{-}OH$, the reaction energy barrier of each step over Ru_4Mo_1/TiO_2 is 0.33, 0.66, 0.16, -0.54 eV, respectively.

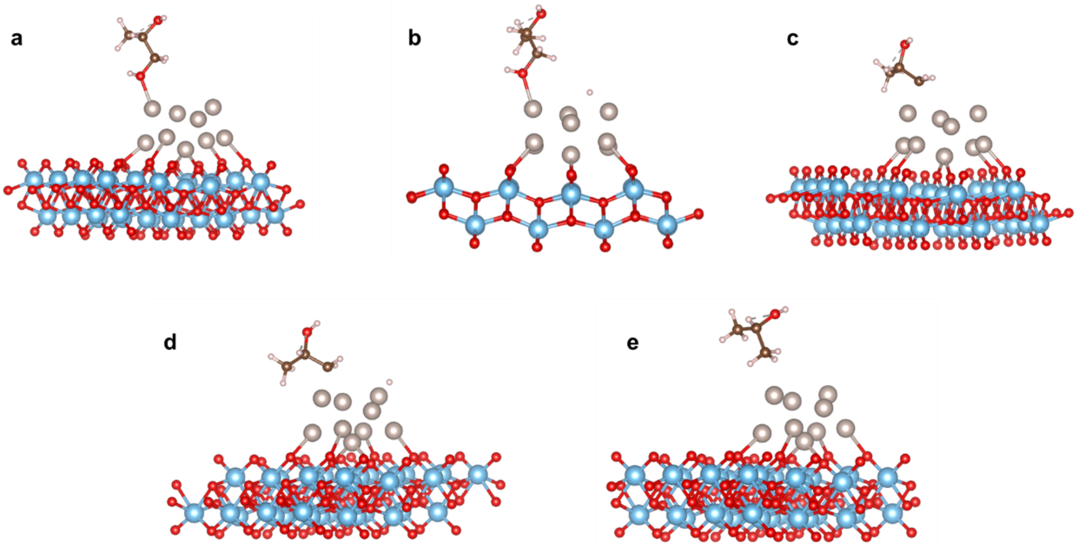

Supplementary Fig. 41 DFT calculation-optimized adsorption configurations of 1,2-propanediol and the hydrogenated species on Ru_6/TiO_2. (a) C_3H_6-2OH, (b) C_3H_6-2OH*+H*, (c) C_3H_6-OH*, (d) C_3H_6-OH*+H*, (e) C_3H_7-OH.

The adsorption energies of these three compounds on the catalyst surface are -1.20 eV (a), -2.65 eV (c), and -0.68 eV (e), respectively. The hydrodeoxygenation reaction of 1,2-propanediol over Ru_6/TiO_2 was calculated, and the surface metal sites act as the active center in favor of -OH activation adsorption. For the route $C_3H_6\text{-2OH} \xrightarrow{H^*} C_3H_6\text{-2OH}^*\text{+H}^* \rightarrow C_3H_6\text{-OH}^*\text{+}H_2O \xrightarrow[-H_2O]{H^*} C_3H_6\text{-OH}^*\text{+H}^* \rightarrow C_3H_7\text{-OH}$, the reaction energy barrier of each step over Ru_6/TiO_2 -0.27, 0.31, -0.16, 0.12 eV. The successive hydrodeoxygenation of 1,2-propanediol molecule (via hydroxy, methylene and methine groups) is the optimal path on both catalysts, which is consistent with the experimental results. Compared with Ru_6/TiO_2, Ru_4Mo_1/TiO_2 displays largely increased reaction energy barriers for 1,2-propanediol hydrogenation, demonstrating the advantages of dual single atom synergistic effect towards the single selectivity for LA hydrogenation to 1,2-propanediol.

Supplementary Table 1 The metal contents of different catalysts detected by ICP.

Catalysts	$RuCl_3·3H_2O$	$H_{24}Mo_7N_6O_{24}·4H_2O$	Element	Content wt(%)
Ru_6/TiO_2	5%	-	Ru<br>Mo	6.15%<br>-
Ru_4Mo_1/TiO_2	5%	1%	Ru<br>Mo	3.76%<br>0.96%
Ru_3Mo_{10}/TiO_2	5%	10%	Ru<br>Mo	3.22%<br>9.85%
Ru_1Mo_6/TiO_2	1%	10%	Ru<br>Mo	0.78%<br>6.13%
Ru_2Mo_1/TiO_2	2%	1%	Ru<br>Mo	1.96%<br>1.22%
$Ru_4Mo_{0.1}/TiO_2$	5%	0.1%	Ru<br>Mo	3.59%<br>0.15%
Mo_2/TiO_2	-	5%	Ru<br>Mo	-<br>1.74%

Supplementary Table 2 Structural parameters of the samples extracted from the Ru and Mo K-edge EXAFS fitting.

Samples	Shell	CN	R (Å)	σ^2 (10-3Å2)	ΔE_0(eV)	*R*-factor	Chi-square
Ru_4Mo_1/TiO_2 (Ru)	Ru-O	4.4	1.98	0.6	-4.3	0.019	118.06
Ru_4Mo_1/TiO_2 (Mo)	Mo-O	4.1	1.75	7.6	-9.9	0.019	1.56
Ru_6/TiO_2	Ru-Ru	3.0	2,39	0.004	3.0	0.003	27.2
	Ru-O	4.0	1.98	0.006	24.9	0.003	27.2
Mo_2/TiO_2	Mo-O	3.9	1.73	0.01	-9.8	0.018	147.2

CN: coordination numbers; R: bond distance; σ^2: Debye-Waller factors; ΔE_0: the inner potential correction; *R*-factor: goodness of fit.

The obtained XAFS data was processed in Athena (version 0.9.26) for background, pre-edge line and post-edge line calibrations. Then Fourier transformed fitting was carried out in Artemis (version 0.9.26). The variable parameters, coordination number, bond length, Debye-Waller factor and E0 shift (CN, R, ΔE_0, σ^2) were fitted with IFEFFIT program. S_0^2 was fixed with the Ru-Ru path in Ru foil or Mo-Mo path in Mo foil. Since the data of different samples were obtained from different tests, each test had different S_0^2. S_0^2 was fixed to 0.678 as determined from Ru foil fitting. $S_0^2$was fixed to 0.808 as determined from Mo foil fitting. The k^2 weighting, k-range of 2.9~8.2 Å^{-1} and R range of 1.0~2.6 Å were used for the fitting of Ru in Ru-O-Mo; the k^2 weighting, *k*-range of 2.9~9.8 Å^{-1} and *R* range of 1.0~2.5 Å were used for the fitting of Mo in Ru-O-Mo; the k^2 weighting, *k*-range of 3.0~10.0 Å^{-1}and *R* range of 1.0~3.0 Å were used for the fitting of Ru-NP; the k^2 weighting, *k*-range of 3.0~10.0 Å^{-1} and *R* range of 0.7~2.0 Å were used for the fitting of Mo-SA.

^{1}H and ^{13}C NMR spectra of the products

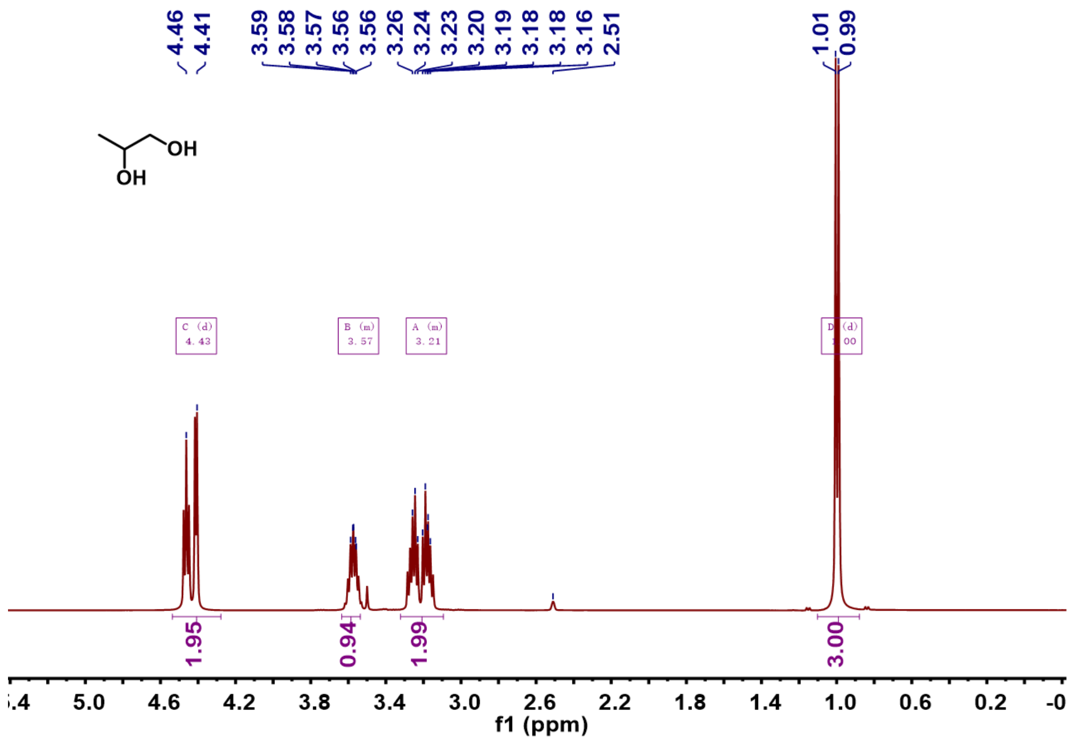

^{1}H NMR (400 MHz, DMSO-d_6) δ 4.43 (d, J = 23.0 Hz, 2H), 3.64 – 3.54 (m, 1H), 3.32 – 3.09 (m, 2H), 1.00 (d, J = 6.3 Hz, 3H).

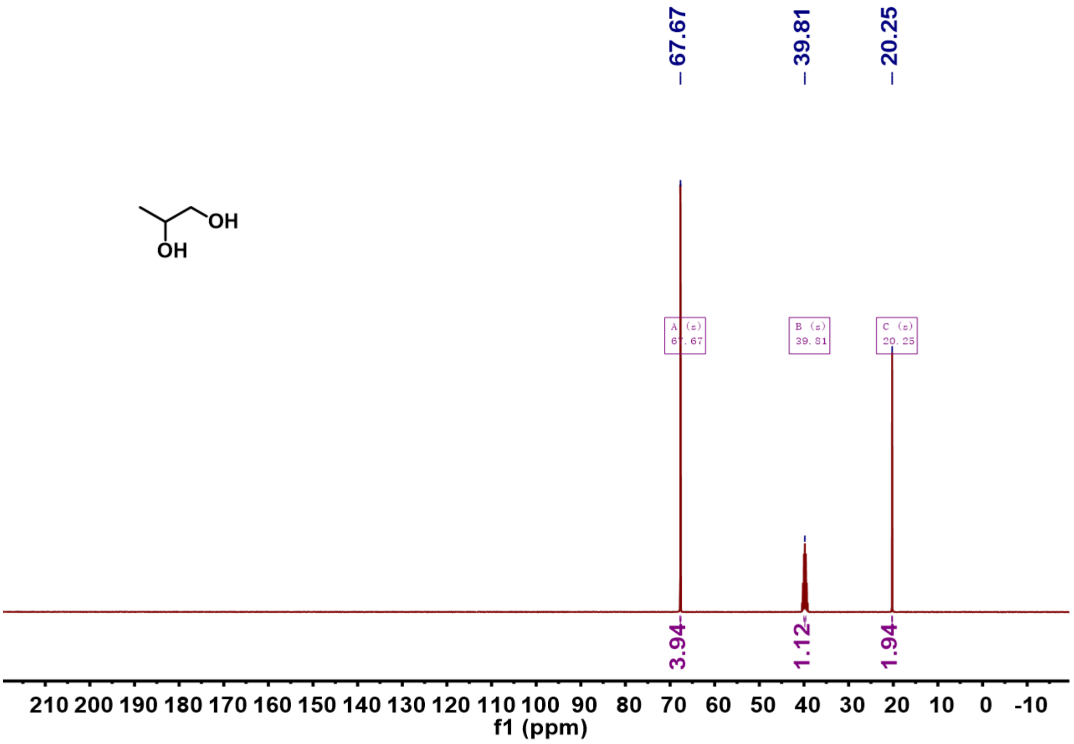

^{13}C NMR (101 MHz, DMSO-d_6) δ 67.67, 39.81, 20.25.

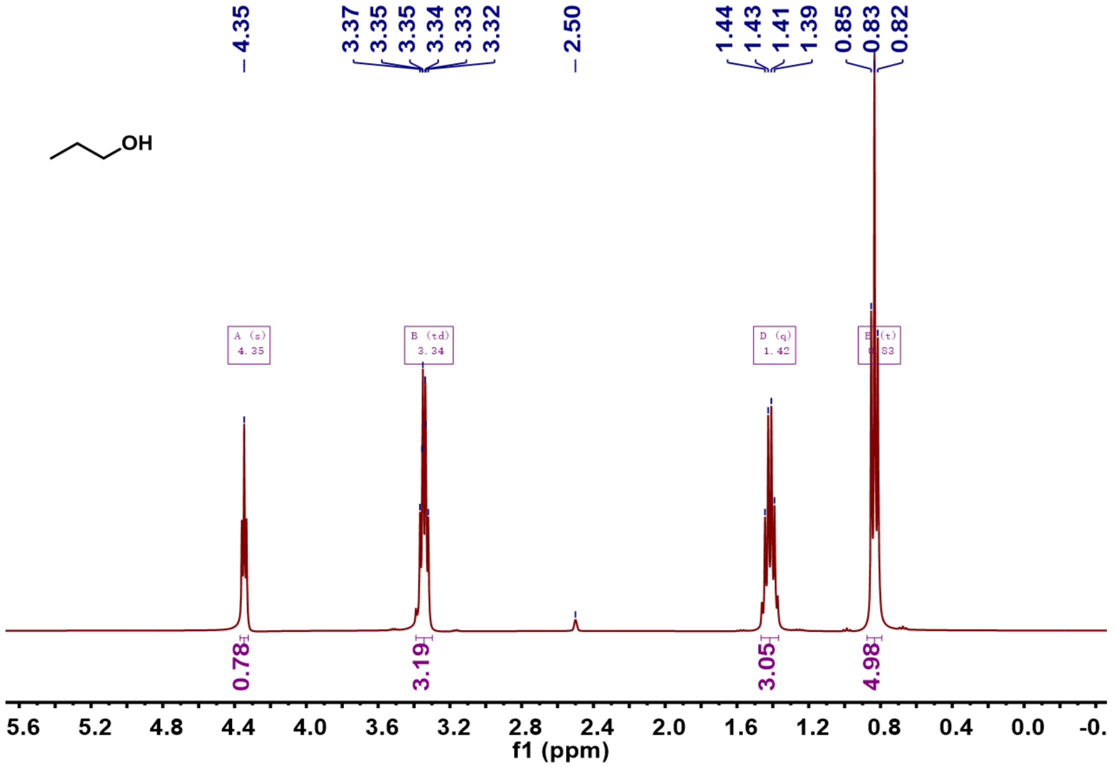

^{1}H NMR (400 MHz, DMSO-d_6) δ 4.35 (s, 1H), 3.34 (td, J = 6.6, 5.1 Hz, 3H), 1.42 (q, J = 7.1 Hz, 3H), 0.83 (t, J = 7.4 Hz, 5H).

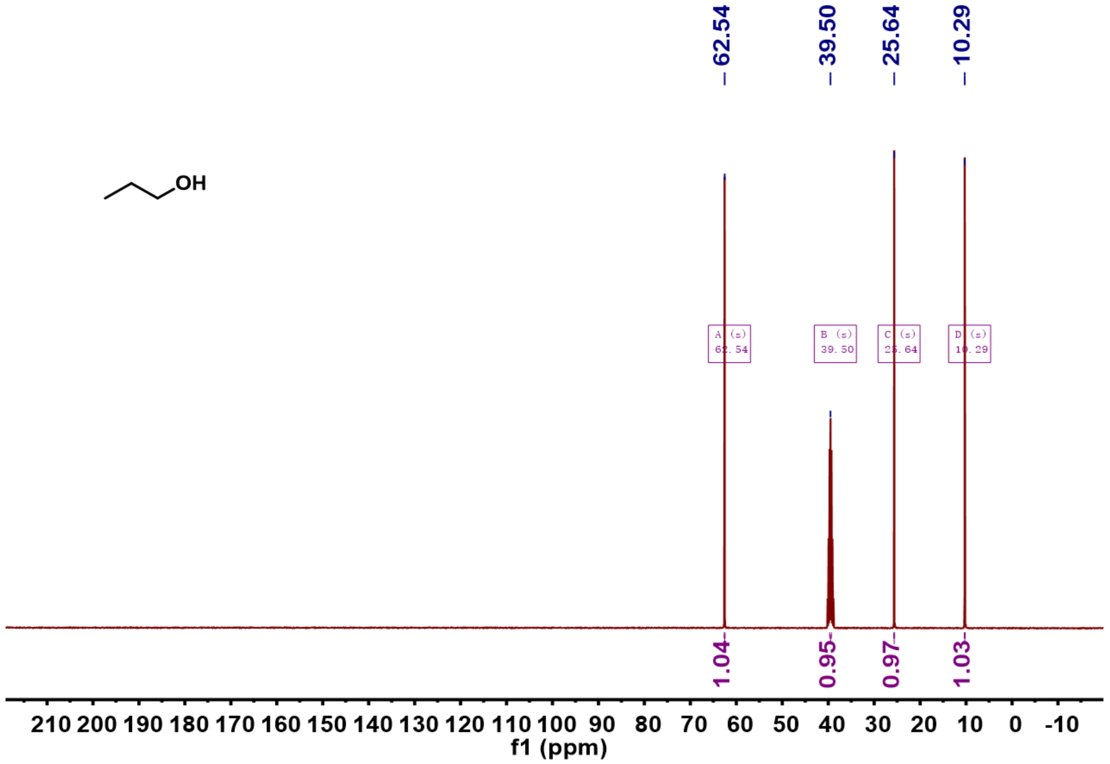

^{13}C NMR (101 MHz, DMSO-d_6) δ 62.54, 39.50, 25.64, 10.29.

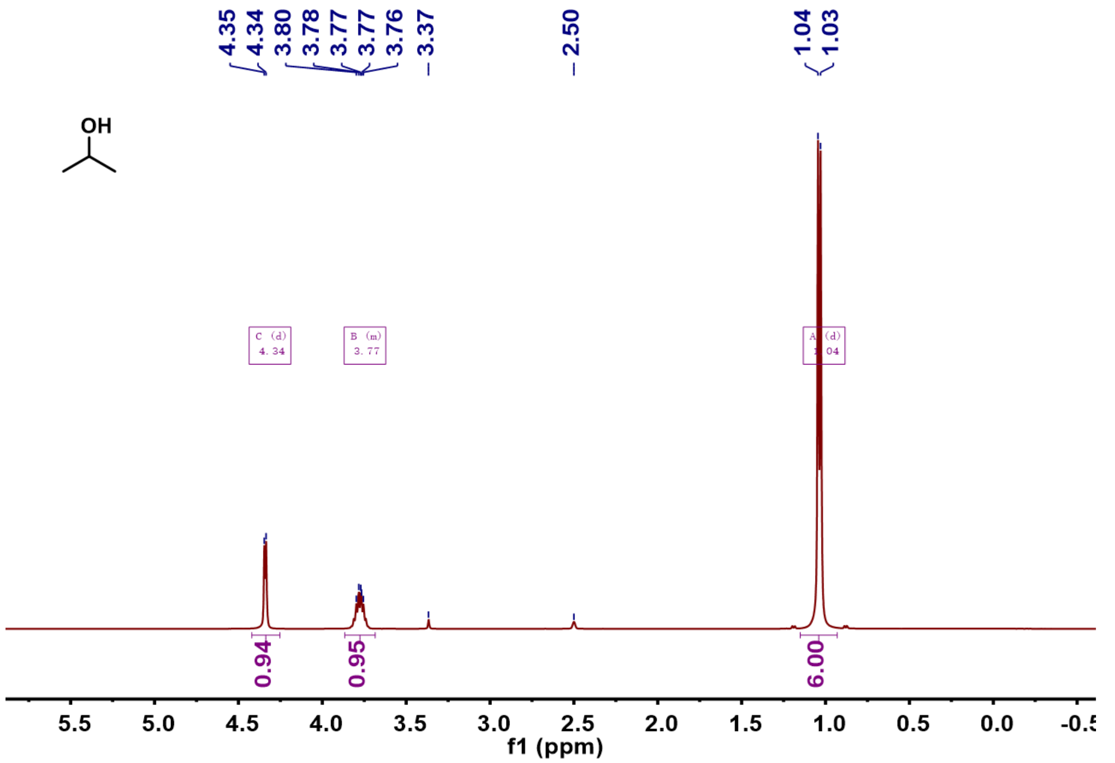

^{1}H NMR (400 MHz, DMSO-d_6) δ 4.34 (d, J = 4.2 Hz, 1H), 3.87 – 3.68 (m, 1H), 1.04 (d, J = 6.2 Hz, 6H).

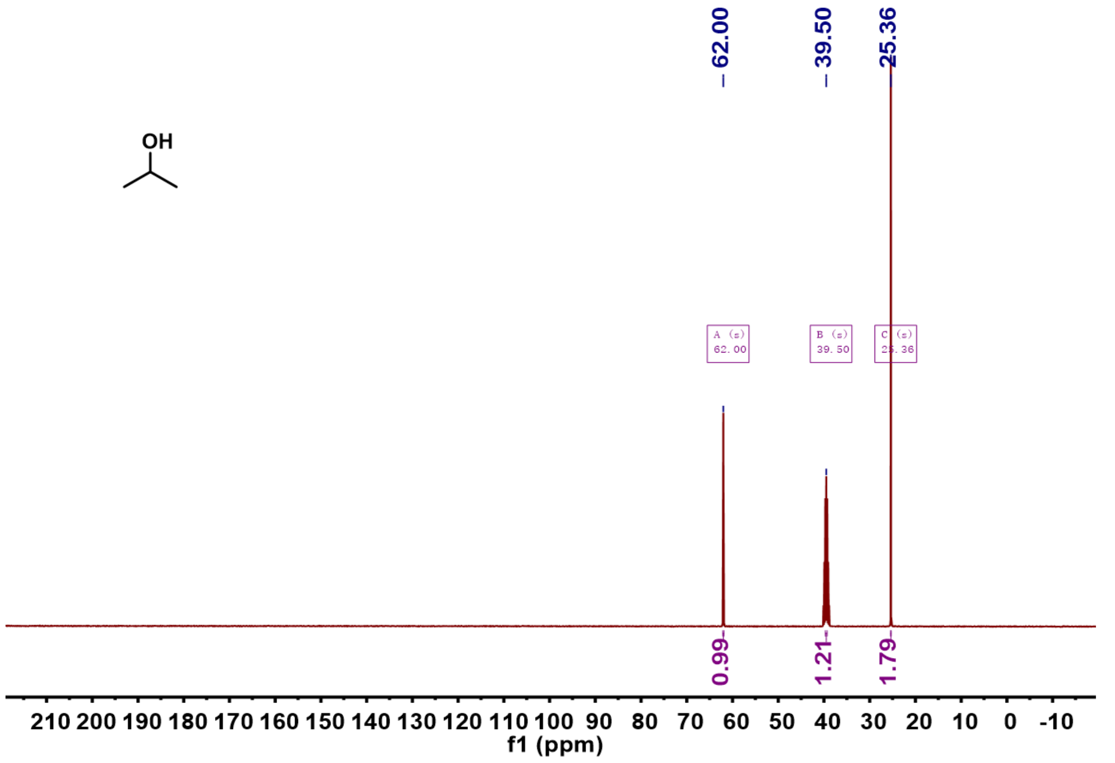

^{13}C NMR (101 MHz, DMSO-d_6) δ 62.00, 39.50, 25.36.

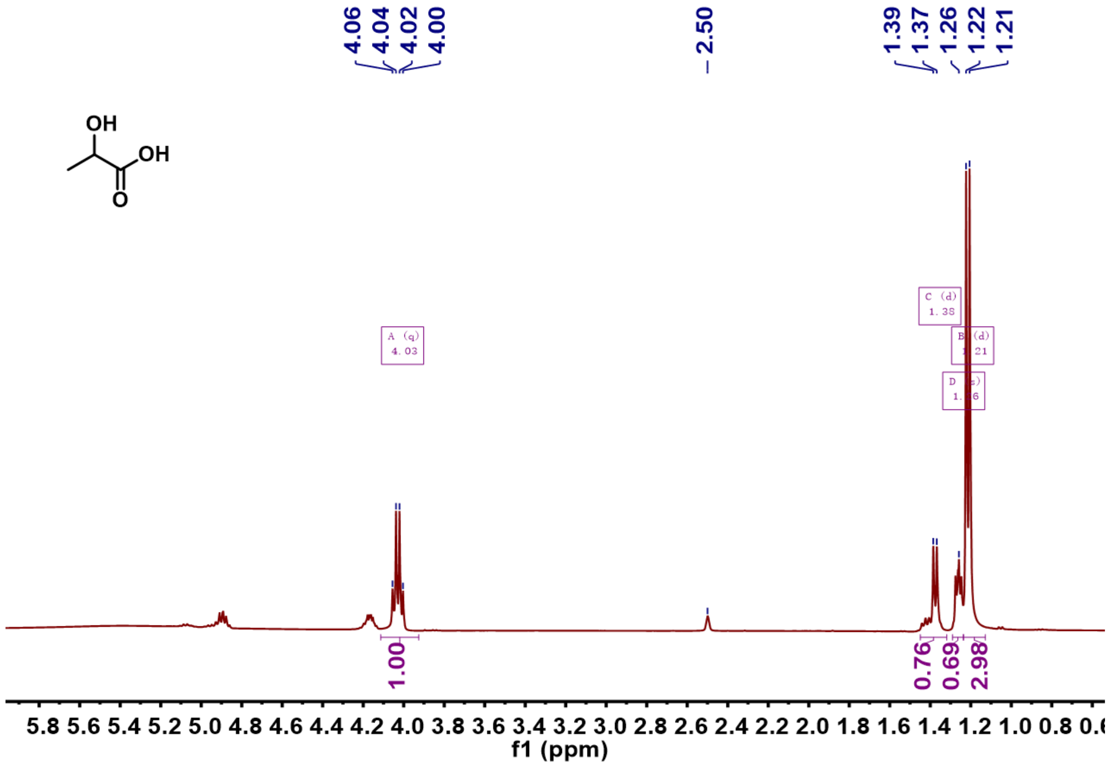

^{1}H NMR (400 MHz, DMSO-d_6) δ 4.03 (q, J = 6.9 Hz, 1H), 1.38 (d, J = 7.1 Hz, 1H), 1.26 (s, 1H), 1.21 (d, J = 6.9 Hz, 3H).

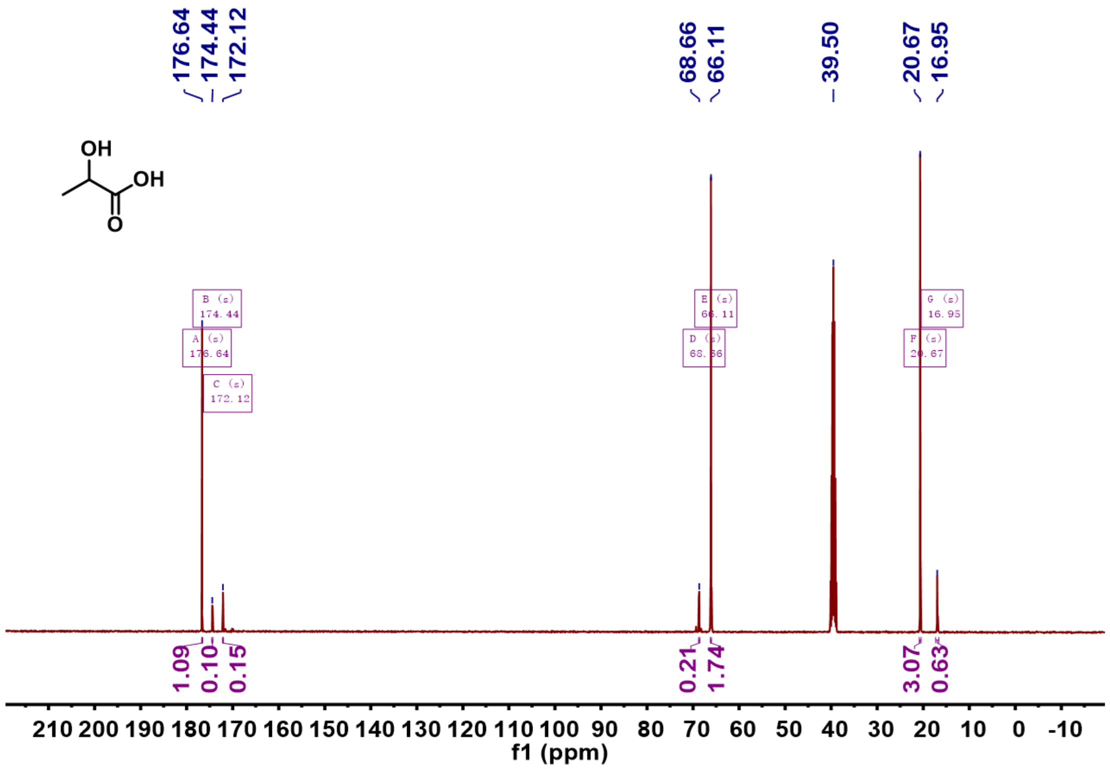

^{13}C NMR (101 MHz, DMSO-d_6) δ 176.64, 174.44, 172.12, 68.66, 66.11, 20.67, 16.95.

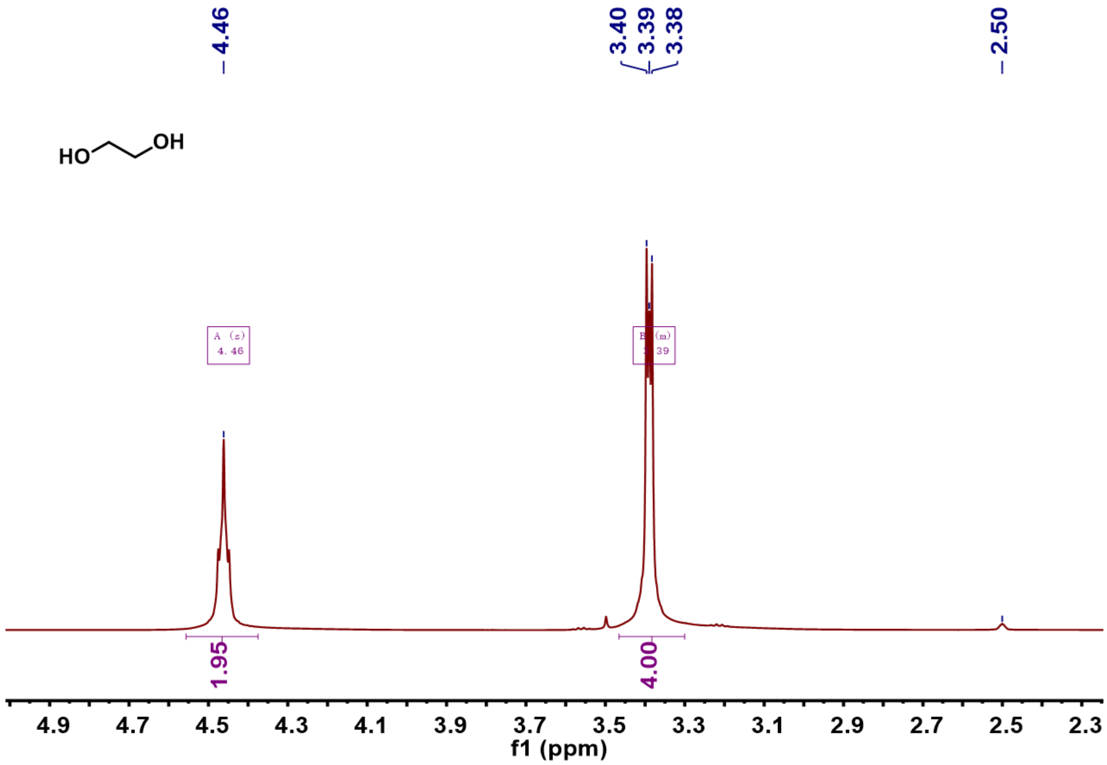

^{1}H NMR (400 MHz, DMSO-d_6) δ 4.46 (s, 2H), 3.47 – 3.30 (m, 4H).

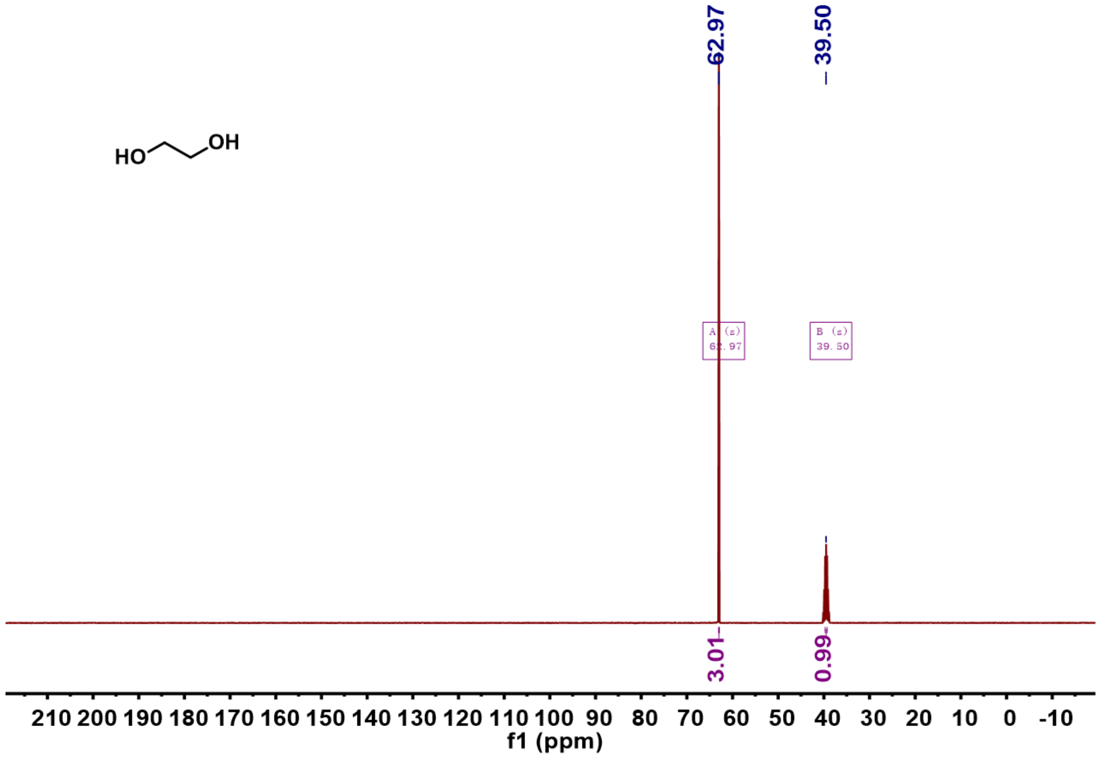

^{13}C NMR (101 MHz, DMSO-d_6) δ 62.97, 39.50.

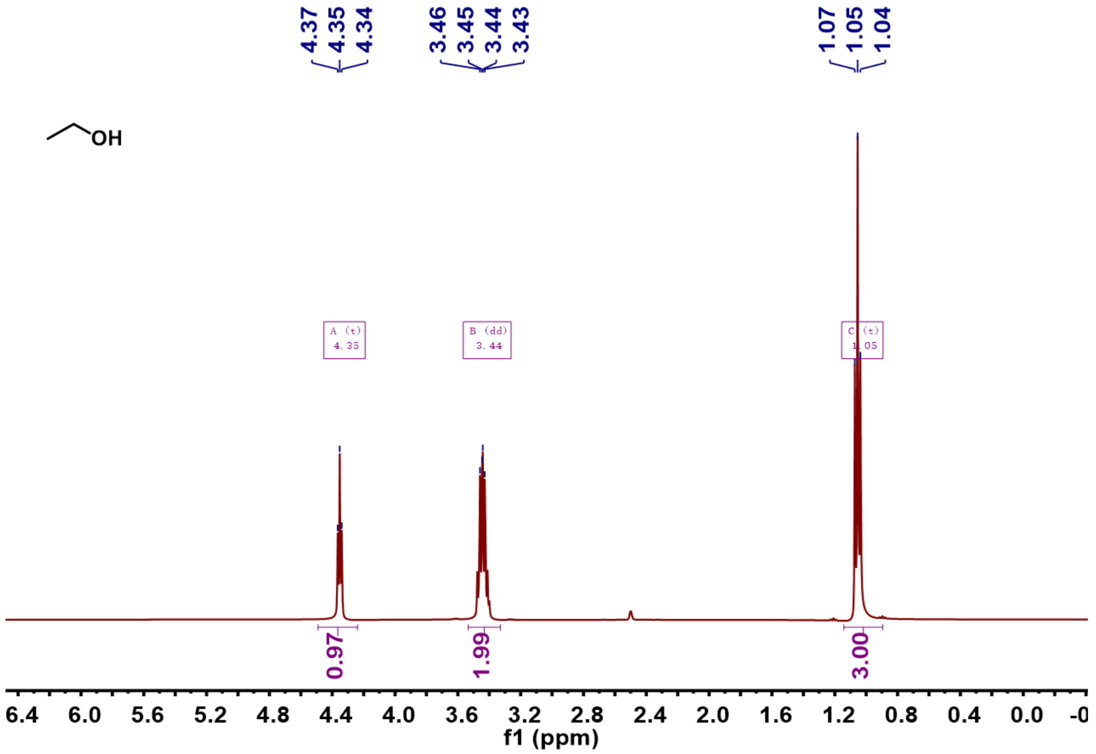

^{1}H NMR (400 MHz, DMSO-d_6) δ 4.35 (t, J = 5.1 Hz, 1H), 3.44 (dd, J = 7.0, 5.1 Hz, 2H), 1.05 (t, J = 7.0 Hz, 3H).

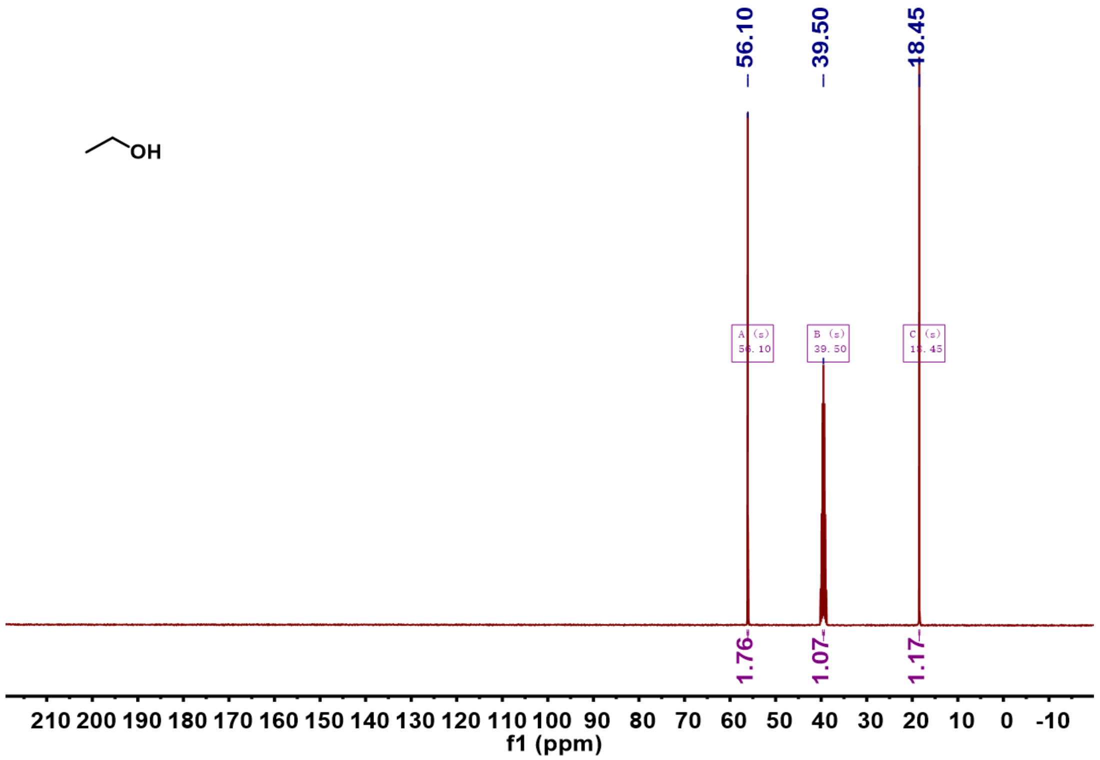

^{13}C NMR (101 MHz, DMSO-d_6) δ 56.10, 39.50, 18.45.

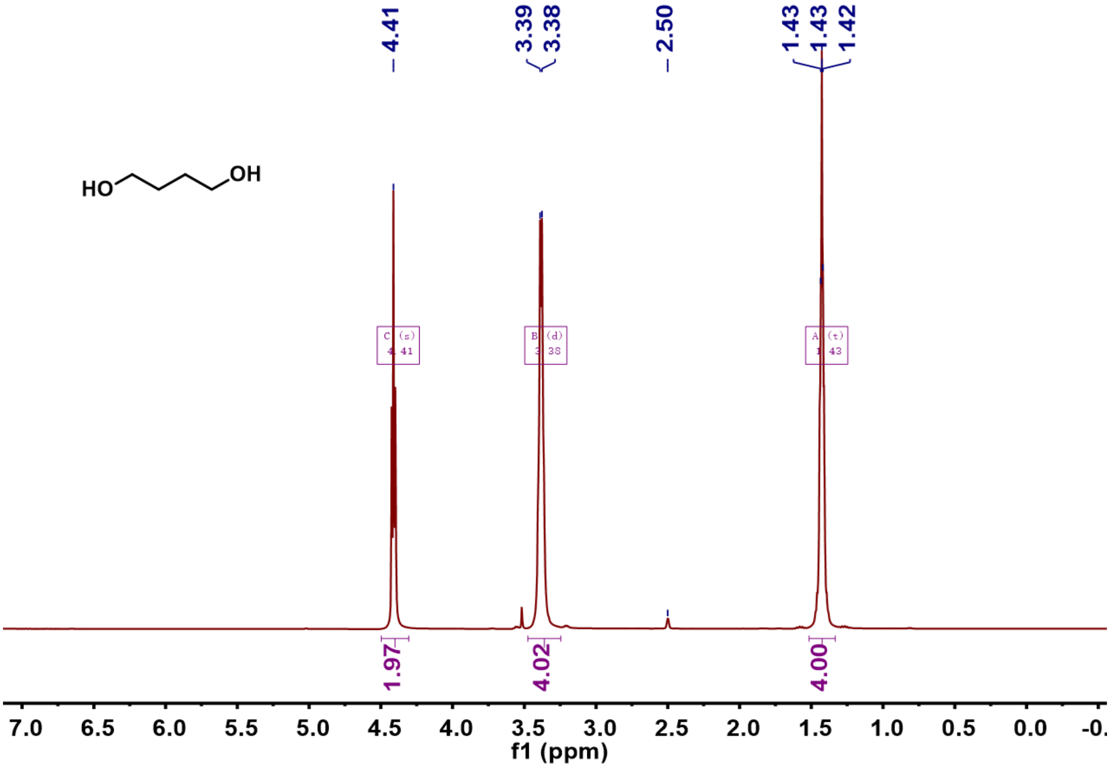

^{1}H NMR (400 MHz, DMSO-d_6) δ 4.41 (s, 2H), 3.38 (d, J = 5.2 Hz, 4H), 1.43 (t, J = 3.0 Hz, 4H).

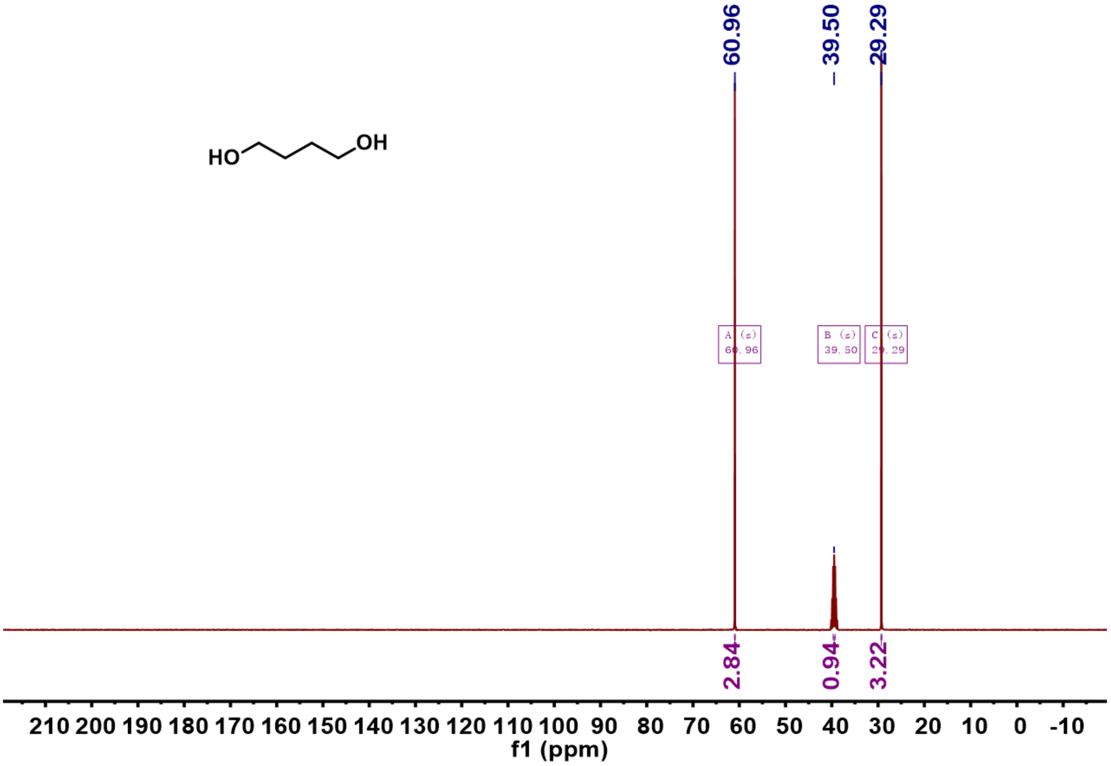

^{13}C NMR (101 MHz, DMSO-d_6) δ 60.96, 39.50, 29.29.

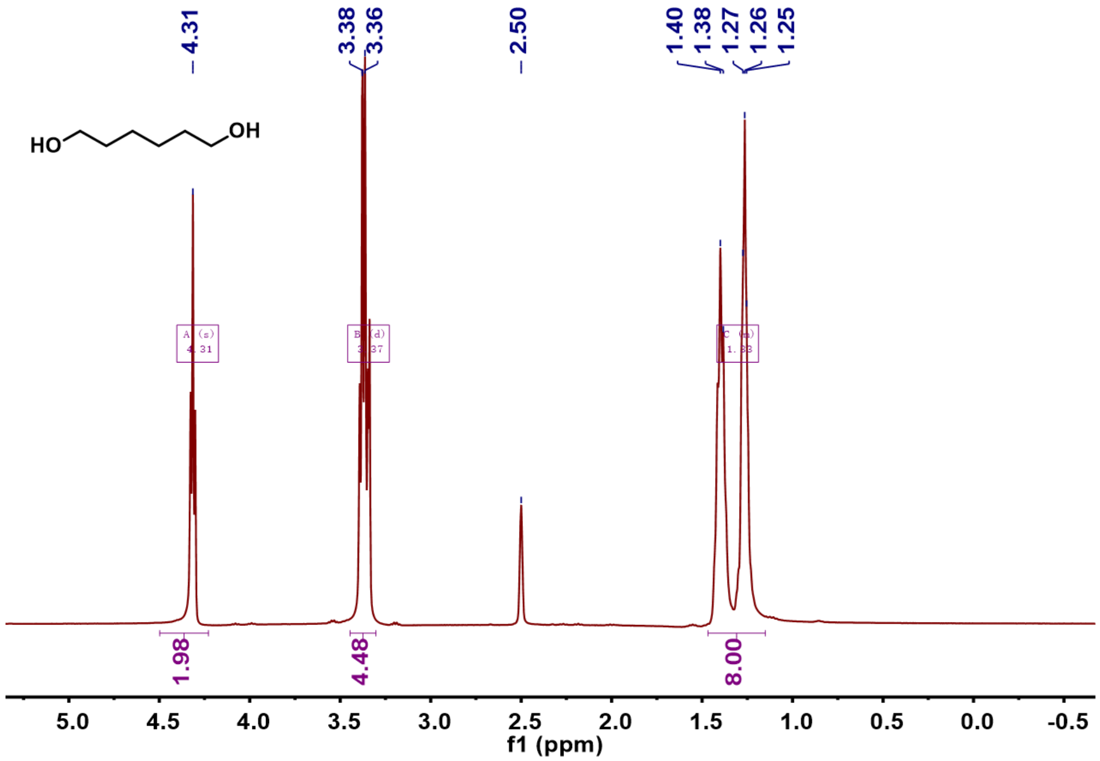

^{1}H NMR (400 MHz, DMSO-d_6) δ 4.31 (s, 2H), 3.37 (d, J = 5.9 Hz, 4H), 1.47 – 1.15 (m, 8H).

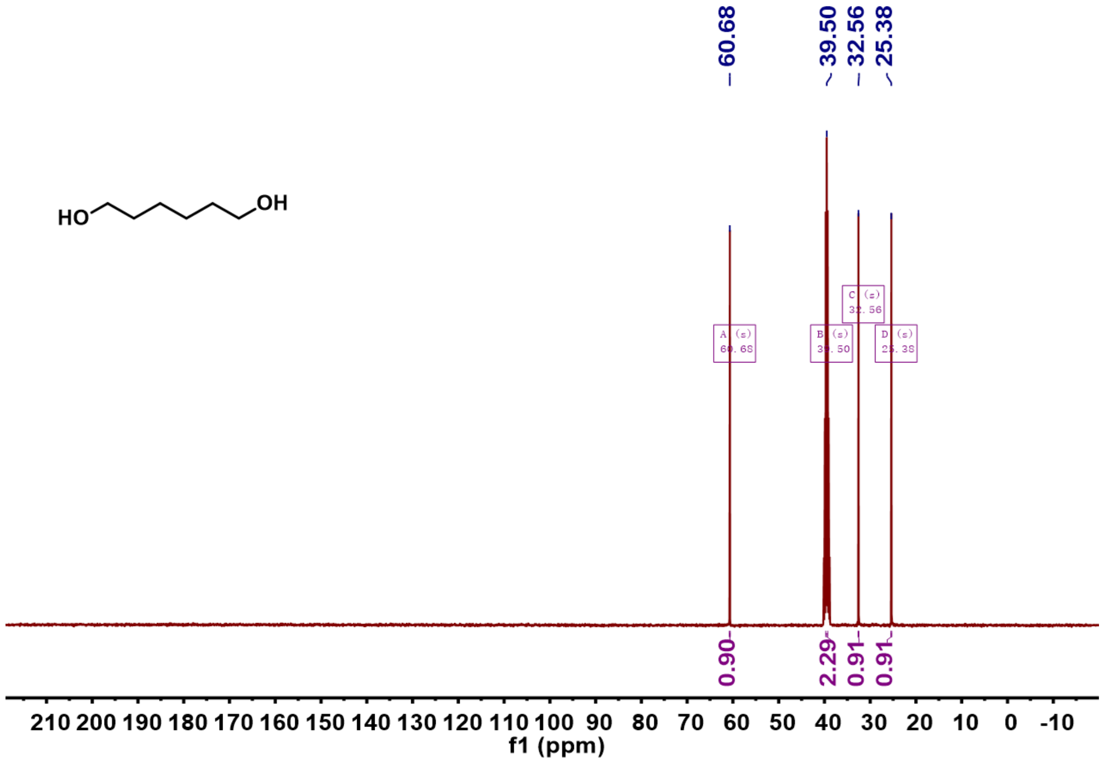

^{13}C NMR (101 MHz, DMSO-d_6) δ 60.68, 39.50, 32.56, 25.38.
