# Supplementary material for: Highly efficient recycling of polyester wastes to diols using Ru and Mo dual-atom catalyst
Source: Nat Commun. 2024 Jul 4;15:5630. doi: 10.1038/s41467-024-49880-z (PMC11224329; doi:10.1038/s41467-024-49880-z)
Supplement: Supplementary file 3 — Description of Additional Supplementary Files [file 41467_2024_49880_MOESM3_ESM.pdf]

Description of Additional Supplementary Files

Supplementary Data 1: The calculated atomic xyz coordinates of each optimized structure including the involved catalysts, reactants, products, and intermediates. Data in each line represents for the atomic coordinates of one atom.
