# Supplementary material for: Highly efficient recycling of polyester wastes to diols using Ru and Mo dual-atom catalyst
Source: Nat Commun. 2024 Jul 4;15:5630. doi: 10.1038/s41467-024-49880-z (PMC11224329; doi:10.1038/s41467-024-49880-z)
Supplement: Supplementary file 4 — Supplementary Data 1 [file 41467_2024_49880_MOESM4_ESM.pdf]

DFT Details

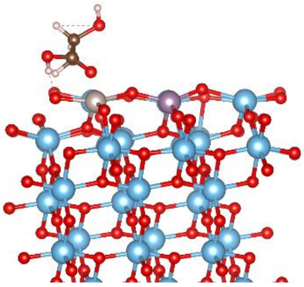

Supplementary Fig. 35a

Ti O Mo Ru C H

52 123 1 1 3 6

0.2162500020000024	0.0720800010000033	0.0971200019999969
0.4121899900000017	0.1374000010000032	0.2082500010000032
0.1075700000000026	0.2017599939999997	0.3191800119999968
0.3223899899999978	0.2694700059999988	0.4248200059999974
0.1132100000000023	0.2044000030000035	0.1804700049999965
0.3091399970000026	0.2697100039999967	0.2915900050000033
0.4172700049999989	0.1390900020000032	0.0693399979999967
0.0111699999999999	0.0034199999999984	0.4150600139999980
0.2061800059999968	0.0639299970000025	0.5169799920000031
0.7162500020000024	0.0720800010000033	0.0971200019999969
0.9121900199999970	0.1374000010000032	0.2082500010000032
0.6080600020000020	0.2023700029999986	0.3199799949999971
0.8129900100000000	0.2696099879999991	0.4331600069999979
0.6132100219999970	0.2044000030000035	0.1804700049999965
0.8091400269999980	0.2697100039999967	0.2915900050000033
0.9172700049999989	0.1390900020000032	0.0693399979999967
0.5113999840000005	0.0040400000000034	0.4151499869999995
0.7064099910000010	0.0657000019999998	0.5152699949999970
0.2162500020000024	0.4054200050000034	0.0971200019999969
0.4121899900000017	0.4707300070000002	0.2082500010000032
0.1083699989999971	0.5364099740000015	0.3192299899999966
0.3214699919999973	0.6141999959999964	0.4265199900000027
0.1132100000000023	0.5377399919999988	0.1804700049999965
0.3091399970000026	0.6030499940000027	0.2915900050000033
0.4172700049999989	0.4724200069999966	0.0693399979999967
0.0018999999999991	0.3338199849999981	0.4078600109999968
0.7162500020000024	0.4054200050000034	0.0971200019999969
0.9121900199999970	0.4707300070000002	0.2082500010000032
0.6079300050000001	0.5363500119999998	0.3196099999999973
0.8137900230000028	0.6065099839999988	0.4318799970000029
0.6132100219999970	0.5377399919999988	0.1804700049999965
0.8091400269999980	0.6030499940000027	0.2915900050000033
0.9172700049999989	0.4724200069999966	0.0693399979999967

0.5153499839999967	0.3389999870000011	0.4159300029999997
0.7077400089999983	0.4033299979999967	0.5147299770000018
0.2162500020000024	0.7387499809999980	0.0971200019999969
0.4121899900000017	0.8040599819999983	0.2082500010000032
0.1092500020000031	0.8700299859999987	0.3198699949999977
0.3179000020000018	0.9390599729999991	0.4311099949999999
0.1132100000000023	0.8710700270000018	0.1804700049999965
0.3091399970000026	0.9363800289999986	0.2915900050000033
0.4172700049999989	0.8057600260000015	0.0693399979999967
0.0036199999999980	0.6676599979999978	0.4101600050000016
0.7162500020000024	0.7387499809999980	0.0971200019999969
0.9121900199999970	0.8040599819999983	0.2082500010000032
0.6075699929999985	0.8695999979999982	0.3198499980000022
0.8191800119999968	0.9394699930000030	0.4279299969999997
0.6132100219999970	0.8710700270000018	0.1804700049999965
0.8091400269999980	0.9363800289999986	0.2915900050000033
0.9172700049999989	0.8057600260000015	0.0693399979999967
0.5146399739999978	0.6710900069999965	0.4159600139999995
0.7072200180000010	0.7384600039999967	0.5159699920000023
0.1305200010000007	0.0435100010000014	0.1664600069999977
0.3264499900000004	0.1088199989999978	0.2775900070000006
0.4345799979999967	0.3115299939999971	0.0553399989999974
0.0293400010000013	0.1772899930000023	0.3975299889999988
0.2262199970000012	0.2542400060000034	0.5435600280000017
0.0274700000000010	0.1758199929999975	0.2498099949999997
0.2267699989999983	0.2455500069999985	0.3635900020000022
0.4213500019999969	0.3162199849999965	0.4740999939999995
0.1356000010000002	0.0452000009999978	0.0275599989999975
0.3315300049999976	0.1105099990000014	0.1386799960000005
0.0905399990000006	0.0298299990000004	0.3367600139999993
0.2873699960000025	0.0996899980000023	0.4522100089999981
0.0030000000000001	0.1676699969999973	0.0000000000000000
0.1989399939999998	0.2329799979999976	0.1111199999999997
0.3948799969999968	0.2982900140000027	0.2222500000000025
0.1938599940000003	0.2312899980000012	0.2500300109999998
0.3991700110000025	0.2940100129999976	0.3600299949999979
0.3019900019999966	0.1006600039999981	0.0277799999999999
0.4979299900000029	0.1659799959999972	0.1388999970000029
0.1032600030000026	0.0440200009999998	0.4816800059999977
0.6305199860000030	0.0435100010000014	0.1664600069999977
0.8264499900000004	0.1088199989999978	0.2775900070000006
0.9345800279999992	0.3115299939999971	0.0553399989999974
0.5301700229999966	0.1771599949999967	0.3982200029999987
0.7334100010000029	0.2441799939999996	0.5260000230000017

0.5274699929999969	0.1758199929999975	0.2498099949999997
0.7273899910000026	0.2405399980000027	0.3637399970000033
0.9212499859999994	0.3076300020000033	0.4740799960000004
0.6355999709999978	0.0452000009999978	0.0275599989999975
0.8315299750000023	0.1105099990000014	0.1386799960000005
0.5897600050000023	0.0300099999999972	0.3368000090000010
0.7858499880000025	0.0922100020000016	0.4492900070000019
0.5030000209999983	0.1676699969999973	0.0000000000000000
0.6989399790000022	0.2329799979999976	0.1111199999999997
0.8948799969999968	0.2982900140000027	0.2222500000000025
0.6938599940000003	0.2312899980000012	0.2500300109999998
0.8935300110000028	0.2989200060000030	0.3628000020000002
0.8019899729999977	0.1006600039999981	0.0277799999999999
0.9979299900000029	0.1659799959999972	0.1388999970000029
0.6015099879999966	0.0313599999999994	0.4803699850000029
0.1305200010000007	0.3768399949999974	0.1664600069999977
0.3264499900000004	0.4421499970000013	0.2775900070000006
0.4345799979999967	0.6448600290000002	0.0553399989999974
0.0327900010000022	0.5111200209999964	0.4001100059999985
0.2221799940000011	0.5642099980000026	0.5350300070000031
0.0274700000000010	0.5091599819999999	0.2498099949999997
0.2267799970000013	0.5746200079999966	0.3642700019999978
0.4218499959999988	0.6262800100000021	0.4741500020000018
0.1356000010000002	0.3785299959999975	0.0275599989999975
0.3315300049999976	0.4438399969999978	0.1386799960000005
0.0892499979999997	0.3636200130000020	0.3357999919999983
0.2833000119999980	0.4265899959999970	0.4493899940000006
0.0030000000000001	0.5009999870000001	0.0000000000000000
0.1989399939999998	0.5663099880000004	0.1111199999999997
0.3948799969999968	0.6316300029999979	0.2222500000000025
0.1938599940000003	0.5646200179999994	0.2500300109999998
0.3977900149999982	0.6409800050000030	0.3610199989999998
0.3019900019999966	0.4339999849999998	0.0277799999999999
0.4979299900000029	0.4993099869999966	0.1388999970000029
0.0989599970000015	0.3597100080000004	0.4823699889999986
0.6305199860000030	0.3768399949999974	0.1664600069999977
0.8264499900000004	0.4421499970000013	0.2775900070000006
0.9345800279999992	0.6448600290000002	0.0553399989999974
0.5294799799999979	0.5098099710000028	0.3965699970000003
0.7324299810000028	0.5783500080000010	0.5280699730000009
0.5274699929999969	0.5091599819999999	0.2498099949999997
0.7275300029999983	0.5776100160000013	0.3634499910000031
0.9201700090000031	0.6401600239999965	0.4747500120000012
0.6355999709999978	0.3785299959999975	0.0275599989999975

0.8315299750000023	0.4438399969999978	0.1386799960000005
0.5921099780000034	0.3641299900000021	0.3374899920000018
0.7838699819999988	0.4287199970000017	0.4476999939999970
0.5030000209999983	0.5009999870000001	0.0000000000000000
0.6989399790000022	0.5663099880000004	0.1111199999999997
0.8948799969999968	0.6316300029999979	0.2222500000000025
0.6938599940000003	0.5646200179999994	0.2500300109999998
0.8951299790000036	0.6326900119999976	0.3632099929999981
0.8019899729999977	0.4339999849999998	0.0277799999999999
0.9979299900000029	0.4993099869999966	0.1388999970000029
0.6007800100000011	0.3676500019999978	0.4821299910000008
0.1305200010000007	0.7101699709999991	0.1664600069999977
0.3264499900000004	0.7754799719999994	0.2775900070000006
0.4345799979999967	0.9781900050000019	0.0553399989999974
0.0343700009999992	0.8433200119999995	0.4014999870000011
0.2219299969999966	0.9049199820000027	0.5234400029999975
0.0274700000000010	0.8424900170000029	0.2498099949999997
0.2264199999999974	0.9067500230000007	0.3641999960000035
0.4218600089999995	0.9805999989999989	0.4759800140000010
0.1356000010000002	0.7118700150000024	0.0275599989999975
0.3315300049999976	0.7771800160000026	0.1386799960000005
0.0891700009999994	0.6960999969999975	0.3365100030000008
0.2953099910000034	0.7634400130000003	0.4585100110000013
0.0030000000000001	0.8343300220000032	0.0000000000000000
0.1989399939999998	0.8996499779999994	0.1111199999999997
0.3948799969999968	0.9649599789999996	0.2222500000000025
0.1938599940000003	0.8979499940000011	0.2500300109999998
0.3947600130000026	0.9629499910000021	0.3637000020000016
0.3019900019999966	0.7673299909999969	0.0277799999999999
0.4979299900000029	0.8326399919999972	0.1388999970000029
0.0858400019999976	0.6878200170000000	0.4962199929999969
0.6305199860000030	0.7101699709999991	0.1664600069999977
0.8264499900000004	0.7754799719999994	0.2775900070000006
0.9345800279999992	0.9781900050000019	0.0553399989999974
0.5293200019999986	0.8432400229999999	0.3982400000000013
0.7281600239999975	0.9091899989999987	0.5350099799999981
0.5274699929999969	0.8424900170000029	0.2498099949999997
0.7275300029999983	0.9097899790000028	0.3632099929999981
0.9229699970000027	0.9737499949999986	0.4751099940000003
0.6355999709999978	0.7118700150000024	0.0275599989999975
0.8315299750000023	0.7771800160000026	0.1386799960000005
0.5910500290000016	0.6973900200000003	0.3371599910000000
0.7851799729999982	0.7644600269999984	0.4493600130000033
0.5030000209999983	0.8343300220000032	0.0000000000000000

0.6989399790000022	0.8996499779999994	0.1111199999999997
0.8948799969999968	0.9649599789999996	0.2222500000000025
0.6938599940000003	0.8979499940000011	0.2500300109999998
0.8982899789999976	0.9644500019999995	0.3621599969999991
0.8019899729999977	0.7673299909999969	0.0277799999999999
0.9979299900000029	0.8326399919999972	0.1388999970000029
0.6016399860000021	0.7026500110000029	0.4812200069999975
0.4290110443700103	0.7097785739108240	0.6858317021851628
0.3248428992782967	0.7470558303389873	0.5817136904282123
0.3454171576299243	0.9396131493960623	0.6166636017333106
0.2046100049999993	0.3986899849999972	0.5146800280000008
0.1960200070000013	0.7266899939999973	0.5159500239999986
0.5239399537627246	0.8269305741349554	0.6099905095798487
0.4457836104163303	0.8221483673489357	0.6537548666503352
0.3654940160265037	0.8316606235895969	0.6145321759575503
0.5852388947088561	0.8332109864859655	0.6368462266568474
0.5329936617592167	0.9050286310185705	0.5786500532656194
0.5092244662080482	0.7438162880469256	0.5819157680347270
0.4605804276064319	0.9006391474097601	0.6852695197944563
0.3990480959720450	0.7172329659430303	0.7244609330074734
0.2983705123053669	0.9341028317537220	0.5844052154920752

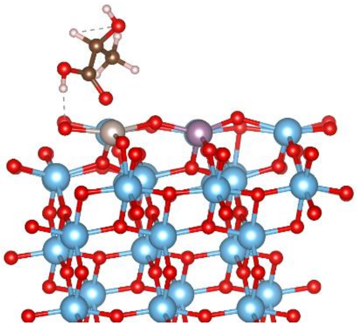

Supplementary Fig. 35b

Ti	O	Mo	Ru	C	H
52	123	1	1	3	6

0.2162500020000024	0.0720800010000033	0.0971200019999969
0.4121899900000017	0.1374000010000032	0.2082500010000032
0.1075700000000026	0.2017599939999997	0.3191800119999968
0.3223899899999978	0.2694700059999988	0.4248200059999974
0.1132100000000023	0.2044000030000035	0.1804700049999965
0.3091399970000026	0.2697100039999967	0.2915900050000033
0.4172700049999989	0.1390900020000032	0.0693399979999967
0.0111699999999999	0.0034199999999984	0.4150600139999980
0.2061800059999968	0.0639299970000025	0.5169799920000031
0.7162500020000024	0.0720800010000033	0.0971200019999969
0.9121900199999970	0.1374000010000032	0.2082500010000032

0.6080600020000020	0.2023700029999986	0.3199799949999971
0.8129900100000000	0.2696099879999991	0.4331600069999979
0.6132100219999970	0.2044000030000035	0.1804700049999965
0.8091400269999980	0.2697100039999967	0.2915900050000033
0.9172700049999989	0.1390900020000032	0.0693399979999967
0.5113999840000005	0.0040400000000034	0.4151499869999995
0.7064099910000010	0.0657000019999998	0.5152699949999970
0.2162500020000024	0.4054200050000034	0.0971200019999969
0.4121899900000017	0.4707300070000002	0.2082500010000032
0.1083699989999971	0.5364099740000015	0.3192299899999966
0.3214699919999973	0.6141999959999964	0.4265199900000027
0.1132100000000023	0.5377399919999988	0.1804700049999965
0.3091399970000026	0.6030499940000027	0.2915900050000033
0.4172700049999989	0.4724200069999966	0.0693399979999967
0.0018999999999991	0.3338199849999981	0.4078600109999968
0.7162500020000024	0.4054200050000034	0.0971200019999969
0.9121900199999970	0.4707300070000002	0.2082500010000032
0.6079300050000001	0.5363500119999998	0.3196099999999973
0.8137900230000028	0.6065099839999988	0.4318799970000029
0.6132100219999970	0.5377399919999988	0.1804700049999965
0.8091400269999980	0.6030499940000027	0.2915900050000033
0.9172700049999989	0.4724200069999966	0.0693399979999967
0.5153499839999967	0.3389999870000011	0.4159300029999997
0.7077400089999983	0.4033299979999967	0.5147299770000018
0.2162500020000024	0.7387499809999980	0.0971200019999969
0.4121899900000017	0.8040599819999983	0.2082500010000032
0.1092500020000031	0.8700299859999987	0.3198699949999977
0.3179000020000018	0.9390599729999991	0.4311099949999999
0.1132100000000023	0.8710700270000018	0.1804700049999965
0.3091399970000026	0.9363800289999986	0.2915900050000033
0.4172700049999989	0.8057600260000015	0.0693399979999967
0.0036199999999980	0.6676599979999978	0.4101600050000016
0.7162500020000024	0.7387499809999980	0.0971200019999969
0.9121900199999970	0.8040599819999983	0.2082500010000032
0.6075699929999985	0.8695999979999982	0.3198499980000022
0.8191800119999968	0.9394699930000030	0.4279299969999997
0.6132100219999970	0.8710700270000018	0.1804700049999965
0.8091400269999980	0.9363800289999986	0.2915900050000033
0.9172700049999989	0.8057600260000015	0.0693399979999967
0.5146399739999978	0.6710900069999965	0.4159600139999995
0.7072200180000010	0.7384600039999967	0.5159699920000023
0.1305200010000007	0.0435100010000014	0.1664600069999977
0.3264499900000004	0.1088199989999978	0.2775900070000006
0.4345799979999967	0.3115299939999971	0.0553399989999974

0.0293400010000013	0.1772899930000023	0.3975299889999988
0.2262199970000012	0.2542400060000034	0.5435600280000017
0.0274700000000010	0.1758199929999975	0.2498099949999997
0.2267699989999983	0.2455500069999985	0.3635900020000022
0.4213500019999969	0.3162199849999965	0.4740999939999995
0.1356000010000002	0.0452000009999978	0.0275599989999975
0.3315300049999976	0.1105099990000014	0.1386799960000005
0.0905399990000006	0.0298299990000004	0.3367600139999993
0.2873699960000025	0.0996899980000023	0.4522100089999981
0.0030000000000001	0.1676699969999973	0.0000000000000000
0.1989399939999998	0.2329799979999976	0.1111199999999997
0.3948799969999968	0.2982900140000027	0.2222500000000025
0.1938599940000003	0.2312899980000012	0.2500300109999998
0.3991700110000025	0.2940100129999976	0.3600299949999979
0.3019900019999966	0.1006600039999981	0.0277799999999999
0.4979299900000029	0.1659799959999972	0.1388999970000029
0.1032600030000026	0.0440200009999998	0.4816800059999977
0.6305199860000030	0.0435100010000014	0.1664600069999977
0.8264499900000004	0.1088199989999978	0.2775900070000006
0.9345800279999992	0.3115299939999971	0.0553399989999974
0.5301700229999966	0.1771599949999967	0.3982200029999987
0.7334100010000029	0.2441799939999996	0.5260000230000017
0.5274699929999969	0.1758199929999975	0.2498099949999997
0.7273899910000026	0.2405399980000027	0.3637399970000033
0.9212499859999994	0.3076300020000033	0.4740799960000004
0.6355999709999978	0.0452000009999978	0.0275599989999975
0.8315299750000023	0.1105099990000014	0.1386799960000005
0.5897600050000023	0.0300099999999972	0.3368000090000010
0.7858499880000025	0.0922100020000016	0.4492900070000019
0.5030000209999983	0.1676699969999973	0.0000000000000000
0.6989399790000022	0.2329799979999976	0.1111199999999997
0.8948799969999968	0.2982900140000027	0.2222500000000025
0.6938599940000003	0.2312899980000012	0.2500300109999998
0.8935300110000028	0.2989200060000030	0.3628000020000002
0.8019899729999977	0.1006600039999981	0.0277799999999999
0.9979299900000029	0.1659799959999972	0.1388999970000029
0.6015099879999966	0.0313599999999994	0.4803699850000029
0.1305200010000007	0.3768399949999974	0.1664600069999977
0.3264499900000004	0.4421499970000013	0.2775900070000006
0.4345799979999967	0.6448600290000002	0.0553399989999974
0.0327900010000022	0.5111200209999964	0.4001100059999985
0.2221799940000011	0.5642099980000026	0.5350300070000031
0.0274700000000010	0.5091599819999999	0.2498099949999997
0.2267799970000013	0.5746200079999966	0.3642700019999978

0.4218499959999988	0.6262800100000021	0.4741500020000018
0.1356000010000002	0.3785299959999975	0.0275599989999975
0.3315300049999976	0.4438399969999978	0.1386799960000005
0.0892499979999997	0.3636200130000020	0.3357999919999983
0.2833000119999980	0.4265899959999970	0.4493899940000006
0.0030000000000001	0.5009999870000001	0.0000000000000000
0.1989399939999998	0.5663099880000004	0.1111199999999997
0.3948799969999968	0.6316300029999979	0.2222500000000025
0.1938599940000003	0.5646200179999994	0.2500300109999998
0.3977900149999982	0.6409800050000030	0.3610199989999998
0.3019900019999966	0.4339999849999998	0.0277799999999999
0.4979299900000029	0.4993099869999966	0.1388999970000029
0.0989599970000015	0.3597100080000004	0.4823699889999986
0.6305199860000030	0.3768399949999974	0.1664600069999977
0.8264499900000004	0.4421499970000013	0.2775900070000006
0.9345800279999992	0.6448600290000002	0.0553399989999974
0.5294799799999979	0.5098099710000028	0.3965699970000003
0.7324299810000028	0.5783500080000010	0.5280699730000009
0.5274699929999969	0.5091599819999999	0.2498099949999997
0.7275300029999983	0.5776100160000013	0.3634499910000031
0.9201700090000031	0.6401600239999965	0.4747500120000012
0.6355999709999978	0.3785299959999975	0.0275599989999975
0.8315299750000023	0.4438399969999978	0.1386799960000005
0.5921099780000034	0.3641299900000021	0.3374899920000018
0.7838699819999988	0.4287199970000017	0.4476999939999970
0.5030000209999983	0.5009999870000001	0.0000000000000000
0.6989399790000022	0.5663099880000004	0.1111199999999997
0.8948799969999968	0.6316300029999979	0.2222500000000025
0.6938599940000003	0.5646200179999994	0.2500300109999998
0.8951299790000036	0.6326900119999976	0.3632099929999981
0.8019899729999977	0.4339999849999998	0.0277799999999999
0.9979299900000029	0.4993099869999966	0.1388999970000029
0.6007800100000011	0.3676500019999978	0.4821299910000008
0.1305200010000007	0.7101699709999991	0.1664600069999977
0.3264499900000004	0.7754799719999994	0.2775900070000006
0.4345799979999967	0.9781900050000019	0.0553399989999974
0.0343700009999992	0.8433200119999995	0.4014999870000011
0.2219299969999966	0.9049199820000027	0.5234400029999975
0.0274700000000010	0.8424900170000029	0.2498099949999997
0.2264199999999974	0.9067500230000007	0.3641999960000035
0.4218600089999995	0.9805999989999989	0.4759800140000010
0.1356000010000002	0.7118700150000024	0.0275599989999975
0.3315300049999976	0.7771800160000026	0.1386799960000005
0.0891700009999994	0.6960999969999975	0.3365100030000008

0.2953099910000034	0.7634400130000003	0.4585100110000013
0.0030000000000001	0.8343300220000032	0.0000000000000000
0.1989399939999998	0.8996499779999994	0.1111199999999997
0.3948799969999968	0.9649599789999996	0.2222500000000025
0.1938599940000003	0.8979499940000011	0.2500300109999998
0.3947600130000026	0.9629499910000021	0.3637000020000016
0.3019900019999966	0.7673299909999969	0.0277799999999999
0.4979299900000029	0.8326399919999972	0.1388899970000029
0.0858400019999976	0.6878200170000000	0.4962199929999969
0.6305199860000030	0.7101699709999991	0.1664600069999977
0.8264499900000004	0.7754799719999994	0.2775900070000006
0.9345800279999992	0.9781900050000019	0.0553399989999974
0.5293200019999986	0.8432400229999999	0.3982400000000013
0.7281600239999975	0.9091899989999987	0.5350099799999981
0.5274699929999969	0.8424900170000029	0.2498099949999997
0.7275300029999983	0.9097899790000028	0.3632099929999981
0.9229699970000027	0.9737499949999986	0.4751099940000003
0.6355999709999978	0.7118700150000024	0.0275599989999975
0.8315299750000023	0.7771800160000026	0.1386799960000005
0.5910500290000016	0.6973900200000003	0.3371599910000000
0.7851799729999982	0.7644600269999984	0.4493600130000033
0.5030000209999983	0.8343300220000032	0.0000000000000000
0.6989399790000022	0.8996499779999994	0.1111199999999997
0.8948799969999968	0.9649599789999996	0.2222500000000025
0.6938599940000003	0.8979499940000011	0.2500300109999998
0.8982899789999976	0.9644500019999995	0.3621599969999991
0.8019899729999977	0.7673299909999969	0.0277799999999999
0.9979299900000029	0.8326399919999972	0.1388899970000029
0.6016399860000021	0.7026500110000029	0.4812200069999975
0.3355813503741829	0.7032477509711588	0.7189752129566419
0.3262563684799729	0.7513256923485689	0.5809928566619944
0.3040102020361017	0.9086400078081548	0.6372877473046101
0.2046100049999993	0.3986899849999972	0.5146800280000008
0.1960200070000013	0.7266899939999973	0.5159500239999986
0.4696555216182571	0.7265397334101639	0.6591894617673141
0.3976419642011585	0.7872110282854275	0.6808543174407579
0.3412046739786831	0.8151891966836371	0.6276660190091827
0.5074567209708025	0.7070218321877000	0.6989720764643630
0.5153922581103134	0.7860343850985471	0.6270451584658354
0.4390424572648758	0.6412227512519022	0.6352835083684686
0.4266624011154317	0.8725675154206860	0.7056063309753111
0.3127614155913621	0.7478750385700188	0.7508896006531774
0.2673664262573045	0.9117377162121523	0.5998405169236430

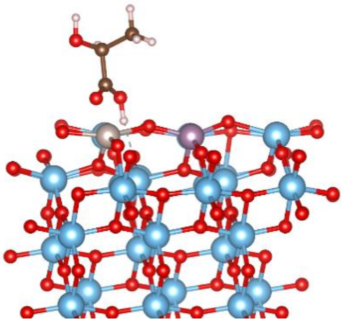

Supplementary Fig. 35c

Ti	O	Mo	Ru	C	H
52	123	1	1	3	6

0.2162500020000024	0.0720800010000033	0.0971200019999969
0.4121899900000017	0.1374000010000032	0.2082500010000032
0.1075700000000026	0.2017599939999997	0.3191800119999968
0.3223899899999978	0.2694700059999988	0.4248200059999974
0.1132100000000023	0.2044000030000035	0.1804700049999965
0.3091399970000026	0.2697100039999967	0.2915900050000033
0.4172700049999989	0.1390900020000032	0.0693399979999967
0.0111699999999999	0.0034199999999984	0.4150600139999980
0.2061800059999968	0.0639299970000025	0.5169799920000031
0.7162500020000024	0.0720800010000033	0.0971200019999969
0.9121900199999970	0.1374000010000032	0.2082500010000032
0.6080600020000020	0.2023700029999986	0.3199799949999971
0.8129900100000000	0.2696099879999991	0.4331600069999979
0.6132100219999970	0.2044000030000035	0.1804700049999965
0.8091400269999980	0.2697100039999967	0.2915900050000033
0.9172700049999989	0.1390900020000032	0.0693399979999967
0.5113999840000005	0.0040400000000034	0.4151499869999995
0.7064099910000010	0.0657000019999998	0.5152699949999970
0.2162500020000024	0.4054200050000034	0.0971200019999969
0.4121899900000017	0.4707300070000002	0.2082500010000032
0.1083699989999971	0.5364099740000015	0.3192299899999966
0.3214699919999973	0.6141999959999964	0.4265199900000027
0.1132100000000023	0.5377399919999988	0.1804700049999965
0.3091399970000026	0.6030499940000027	0.2915900050000033
0.4172700049999989	0.4724200069999966	0.0693399979999967
0.0018999999999991	0.3338199849999981	0.4078600109999968
0.7162500020000024	0.4054200050000034	0.0971200019999969
0.9121900199999970	0.4707300070000002	0.2082500010000032
0.6079300050000001	0.5363500119999998	0.3196099999999973
0.8137900230000028	0.6065099839999988	0.4318799970000029
0.6132100219999970	0.5377399919999988	0.1804700049999965
0.8091400269999980	0.6030499940000027	0.2915900050000033

0.9172700049999989	0.4724200069999966	0.0693399979999967
0.5153499839999967	0.3389999870000011	0.4159300029999997
0.7077400089999983	0.4033299979999967	0.5147299770000018
0.2162500020000024	0.7387499809999980	0.0971200019999969
0.4121899900000017	0.8040599819999983	0.2082500010000032
0.1092500020000031	0.8700299859999987	0.3198699949999977
0.3179000020000018	0.9390599729999991	0.4311099949999999
0.1132100000000023	0.8710700270000018	0.1804700049999965
0.3091399970000026	0.9363800289999986	0.2915900050000033
0.4172700049999989	0.8057600260000015	0.0693399979999967
0.0036199999999980	0.6676599979999978	0.4101600050000016
0.7162500020000024	0.7387499809999980	0.0971200019999969
0.9121900199999970	0.8040599819999983	0.2082500010000032
0.6075699929999985	0.8695999979999982	0.3198499980000022
0.8191800119999968	0.9394699930000030	0.4279299969999997
0.6132100219999970	0.8710700270000018	0.1804700049999965
0.8091400269999980	0.9363800289999986	0.2915900050000033
0.9172700049999989	0.8057600260000015	0.0693399979999967
0.5146399739999978	0.6710900069999965	0.4159600139999995
0.7072200180000010	0.7384600039999967	0.5159699920000023
0.1305200010000007	0.0435100010000014	0.1664600069999977
0.3264499900000004	0.1088199989999978	0.2775900070000006
0.4345799979999967	0.3115299939999971	0.0553399989999974
0.0293400010000013	0.1772899930000023	0.3975299889999988
0.2262199970000012	0.2542400060000034	0.5435600280000017
0.0274700000000010	0.1758199929999975	0.2498099949999997
0.2267699989999983	0.2455500069999985	0.3635900020000022
0.4213500019999969	0.3162199849999965	0.4740999939999995
0.1356000010000002	0.0452000009999978	0.0275599989999975
0.3315300049999976	0.1105099990000014	0.1386799960000005
0.0905399990000006	0.0298299990000004	0.3367600139999993
0.2873699960000025	0.0996899980000023	0.4522100089999981
0.0030000000000001	0.1676699969999973	0.0000000000000000
0.1989399939999998	0.2329799979999976	0.1111199999999997
0.3948799969999968	0.2982900140000027	0.2222500000000025
0.1938599940000003	0.2312899980000012	0.2500300109999998
0.3991700110000025	0.2940100129999976	0.3600299949999979
0.3019900019999966	0.1006600039999981	0.0277799999999999
0.4979299900000029	0.1659799959999972	0.1388899970000029
0.1032600030000026	0.0440200009999998	0.4816800059999977
0.6305199860000030	0.0435100010000014	0.1664600069999977
0.8264499900000004	0.1088199989999978	0.2775900070000006
0.9345800279999992	0.3115299939999971	0.0553399989999974
0.5301700229999966	0.1771599949999967	0.3982200029999987

0.7334100010000029	0.2441799939999996	0.5260000230000017
0.5274699929999969	0.1758199929999975	0.2498099949999997
0.7273899910000026	0.2405399980000027	0.3637399970000033
0.9212499859999994	0.3076300020000033	0.4740799960000004
0.6355999709999978	0.0452000009999978	0.0275599989999975
0.8315299750000023	0.1105099990000014	0.1386799960000005
0.5897600050000023	0.0300099999999972	0.3368000090000010
0.7858499880000025	0.0922100020000016	0.4492900070000019
0.5030000209999983	0.1676699969999973	0.0000000000000000
0.6989399790000022	0.2329799979999976	0.1111199999999997
0.8948799969999968	0.2982900140000027	0.2222500000000025
0.6938599940000003	0.2312899980000012	0.2500300109999998
0.8935300110000028	0.2989200060000030	0.3628000020000002
0.8019899729999977	0.1006600039999981	0.0277799999999999
0.9979299900000029	0.1659799959999972	0.1388999970000029
0.6015099879999966	0.0313599999999994	0.4803699850000029
0.1305200010000007	0.3768399949999974	0.1664600069999977
0.3264499900000004	0.4421499970000013	0.2775900070000006
0.4345799979999967	0.6448600290000002	0.0553399989999974
0.0327900010000022	0.5111200209999964	0.4001100059999985
0.2221799940000011	0.5642099980000026	0.5350300070000031
0.0274700000000010	0.5091599819999999	0.2498099949999997
0.2267799970000013	0.5746200079999966	0.3642700019999978
0.4218499959999988	0.6262800100000021	0.4741500020000018
0.1356000010000002	0.3785299959999975	0.0275599989999975
0.3315300049999976	0.4438399969999978	0.1386799960000005
0.0892499979999997	0.3636200130000020	0.3357999919999983
0.2833000119999980	0.4265899959999970	0.4493899940000006
0.0030000000000001	0.5009999870000001	0.0000000000000000
0.1989399939999998	0.5663099880000004	0.1111199999999997
0.3948799969999968	0.6316300029999979	0.2222500000000025
0.1938599940000003	0.5646200179999994	0.2500300109999998
0.3977900149999982	0.6409800050000030	0.3610199989999998
0.3019900019999966	0.4339999849999998	0.0277799999999999
0.4979299900000029	0.4993099869999966	0.1388999970000029
0.0989599970000015	0.3597100080000004	0.4823699889999986
0.6305199860000030	0.3768399949999974	0.1664600069999977
0.8264499900000004	0.4421499970000013	0.2775900070000006
0.9345800279999992	0.6448600290000002	0.0553399989999974
0.5294799799999979	0.5098099710000028	0.3965699970000003
0.7324299810000028	0.5783500080000010	0.5280699730000009
0.5274699929999969	0.5091599819999999	0.2498099949999997
0.7275300029999983	0.5776100160000013	0.3634499910000031
0.9201700090000031	0.6401600239999965	0.4747500120000012

0.6355999709999978	0.3785299959999975	0.0275599989999975
0.8315299750000023	0.4438399969999978	0.1386799960000005
0.5921099780000034	0.3641299900000021	0.3374899920000018
0.7838699819999988	0.4287199970000017	0.4476999939999970
0.5030000209999983	0.5009999870000001	0.0000000000000000
0.6989399790000022	0.5663099880000004	0.1111199999999997
0.8948799969999968	0.6316300029999979	0.2222500000000025
0.6938599940000003	0.5646200179999994	0.2500300109999998
0.8951299790000036	0.6326900119999976	0.3632099929999981
0.8019899729999977	0.4339999849999998	0.0277799999999999
0.9979299900000029	0.4993099869999966	0.1388999970000029
0.6007800100000011	0.3676500019999978	0.4821299910000008
0.1305200010000007	0.7101699709999991	0.1664600069999977
0.3264499900000004	0.7754799719999994	0.2775900070000006
0.4345799979999967	0.9781900050000019	0.0553399989999974
0.0343700009999992	0.8433200119999995	0.4014999870000011
0.2219299969999966	0.9049199820000027	0.5234400029999975
0.0274700000000010	0.8424900170000029	0.2498099949999997
0.2264199999999974	0.9067500230000007	0.3641999960000035
0.4218600089999995	0.9805999989999989	0.4759800140000010
0.1356000010000002	0.7118700150000024	0.0275599989999975
0.3315300049999976	0.7771800160000026	0.1386799960000005
0.0891700009999994	0.6960999969999975	0.3365100030000008
0.2953099910000034	0.7634400130000003	0.4585100110000013
0.0030000000000001	0.8343300220000032	0.0000000000000000
0.1989399939999998	0.8996499779999994	0.1111199999999997
0.3948799969999968	0.9649599789999996	0.2222500000000025
0.1938599940000003	0.8979499940000011	0.2500300109999998
0.3947600130000026	0.9629499910000021	0.3637000020000016
0.3019900019999966	0.7673299909999969	0.0277799999999999
0.4979299900000029	0.8326399919999972	0.1388999970000029
0.0858400019999976	0.6878200170000000	0.4962199929999969
0.6305199860000030	0.7101699709999991	0.1664600069999977
0.8264499900000004	0.7754799719999994	0.2775900070000006
0.9345800279999992	0.9781900050000019	0.0553399989999974
0.5293200019999986	0.8432400229999999	0.3982400000000013
0.7281600239999975	0.9091899989999987	0.5350099799999981
0.5274699929999969	0.8424900170000029	0.2498099949999997
0.7275300029999983	0.9097899790000028	0.3632099929999981
0.9229699970000027	0.9737499949999986	0.4751099940000003
0.6355999709999978	0.7118700150000024	0.0275599989999975
0.8315299750000023	0.7771800160000026	0.1386799960000005
0.5910500290000016	0.6973900200000003	0.3371599910000000
0.7851799729999982	0.7644600269999984	0.4493600130000033

0.5030000209999983	0.8343300220000032	0.0000000000000000
0.6989399790000022	0.8996499779999994	0.1111199999999997
0.8948799969999968	0.9649599789999996	0.2222500000000025
0.6938599940000003	0.8979499940000011	0.2500300109999998
0.8982899789999976	0.9644500019999995	0.3621599969999991
0.8019899729999977	0.7673299909999969	0.0277799999999999
0.9979299900000029	0.8326399919999972	0.1388899970000029
0.6016399860000021	0.7026500110000029	0.4812200069999975
0.3547760294238086	0.8518295033410059	0.7043241088569402
0.3147030947754978	0.7723668695684326	0.5898608344088601
0.4298758303327304	0.6776797958860201	0.5915538747960579
0.2046100049999993	0.3986899849999972	0.5146800280000008
0.1960200070000013	0.7266899939999973	0.5159500239999986
0.3368620942124341	0.6310801932650572	0.7167002798984641
0.3871400718279556	0.7502160294980486	0.6859106084679273
0.3739833121848857	0.7361034823433794	0.6167745775098467
0.3457743045250215	0.6390320904748678	0.7666457625641251
0.3630123450310425	0.5540375214051070	0.7013961082643200
0.2649283648602249	0.6128550245212975	0.7064316168565914
0.4596802531233277	0.7654403355933118	0.6950432373127670
0.3521936386493916	0.8521846929343080	0.7489492097658518
0.4216481305386344	0.6640768286836919	0.5456655379501442

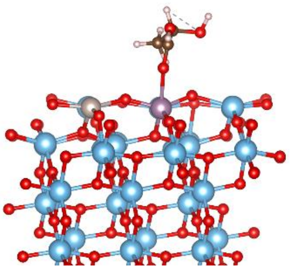

Supplementary Fig. 35d

Ti	O	Mo	Ru	C	H
52	123	1	1	3	6

0.2162500020000024	0.0720800010000033	0.0971200019999969
0.4121899900000017	0.1374000010000032	0.2082500010000032
0.1075700000000026	0.2017599939999997	0.3191800119999968
0.3223899899999978	0.2694700059999988	0.4248200059999974
0.1132100000000023	0.2044000030000035	0.1804700049999965
0.3091399970000026	0.2697100039999967	0.2915900050000033
0.4172700049999989	0.1390900020000032	0.0693399979999967
0.0111699999999999	0.0034199999999984	0.4150600139999980
0.2061800059999968	0.0639299970000025	0.5169799920000031
0.7162500020000024	0.0720800010000033	0.0971200019999969
0.9121900199999970	0.1374000010000032	0.2082500010000032
0.6080600020000020	0.2023700029999986	0.3199799949999971

0.8129900100000000	0.2696099879999991	0.4331600069999979
0.6132100219999970	0.2044000030000035	0.1804700049999965
0.8091400269999980	0.2697100039999967	0.2915900050000033
0.9172700049999989	0.1390900020000032	0.0693399979999967
0.5113999840000005	0.0040400000000034	0.4151499869999995
0.7064099910000010	0.0657000019999998	0.5152699949999970
0.2162500020000024	0.4054200050000034	0.0971200019999969
0.4121899900000017	0.4707300070000002	0.2082500010000032
0.1083699989999971	0.5364099740000015	0.3192299899999966
0.3214699919999973	0.6141999959999964	0.4265199900000027
0.1132100000000023	0.5377399919999988	0.1804700049999965
0.3091399970000026	0.6030499940000027	0.2915900050000033
0.4172700049999989	0.4724200069999966	0.0693399979999967
0.0018999999999991	0.3338199849999981	0.4078600109999968
0.7162500020000024	0.4054200050000034	0.0971200019999969
0.9121900199999970	0.4707300070000002	0.2082500010000032
0.6079300050000001	0.5363500119999998	0.3196099999999973
0.8137900230000028	0.6065099839999988	0.4318799970000029
0.6132100219999970	0.5377399919999988	0.1804700049999965
0.8091400269999980	0.6030499940000027	0.2915900050000033
0.9172700049999989	0.4724200069999966	0.0693399979999967
0.5153499839999967	0.3389999870000011	0.4159300029999997
0.7077400089999983	0.4033299979999967	0.5147299770000018
0.2162500020000024	0.7387499809999980	0.0971200019999969
0.4121899900000017	0.8040599819999983	0.2082500010000032
0.1092500020000031	0.8700299859999987	0.3198699949999977
0.3179000020000018	0.9390599729999991	0.4311099949999999
0.1132100000000023	0.8710700270000018	0.1804700049999965
0.3091399970000026	0.9363800289999986	0.2915900050000033
0.4172700049999989	0.8057600260000015	0.0693399979999967
0.0036199999999980	0.6676599979999978	0.4101600050000016
0.7162500020000024	0.7387499809999980	0.0971200019999969
0.9121900199999970	0.8040599819999983	0.2082500010000032
0.6075699929999985	0.8695999979999982	0.3198499980000022
0.8191800119999968	0.9394699930000030	0.4279299969999997
0.6132100219999970	0.8710700270000018	0.1804700049999965
0.8091400269999980	0.9363800289999986	0.2915900050000033
0.9172700049999989	0.8057600260000015	0.0693399979999967
0.5146399739999978	0.6710900069999965	0.4159600139999995
0.7072200180000010	0.7384600039999967	0.5159699920000023
0.1305200010000007	0.0435100010000014	0.1664600069999977
0.3264499900000004	0.1088199989999978	0.2775900070000006
0.4345799979999967	0.3115299939999971	0.0553399989999974
0.0293400010000013	0.1772899930000023	0.3975299889999988

0.2262199970000012	0.2542400060000034	0.5435600280000017
0.0274700000000010	0.1758199929999975	0.2498099949999997
0.2267699989999983	0.2455500069999985	0.3635900020000022
0.4213500019999969	0.3162199849999965	0.4740999939999995
0.1356000010000002	0.0452000009999978	0.0275599989999975
0.3315300049999976	0.1105099990000014	0.1386799960000005
0.0905399990000006	0.0298299990000004	0.3367600139999993
0.2873699960000025	0.0996899980000023	0.4522100089999981
0.0030000000000001	0.1676699969999973	0.0000000000000000
0.1989399939999998	0.2329799979999976	0.1111199999999997
0.3948799969999968	0.2982900140000027	0.2222500000000025
0.1938599940000003	0.2312899980000012	0.2500300109999998
0.3991700110000025	0.2940100129999976	0.3600299949999979
0.3019900019999966	0.1006600039999981	0.0277799999999999
0.4979299900000029	0.1659799959999972	0.1388999970000029
0.1032600030000026	0.0440200009999998	0.4816800059999977
0.6305199860000030	0.0435100010000014	0.1664600069999977
0.8264499900000004	0.1088199989999978	0.2775900070000006
0.9345800279999992	0.3115299939999971	0.0553399989999974
0.5301700229999966	0.1771599949999967	0.3982200029999987
0.7334100010000029	0.2441799939999996	0.5260000230000017
0.5274699929999969	0.1758199929999975	0.2498099949999997
0.7273899910000026	0.2405399980000027	0.3637399970000033
0.9212499859999994	0.3076300020000033	0.4740799960000004
0.6355999709999978	0.0452000009999978	0.0275599989999975
0.8315299750000023	0.1105099990000014	0.1386799960000005
0.5897600050000023	0.0300099999999972	0.3368000090000010
0.7858499880000025	0.0922100020000016	0.4492900070000019
0.5030000209999983	0.1676699969999973	0.0000000000000000
0.6989399790000022	0.2329799979999976	0.1111199999999997
0.8948799969999968	0.2982900140000027	0.2222500000000025
0.6938599940000003	0.2312899980000012	0.2500300109999998
0.8935300110000028	0.2989200060000030	0.3628000020000002
0.8019899729999977	0.1006600039999981	0.0277799999999999
0.9979299900000029	0.1659799959999972	0.1388999970000029
0.6015099879999966	0.0313599999999994	0.4803699850000029
0.1305200010000007	0.3768399949999974	0.1664600069999977
0.3264499900000004	0.4421499970000013	0.2775900070000006
0.4345799979999967	0.6448600290000002	0.0553399989999974
0.0327900010000022	0.5111200209999964	0.4001100059999985
0.2221799940000011	0.5642099980000026	0.5350300070000031
0.0274700000000010	0.5091599819999999	0.2498099949999997
0.2267799970000013	0.5746200079999966	0.3642700019999978
0.4218499959999988	0.6262800100000021	0.4741500020000018

0.1356000010000002	0.3785299959999975	0.0275599989999975
0.3315300049999976	0.4438399969999978	0.1386799960000005
0.0892499979999997	0.3636200130000020	0.3357999919999983
0.2833000119999980	0.4265899959999970	0.4493899940000006
0.0030000000000001	0.5009999870000001	0.0000000000000000
0.1989399939999998	0.5663099880000004	0.1111199999999997
0.3948799969999968	0.6316300029999979	0.2222500000000025
0.1938599940000003	0.5646200179999994	0.2500300109999998
0.3977900149999982	0.6409800050000030	0.3610199989999998
0.3019900019999966	0.4339999849999998	0.0277799999999999
0.4979299900000029	0.4993099869999966	0.1388999970000029
0.0989599970000015	0.3597100080000004	0.4823699889999986
0.6305199860000030	0.3768399949999974	0.1664600069999977
0.8264499900000004	0.4421499970000013	0.2775900070000006
0.9345800279999992	0.6448600290000002	0.0553399989999974
0.5294799799999979	0.5098099710000028	0.3965699970000003
0.7324299810000028	0.5783500080000010	0.5280699730000009
0.5274699929999969	0.5091599819999999	0.2498099949999997
0.7275300029999983	0.5776100160000013	0.3634499910000031
0.9201700090000031	0.6401600239999965	0.4747500120000012
0.6355999709999978	0.3785299959999975	0.0275599989999975
0.8315299750000023	0.4438399969999978	0.1386799960000005
0.5921099780000034	0.3641299900000021	0.3374899920000018
0.7838699819999988	0.4287199970000017	0.4476999939999970
0.5030000209999983	0.5009999870000001	0.0000000000000000
0.6989399790000022	0.5663099880000004	0.1111199999999997
0.8948799969999968	0.6316300029999979	0.2222500000000025
0.6938599940000003	0.5646200179999994	0.2500300109999998
0.8951299790000036	0.6326900119999976	0.3632099929999981
0.8019899729999977	0.4339999849999998	0.0277799999999999
0.9979299900000029	0.4993099869999966	0.1388999970000029
0.6007800100000011	0.3676500019999978	0.4821299910000008
0.1305200010000007	0.7101699709999991	0.1664600069999977
0.3264499900000004	0.7754799719999994	0.2775900070000006
0.4345799979999967	0.9781900050000019	0.0553399989999974
0.0343700009999992	0.8433200119999995	0.4014999870000011
0.2219299969999966	0.9049199820000027	0.5234400029999975
0.0274700000000010	0.8424900170000029	0.2498099949999997
0.2264199999999974	0.9067500230000007	0.3641999960000035
0.4218600089999995	0.9805999989999989	0.4759800140000010
0.1356000010000002	0.7118700150000024	0.0275599989999975
0.3315300049999976	0.7771800160000026	0.1386799960000005
0.0891700009999994	0.6960999969999975	0.3365100030000008
0.2953099910000034	0.7634400130000003	0.4585100110000013

0.0030000000000001	0.8343300220000032	0.0000000000000000
0.1989399939999998	0.8996499779999994	0.1111199999999997
0.3948799969999968	0.9649599789999996	0.2222500000000025
0.1938599940000003	0.8979499940000011	0.2500300109999998
0.3947600130000026	0.9629499910000021	0.3637000020000016
0.3019900019999966	0.7673299909999969	0.0277799999999999
0.4979299900000029	0.8326399919999972	0.1388999970000029
0.0858400019999976	0.6878200170000000	0.4962199929999969
0.6305199860000030	0.7101699709999991	0.1664600069999977
0.8264499900000004	0.7754799719999994	0.2775900070000006
0.9345800279999992	0.9781900050000019	0.0553399989999974
0.5293200019999986	0.8432400229999999	0.3982400000000013
0.7281600239999975	0.9091899989999987	0.5350099799999981
0.5274699929999969	0.8424900170000029	0.2498099949999997
0.7275300029999983	0.9097899790000028	0.3632099929999981
0.9229699970000027	0.9737499949999986	0.4751099940000003
0.6355999709999978	0.7118700150000024	0.0275599989999975
0.8315299750000023	0.7771800160000026	0.1386799960000005
0.5910500290000016	0.6973900200000003	0.3371599910000000
0.7851799729999982	0.7644600269999984	0.4493600130000033
0.5030000209999983	0.8343300220000032	0.0000000000000000
0.6989399790000022	0.8996499779999994	0.1111199999999997
0.8948799969999968	0.9649599789999996	0.2222500000000025
0.6938599940000003	0.8979499940000011	0.2500300109999998
0.8982899789999976	0.9644500019999995	0.3621599969999991
0.8019899729999977	0.7673299909999969	0.0277799999999999
0.9979299900000029	0.8326399919999972	0.1388999970000029
0.6016399860000021	0.7026500110000029	0.4812200069999975
0.2211332150639743	0.2153995877842810	0.6919330391669432
0.1398751558642427	0.3876520774910539	0.6085617643910759
0.0781883133684263	0.3570074964766802	0.7015723356218398
0.2046100049999993	0.3986899849999972	0.5146800280000008
0.1960200070000013	0.7266899939999973	0.5159500239999986
0.3176830308501445	0.4133636607880019	0.6677264753127228
0.2301190022397891	0.3449403920859124	0.6963011905194442
0.1475522763433379	0.3670221334982734	0.6639036347466297
0.3732945861285237	0.4019216605639831	0.6966601244160600
0.3207739362566054	0.5111258216555302	0.6643066971582435
0.3262953924196377	0.3771385111250468	0.6220727580016507
0.2262945894627010	0.3721596694773172	0.7444588404707109
0.1927233179551239	0.1774428082504211	0.7293831346034514
0.0255139794463352	0.3654667788047227	0.6781227974895087

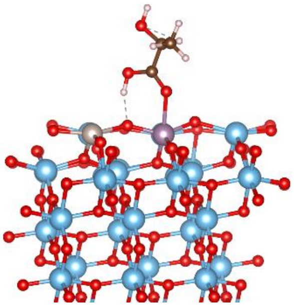

Supplementary Fig. 35e

Ti	O	Mo	Ru	C	H
52	123	1	1	3	6

0.2162500020000024	0.0720800010000033	0.0971200019999969
0.4121899900000017	0.1374000010000032	0.2082500010000032
0.1075700000000026	0.2017599939999997	0.3191800119999968
0.3223899899999978	0.2694700059999988	0.4248200059999974
0.1132100000000023	0.2044000030000035	0.1804700049999965
0.3091399970000026	0.2697100039999967	0.2915900050000033
0.4172700049999989	0.1390900020000032	0.0693399979999967
0.0111699999999999	0.0034199999999984	0.4150600139999980
0.2061800059999968	0.0639299970000025	0.5169799920000031
0.7162500020000024	0.0720800010000033	0.0971200019999969
0.9121900199999970	0.1374000010000032	0.2082500010000032
0.6080600020000020	0.2023700029999986	0.3199799949999971
0.8129900100000000	0.2696099879999991	0.4331600069999979
0.6132100219999970	0.2044000030000035	0.1804700049999965
0.8091400269999980	0.2697100039999967	0.2915900050000033
0.9172700049999989	0.1390900020000032	0.0693399979999967
0.5113999840000005	0.0040400000000034	0.4151499869999995
0.7064099910000010	0.0657000019999998	0.5152699949999970
0.2162500020000024	0.4054200050000034	0.0971200019999969
0.4121899900000017	0.4707300070000002	0.2082500010000032
0.1083699989999971	0.5364099740000015	0.3192299899999966
0.3214699919999973	0.6141999959999964	0.4265199900000027
0.1132100000000023	0.5377399919999988	0.1804700049999965
0.3091399970000026	0.6030499940000027	0.2915900050000033
0.4172700049999989	0.4724200069999966	0.0693399979999967
0.0018999999999991	0.3338199849999981	0.4078600109999968
0.7162500020000024	0.4054200050000034	0.0971200019999969
0.9121900199999970	0.4707300070000002	0.2082500010000032
0.6079300050000001	0.5363500119999998	0.3196099999999973
0.8137900230000028	0.6065099839999988	0.4318799970000029
0.6132100219999970	0.5377399919999988	0.1804700049999965
0.8091400269999980	0.6030499940000027	0.2915900050000033
0.9172700049999989	0.4724200069999966	0.0693399979999967
0.5153499839999967	0.3389999870000011	0.4159300029999997

0.7077400089999983	0.4033299979999967	0.5147299770000018
0.2162500020000024	0.7387499809999980	0.0971200019999969
0.4121899900000017	0.8040599819999983	0.2082500010000032
0.1092500020000031	0.8700299859999987	0.3198699949999977
0.3179000020000018	0.9390599729999991	0.4311099949999999
0.1132100000000023	0.8710700270000018	0.1804700049999965
0.3091399970000026	0.9363800289999986	0.2915900050000033
0.4172700049999989	0.8057600260000015	0.0693399979999967
0.0036199999999980	0.6676599979999978	0.4101600050000016
0.7162500020000024	0.7387499809999980	0.0971200019999969
0.9121900199999970	0.8040599819999983	0.2082500010000032
0.6075699929999985	0.8695999979999982	0.3198499980000022
0.8191800119999968	0.9394699930000030	0.4279299969999997
0.6132100219999970	0.8710700270000018	0.1804700049999965
0.8091400269999980	0.9363800289999986	0.2915900050000033
0.9172700049999989	0.8057600260000015	0.0693399979999967
0.5146399739999978	0.6710900069999965	0.4159600139999995
0.7072200180000010	0.7384600039999967	0.5159699920000023
0.1305200010000007	0.0435100010000014	0.1664600069999977
0.3264499900000004	0.1088199989999978	0.2775900070000006
0.4345799979999967	0.3115299939999971	0.0553399989999974
0.0293400010000013	0.1772899930000023	0.3975299889999988
0.2262199970000012	0.2542400060000034	0.5435600280000017
0.0274700000000010	0.1758199929999975	0.2498099949999997
0.2267699989999983	0.2455500069999985	0.3635900020000022
0.4213500019999969	0.3162199849999965	0.4740999939999995
0.1356000010000002	0.0452000009999978	0.0275599989999975
0.3315300049999976	0.1105099990000014	0.1386799960000005
0.0905399990000006	0.0298299990000004	0.3367600139999993
0.2873699960000025	0.0996899980000023	0.4522100089999981
0.0030000000000001	0.1676699969999973	0.0000000000000000
0.1989399939999998	0.2329799979999976	0.1111199999999997
0.3948799969999968	0.2982900140000027	0.2222500000000025
0.1938599940000003	0.2312899980000012	0.2500300109999998
0.3991700110000025	0.2940100129999976	0.3600299949999979
0.3019900019999966	0.1006600039999981	0.0277799999999999
0.4979299900000029	0.1659799959999972	0.1388899970000029
0.1032600030000026	0.0440200009999998	0.4816800059999977
0.6305199860000030	0.0435100010000014	0.1664600069999977
0.8264499900000004	0.1088199989999978	0.2775900070000006
0.9345800279999992	0.3115299939999971	0.0553399989999974
0.5301700229999966	0.1771599949999967	0.3982200029999987
0.7334100010000029	0.2441799939999996	0.5260000230000017
0.5274699929999969	0.1758199929999975	0.2498099949999997

0.7273899910000026	0.2405399980000027	0.3637399970000033
0.9212499859999994	0.3076300020000033	0.4740799960000004
0.6355999709999978	0.0452000009999978	0.0275599989999975
0.8315299750000023	0.1105099990000014	0.1386799960000005
0.5897600050000023	0.0300099999999972	0.3368000090000010
0.7858499880000025	0.0922100020000016	0.4492900070000019
0.5030000209999983	0.1676699969999973	0.0000000000000000
0.6989399790000022	0.2329799979999976	0.1111199999999997
0.8948799969999968	0.2982900140000027	0.2222500000000025
0.6938599940000003	0.2312899980000012	0.2500300109999998
0.8935300110000028	0.2989200060000030	0.3628000020000002
0.8019899729999977	0.1006600039999981	0.0277799999999999
0.9979299900000029	0.1659799959999972	0.1388999970000029
0.6015099879999966	0.0313599999999994	0.4803699850000029
0.1305200010000007	0.3768399949999974	0.1664600069999977
0.3264499900000004	0.4421499970000013	0.2775900070000006
0.4345799979999967	0.6448600290000002	0.0553399989999974
0.0327900010000022	0.5111200209999964	0.4001100059999985
0.2221799940000011	0.5642099980000026	0.5350300070000031
0.0274700000000010	0.5091599819999999	0.2498099949999997
0.2267799970000013	0.5746200079999966	0.3642700019999978
0.4218499959999988	0.6262800100000021	0.4741500020000018
0.1356000010000002	0.3785299959999975	0.0275599989999975
0.3315300049999976	0.4438399969999978	0.1386799960000005
0.0892499979999997	0.3636200130000020	0.3357999919999983
0.2833000119999980	0.4265899959999970	0.4493899940000006
0.0030000000000001	0.5009999870000001	0.0000000000000000
0.1989399939999998	0.5663099880000004	0.1111199999999997
0.3948799969999968	0.6316300029999979	0.2222500000000025
0.1938599940000003	0.5646200179999994	0.2500300109999998
0.3977900149999982	0.6409800050000030	0.3610199989999998
0.3019900019999966	0.4339999849999998	0.0277799999999999
0.4979299900000029	0.4993099869999966	0.1388999970000029
0.0989599970000015	0.3597100080000004	0.4823699889999986
0.6305199860000030	0.3768399949999974	0.1664600069999977
0.8264499900000004	0.4421499970000013	0.2775900070000006
0.9345800279999992	0.6448600290000002	0.0553399989999974
0.5294799799999979	0.5098099710000028	0.3965699970000003
0.7324299810000028	0.5783500080000010	0.5280699730000009
0.5274699929999969	0.5091599819999999	0.2498099949999997
0.7275300029999983	0.5776100160000013	0.3634499910000031
0.9201700090000031	0.6401600239999965	0.4747500120000012
0.6355999709999978	0.3785299959999975	0.0275599989999975
0.8315299750000023	0.4438399969999978	0.1386799960000005

0.5921099780000034	0.3641299900000021	0.3374899920000018
0.7838699819999988	0.4287199970000017	0.4476999939999970
0.5030000209999983	0.5009999870000001	0.0000000000000000
0.6989399790000022	0.5663099880000004	0.1111199999999997
0.8948799969999968	0.6316300029999979	0.2222500000000025
0.6938599940000003	0.5646200179999994	0.2500300109999998
0.8951299790000036	0.6326900119999976	0.3632099929999981
0.8019899729999977	0.4339999849999998	0.0277799999999999
0.9979299900000029	0.4993099869999966	0.1388999970000029
0.6007800100000011	0.3676500019999978	0.4821299910000008
0.1305200010000007	0.7101699709999991	0.1664600069999977
0.3264499900000004	0.7754799719999994	0.2775900070000006
0.4345799979999967	0.9781900050000019	0.0553399989999974
0.0343700009999992	0.8433200119999995	0.4014999870000011
0.2219299969999966	0.9049199820000027	0.5234400029999975
0.0274700000000010	0.8424900170000029	0.2498099949999997
0.2264199999999974	0.9067500230000007	0.3641999960000035
0.4218600089999995	0.9805999989999989	0.4759800140000010
0.1356000010000002	0.7118700150000024	0.0275599989999975
0.3315300049999976	0.7771800160000026	0.1386799960000005
0.0891700009999994	0.6960999969999975	0.3365100030000008
0.2953099910000034	0.7634400130000003	0.4585100110000013
0.0030000000000001	0.8343300220000032	0.0000000000000000
0.1989399939999998	0.8996499779999994	0.1111199999999997
0.3948799969999968	0.9649599789999996	0.2222500000000025
0.1938599940000003	0.8979499940000011	0.2500300109999998
0.3947600130000026	0.9629499910000021	0.3637000020000016
0.3019900019999966	0.7673299909999969	0.0277799999999999
0.4979299900000029	0.8326399919999972	0.1388999970000029
0.0858400019999976	0.6878200170000000	0.4962199929999969
0.6305199860000030	0.7101699709999991	0.1664600069999977
0.8264499900000004	0.7754799719999994	0.2775900070000006
0.9345800279999992	0.9781900050000019	0.0553399989999974
0.5293200019999986	0.8432400229999999	0.3982400000000013
0.7281600239999975	0.9091899989999987	0.5350099799999981
0.5274699929999969	0.8424900170000029	0.2498099949999997
0.7275300029999983	0.9097899790000028	0.3632099929999981
0.9229699970000027	0.9737499949999986	0.4751099940000003
0.6355999709999978	0.7118700150000024	0.0275599989999975
0.8315299750000023	0.7771800160000026	0.1386799960000005
0.5910500290000016	0.6973900200000003	0.3371599910000000
0.7851799729999982	0.7644600269999984	0.4493600130000033
0.5030000209999983	0.8343300220000032	0.0000000000000000
0.6989399790000022	0.8996499779999994	0.1111199999999997

0.8948799969999968	0.9649599789999996	0.2222500000000025
0.6938599940000003	0.8979499940000011	0.2500300109999998
0.8982899789999976	0.9644500019999995	0.3621599969999991
0.8019899729999977	0.7673299909999969	0.0277799999999999
0.9979299900000029	0.8326399919999972	0.1388999970000029
0.6016399860000021	0.7026500110000029	0.4812200069999975
0.1919729705046724	0.5010168361877368	0.7654424063941350
0.1607921974990942	0.3859182288361641	0.6137205751393908
0.2415892625227802	0.5606356367786098	0.6565967559393323
0.2046100049999993	0.3986899849999972	0.5146800280000008
0.1960200070000013	0.7266899939999973	0.5159500239999986
0.0522772393604564	0.3668335038342732	0.7257036001943746
0.1549044606971575	0.4073227226491407	0.7235079289909926
0.1864148169839394	0.4520745404702970	0.6599106273226262
0.0315162849977991	0.3285124133301177	0.7711428948661454
0.0264257751393989	0.2970071116880199	0.6909150792156112
0.0236348512903246	0.4454121018214617	0.7182893118128649
0.1822929048319726	0.3262244007060906	0.7305516269044007
0.1749837125568022	0.4727499471271551	0.8069672979178042
0.2517229413285134	0.5811209840142207	0.6121857204754201

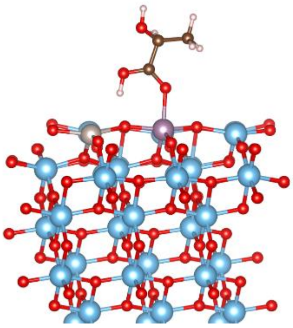

Supplementary Fig. 35f

Ti O Mo Ru C H

52 123 1 1 3 6

0.2162500020000024	0.0720800010000033	0.0971200019999969
0.4121899900000017	0.1374000010000032	0.2082500010000032
0.1088563280548426	0.2026996703663542	0.3187529341509733
0.3211903906142102	0.2729759366070897	0.4261084609855771
0.1132100000000023	0.2044000030000035	0.1804700049999965
0.3091399970000026	0.2697100039999967	0.2915900050000033
0.4172700049999989	0.1390900020000032	0.0693399979999967
0.0118033340918309	0.0039463800068074	0.4124172966111677
0.2101451401689200	0.0647644133536818	0.5080356182401498
0.7162500020000024	0.0720800010000033	0.0971200019999969
0.9121900199999970	0.1374000010000032	0.2082500010000032
0.6085186549100440	0.2025997186275920	0.3189767462175978

0.8156852996836687	0.2708825034106702	0.4310671016522917
0.6132100219999970	0.2044000030000035	0.1804700049999965
0.8091400269999980	0.2697100039999967	0.2915900050000033
0.9172700049999989	0.1390900020000032	0.0693399979999967
0.5131614321782656	0.0044145278481812	0.4124021827858256
0.7086801245950802	0.0670219512177905	0.5115007486355968
0.2162500020000024	0.4054200050000034	0.0971200019999969
0.4121899900000017	0.4707300070000002	0.2082500010000032
0.1093879001036102	0.5363095964805146	0.3186409582687385
0.3229079018185875	0.6092142588848082	0.4258059931011137
0.1132100000000023	0.5377399919999988	0.1804700049999965
0.3091399970000026	0.6030499940000027	0.2915900050000033
0.4172700049999989	0.4724200069999966	0.0693399979999967
0.0038402045264056	0.3348979304003064	0.4069134418729807
0.7162500020000024	0.4054200050000034	0.0971200019999969
0.9121900199999970	0.4707300070000002	0.2082500010000032
0.6085694900671573	0.5360546417350016	0.3189044713136074
0.8146703153302017	0.6059514267285996	0.4308378895809173
0.6132100219999970	0.5377399919999988	0.1804700049999965
0.8091400269999980	0.6030499940000027	0.2915900050000033
0.9172700049999989	0.4724200069999966	0.0693399979999967
0.5133513094304439	0.3378357430393766	0.4127492771143188
0.7079281689970643	0.4024148094391324	0.5105394444250076
0.2162500020000024	0.7387499809999980	0.0971200019999969
0.4121899900000017	0.8040599819999983	0.2082500010000032
0.1094888933516234	0.8698946118264035	0.3189843419134324
0.3215339296421359	0.9388546050206672	0.4276076501744531
0.1132100000000023	0.8710700270000018	0.1804700049999965
0.3091399970000026	0.9363800289999986	0.2915900050000033
0.4172700049999989	0.8057600260000015	0.0693399979999967
0.0035489534690991	0.6675274722693997	0.4071340365046697
0.7162500020000024	0.7387499809999980	0.0971200019999969
0.9121900199999970	0.8040599819999983	0.2082500010000032
0.6083091455084373	0.8694140985684097	0.3190176468275276
0.8209919492678801	0.9405428738078233	0.4265848158960300
0.6132100219999970	0.8710700270000018	0.1804700049999965
0.8091400269999980	0.9363800289999986	0.2915900050000033
0.9172700049999989	0.8057600260000015	0.0693399979999967
0.5148325039689625	0.6709951048249277	0.4131457894376784
0.7091153349893390	0.7393234247455555	0.5117301046357013
0.1305200010000007	0.0435100010000014	0.1664600069999977
0.3264499900000004	0.1088199989999978	0.2775900070000006
0.4345799979999967	0.3115299939999971	0.0553399989999974
0.0318582455150206	0.1790206025351518	0.3976944990621073

0.2337539504027684	0.2438771084032220	0.5247547659190694
0.0274700000000010	0.1758199929999975	0.2498099949999997
0.2267716227928946	0.2481422831353903	0.3630960518725510
0.4226332852798978	0.3124127312037150	0.4733337332847504
0.1356000010000002	0.0452000009999978	0.0275599989999975
0.3315300049999976	0.1105099990000014	0.1386799960000005
0.0924994975798785	0.0306948540801417	0.3356779626022153
0.2895753062869099	0.0973747621582911	0.4427119426525487
0.0030000000000001	0.1676699969999973	0.0000000000000000
0.1989399939999998	0.2329799979999976	0.1111199999999997
0.3948799969999968	0.2982900140000027	0.2222500000000025
0.1938599940000003	0.2312899980000012	0.2500300109999998
0.3981583613492803	0.2988128266083023	0.3600278149500667
0.3019900019999966	0.1006600039999981	0.0277799999999999
0.4979299900000029	0.1659799959999972	0.1388999970000029
0.1037961087510282	0.0365460882555298	0.4773017692546724
0.6305199860000030	0.0435100010000014	0.1664600069999977
0.8264499900000004	0.1088199989999978	0.2775900070000006
0.9345800279999992	0.3115299939999971	0.0553399989999974
0.5289976483353246	0.1767040955700862	0.3954414709589956
0.7351763007055527	0.2446800516611456	0.5229278927291838
0.5274699929999969	0.1758199929999975	0.2498099949999997
0.7284995005082661	0.2407761332934163	0.3633077725864764
0.9235106923317197	0.3092612326493162	0.4729268963529671
0.6355999709999978	0.0452000009999978	0.0275599989999975
0.8315299750000023	0.1105099990000014	0.1386799960000005
0.5925998027556589	0.0307591709468864	0.3355221023425655
0.7888661625911960	0.0941774914952854	0.4463255853802253
0.5030000209999983	0.1676699969999973	0.0000000000000000
0.6989399790000022	0.2329799979999976	0.1111199999999997
0.8948799969999968	0.2982900140000027	0.2222500000000025
0.6938599940000003	0.2312899980000012	0.2500300109999998
0.8963862293633463	0.3001175316700667	0.3612740288663751
0.8019899729999977	0.1006600039999981	0.0277799999999999
0.9979299900000029	0.1659799959999972	0.1388999970000029
0.6039709437590528	0.0328743110285463	0.4766571789189673
0.1305200010000007	0.3768399949999974	0.1664600069999977
0.3264499900000004	0.4421499970000013	0.2775900070000006
0.4345799979999967	0.6448600290000002	0.0553399989999974
0.0355225257026864	0.5118040745325330	0.4004436785096803
0.2244899793861547	0.5753096843521974	0.5260249248022681
0.0274700000000010	0.5091599819999999	0.2498099949999997
0.2275051001296379	0.5750828871837917	0.3638495745410245
0.4241011617639917	0.6296933965889965	0.4732725226386943

0.1356000010000002	0.3785299959999975	0.0275599989999975
0.3315300049999976	0.4438399969999978	0.1386799960000005
0.0902226231396769	0.3642980126853330	0.3348239927219099
0.2844981983434454	0.4293094650891695	0.4505688797118045
0.0030000000000001	0.5009999870000001	0.0000000000000000
0.1989399939999998	0.5663099880000004	0.1111199999999997
0.3948799969999968	0.6316300029999979	0.2222500000000025
0.1938599940000003	0.5646200179999994	0.2500300109999998
0.3990113177643398	0.6362608253920488	0.3597855973810416
0.3019900019999966	0.4339999849999998	0.0277799999999999
0.4979299900000029	0.4993099869999966	0.1388999970000029
0.1003769453572955	0.3620027434856165	0.4878265980111945
0.6305199860000030	0.3768399949999974	0.1664600069999977
0.8264499900000004	0.4421499970000013	0.2775900070000006
0.9345800279999992	0.6448600290000002	0.0553399989999974
0.5298169992900835	0.5095011895155537	0.3954125093530466
0.7343848320641552	0.5785536131917532	0.5227271162531738
0.5274699929999969	0.5091599819999999	0.2498099949999997
0.7277567953678959	0.5775668901906902	0.3630684424305665
0.9214256947812879	0.6385904460339341	0.4729130639869481
0.6355999709999978	0.3785299959999975	0.0275599989999975
0.8315299750000023	0.4438399969999978	0.1386799960000005
0.5934339625241042	0.3642955058627280	0.3359246555159784
0.7852005822456483	0.4281713930652280	0.4442697532252122
0.5030000209999983	0.5009999870000001	0.0000000000000000
0.6989399790000022	0.5663099880000004	0.1111199999999997
0.8948799969999968	0.6316300029999979	0.2222500000000025
0.6938599940000003	0.5646200179999994	0.2500300109999998
0.8956738599571814	0.6310789301836457	0.3612777811081378
0.8019899729999977	0.4339999849999998	0.0277799999999999
0.9979299900000029	0.4993099869999966	0.1388999970000029
0.6011755716277967	0.3660282285285334	0.4785783133398804
0.1305200010000007	0.7101699709999991	0.1664600069999977
0.3264499900000004	0.7754799719999994	0.2775900070000006
0.4345799979999967	0.9781900050000019	0.0553399989999974
0.0322940965790609	0.8424141426257027	0.3983165955973645
0.2307968914501356	0.9069416457841960	0.5172036702971089
0.0274700000000010	0.8424900170000029	0.2498099949999997
0.2269745939497989	0.9031549385285427	0.3634088273472654
0.4244756830072295	0.9800603101164924	0.4735992763487130
0.1356000010000002	0.7118700150000024	0.0275599989999975
0.3315300049999976	0.7771800160000026	0.1386799960000005
0.0901056989306559	0.6958017836883358	0.3352971663825648
0.2984498059338785	0.7629976435748979	0.4536249837310319

0.0030000000000001	0.8343300220000032	0.0000000000000000
0.1989399939999998	0.8996499779999994	0.1111199999999997
0.3948799969999968	0.9649599789999996	0.2222500000000025
0.1938599940000003	0.8979499940000011	0.2500300109999998
0.3978675280529037	0.9632561740014181	0.3603875501814567
0.3019900019999966	0.7673299909999969	0.0277799999999999
0.4979299900000029	0.8326399919999972	0.1388999970000029
0.0862690241017854	0.6987954501896729	0.4911489237900669
0.6305199860000030	0.7101699709999991	0.1664600069999977
0.8264499900000004	0.7754799719999994	0.2775900070000006
0.9345800279999992	0.9781900050000019	0.0553399989999974
0.5289704661013899	0.8426626295892327	0.3960518159359671
0.7312775185294310	0.9105670699015195	0.5303468732572483
0.5274699929999969	0.8424900170000029	0.2498099949999997
0.7280463415983232	0.9096203938993529	0.3629589001182839
0.9245707365162843	0.9745880551130092	0.4734555557887993
0.6355999709999978	0.7118700150000024	0.0275599989999975
0.8315299750000023	0.7771800160000026	0.1386799960000005
0.5925701888010970	0.6974952586510592	0.3357725133095134
0.7880262067385132	0.7652602481846065	0.4460730308764776
0.5030000209999983	0.8343300220000032	0.0000000000000000
0.6989399790000022	0.8996499779999994	0.1111199999999997
0.8948799969999968	0.9649599789999996	0.2222500000000025
0.6938599940000003	0.8979499940000011	0.2500300109999998
0.8994450072654049	0.9664933480544255	0.3603399402969050
0.8019899729999977	0.7673299909999969	0.0277799999999999
0.9979299900000029	0.8326399919999972	0.1388999970000029
0.6035289583311371	0.7037714526985306	0.4777116877751744
0.1469879321123378	0.4983871801823251	0.7542816958580508
0.2018498839371667	0.3880228619914327	0.6150370976744421
0.2221575960669898	0.5813099384296049	0.6489598708026445
0.2064765580220797	0.3987569895474484	0.5183328919867284
0.1968422994253241	0.7318224419937881	0.5072535230593035
0.1490698492641514	0.2858863408262302	0.7300716830555896
0.1935051807503966	0.4214406022037423	0.7243802470652435
0.2055772097726442	0.4645734731516201	0.6583266531323331
0.1450149492090790	0.2606414580906111	0.7787297922321856
0.1885387462317234	0.2309508131159405	0.7065132269194663
0.0810195468278824	0.2650418263275726	0.7110539513119234
0.2637602008672612	0.4385127005779900	0.7413819498452568
0.1526249891863351	0.4903648040551355	0.7985276487058866
0.2291233897333989	0.5978314449390529	0.6034897935598391

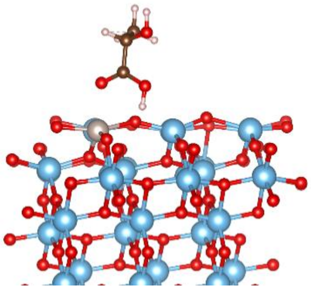

Supplementary Fig. 36a

Ti	O	Ru	C	H
53	123	1	3	6

0.2162500020000024	0.0720800010000033	0.0971200019999969
0.4121899900000017	0.1374000010000032	0.2082500010000032
0.1075700000000026	0.2017599939999997	0.3191800119999968
0.3223899899999978	0.2694700059999988	0.4248200059999974
0.1132100000000023	0.2044000030000035	0.1804700049999965
0.3091399970000026	0.2697100039999967	0.2915900050000033
0.4172700049999989	0.1390900020000032	0.0693399979999967
0.0111699999999999	0.0034199999999984	0.4150600139999980
0.2061800059999968	0.0639299970000025	0.5169799920000031
0.7162500020000024	0.0720800010000033	0.0971200019999969
0.9121900199999970	0.1374000010000032	0.2082500010000032
0.6080600020000020	0.2023700029999986	0.3199799949999971
0.8129900100000000	0.2696099879999991	0.4331600069999979
0.6132100219999970	0.2044000030000035	0.1804700049999965
0.8091400269999980	0.2697100039999967	0.2915900050000033
0.9172700049999989	0.1390900020000032	0.0693399979999967
0.5113999840000005	0.0040400000000034	0.4151499869999995
0.7064099910000010	0.0657000019999998	0.5152699949999970
0.2162500020000024	0.4054200050000034	0.0971200019999969
0.4121899900000017	0.4707300070000002	0.2082500010000032
0.1083699989999971	0.5364099740000015	0.3192299899999966
0.3214699919999973	0.6141999959999964	0.4265199900000027
0.1132100000000023	0.5377399919999988	0.1804700049999965
0.3091399970000026	0.6030499940000027	0.2915900050000033
0.4172700049999989	0.4724200069999966	0.0693399979999967
0.0018999999999991	0.3338199849999981	0.4078600109999968
0.7162500020000024	0.4054200050000034	0.0971200019999969
0.9121900199999970	0.4707300070000002	0.2082500010000032
0.6079300050000001	0.5363500119999998	0.3196099999999973
0.8137900230000028	0.6065099839999988	0.4318799970000029
0.6132100219999970	0.5377399919999988	0.1804700049999965
0.8091400269999980	0.6030499940000027	0.2915900050000033
0.9172700049999989	0.4724200069999966	0.0693399979999967
0.5153499839999967	0.3389999870000011	0.4159300029999997

0.7077400089999983	0.4033299979999967	0.5147299770000018
0.2162500020000024	0.7387499809999980	0.0971200019999969
0.4121899900000017	0.8040599819999983	0.2082500010000032
0.1092500020000031	0.8700299859999987	0.3198699949999977
0.3179000020000018	0.9390599729999991	0.4311099949999999
0.1132100000000023	0.8710700270000018	0.1804700049999965
0.3091399970000026	0.9363800289999986	0.2915900050000033
0.4172700049999989	0.8057600260000015	0.0693399979999967
0.0036199999999980	0.6676599979999978	0.4101600050000016
0.7162500020000024	0.7387499809999980	0.0971200019999969
0.9121900199999970	0.8040599819999983	0.2082500010000032
0.6075699929999985	0.8695999979999982	0.3198499980000022
0.8191800119999968	0.9394699930000030	0.4279299969999997
0.6132100219999970	0.8710700270000018	0.1804700049999965
0.8091400269999980	0.9363800289999986	0.2915900050000033
0.9172700049999989	0.8057600260000015	0.0693399979999967
0.5146399739999978	0.6710900069999965	0.4159600139999995
0.7072200180000010	0.7384600039999967	0.5159699920000023
0.2046100049999993	0.3986899849999972	0.5146800280000008
0.1305200010000007	0.0435100010000014	0.1664600069999977
0.3264499900000004	0.1088199989999978	0.2775900070000006
0.4345799979999967	0.3115299939999971	0.0553399989999974
0.0293400010000013	0.1772899930000023	0.3975299889999988
0.2262199970000012	0.2542400060000034	0.5435600280000017
0.0274700000000010	0.1758199929999975	0.2498099949999997
0.2267699989999983	0.2455500069999985	0.3635900020000022
0.4213500019999969	0.3162199849999965	0.4740999939999995
0.1356000010000002	0.0452000009999978	0.0275599989999975
0.3315300049999976	0.1105099990000014	0.1386799960000005
0.0905399990000006	0.0298299990000004	0.3367600139999993
0.2873699960000025	0.0996899980000023	0.4522100089999981
0.0030000000000001	0.1676699969999973	0.0000000000000000
0.1989399939999998	0.2329799979999976	0.1111199999999997
0.3948799969999968	0.2982900140000027	0.2222500000000025
0.1938599940000003	0.2312899980000012	0.2500300109999998
0.3991700110000025	0.2940100129999976	0.3600299949999979
0.3019900019999966	0.1006600039999981	0.0277799999999999
0.4979299900000029	0.1659799959999972	0.1388999970000029
0.1032600030000026	0.0440200009999998	0.4816800059999977
0.6305199860000030	0.0435100010000014	0.1664600069999977
0.8264499900000004	0.1088199989999978	0.2775900070000006
0.9345800279999992	0.3115299939999971	0.0553399989999974
0.5301700229999966	0.1771599949999967	0.3982200029999987
0.7334100010000029	0.2441799939999996	0.5260000230000017

0.5274699929999969	0.1758199929999975	0.2498099949999997
0.7273899910000026	0.2405399980000027	0.3637399970000033
0.9212499859999994	0.3076300020000033	0.4740799960000004
0.6355999709999978	0.0452000009999978	0.0275599989999975
0.8315299750000023	0.1105099990000014	0.1386799960000005
0.5897600050000023	0.0300099999999972	0.3368000090000010
0.7858499880000025	0.0922100020000016	0.4492900070000019
0.5030000209999983	0.1676699969999973	0.0000000000000000
0.6989399790000022	0.2329799979999976	0.1111199999999997
0.8948799969999968	0.2982900140000027	0.2222500000000025
0.6938599940000003	0.2312899980000012	0.2500300109999998
0.8935300110000028	0.2989200060000030	0.3628000020000002
0.8019899729999977	0.1006600039999981	0.0277799999999999
0.9979299900000029	0.1659799959999972	0.1388999970000029
0.6015099879999966	0.0313599999999994	0.4803699850000029
0.1305200010000007	0.3768399949999974	0.1664600069999977
0.3264499900000004	0.4421499970000013	0.2775900070000006
0.4345799979999967	0.6448600290000002	0.0553399989999974
0.0327900010000022	0.5111200209999964	0.4001100059999985
0.2221799940000011	0.5642099980000026	0.5350300070000031
0.0274700000000010	0.5091599819999999	0.2498099949999997
0.2267799970000013	0.5746200079999966	0.3642700019999978
0.4218499959999988	0.6262800100000021	0.4741500020000018
0.1356000010000002	0.3785299959999975	0.0275599989999975
0.3315300049999976	0.4438399969999978	0.1386799960000005
0.0892499979999997	0.3636200130000020	0.3357999919999983
0.2833000119999980	0.4265899959999970	0.4493899940000006
0.0030000000000001	0.5009999870000001	0.0000000000000000
0.1989399939999998	0.5663099880000004	0.1111199999999997
0.3948799969999968	0.6316300029999979	0.2222500000000025
0.1938599940000003	0.5646200179999994	0.2500300109999998
0.3977900149999982	0.6409800050000030	0.3610199989999998
0.3019900019999966	0.4339999849999998	0.0277799999999999
0.4979299900000029	0.4993099869999966	0.1388999970000029
0.0989599970000015	0.3597100080000004	0.4823699889999986
0.6305199860000030	0.3768399949999974	0.1664600069999977
0.8264499900000004	0.4421499970000013	0.2775900070000006
0.9345800279999992	0.6448600290000002	0.0553399989999974
0.5294799799999979	0.5098099710000028	0.3965699970000003
0.7324299810000028	0.5783500080000010	0.5280699730000009
0.5274699929999969	0.5091599819999999	0.2498099949999997
0.7275300029999983	0.5776100160000013	0.3634499910000031
0.9201700090000031	0.6401600239999965	0.4747500120000012
0.6355999709999978	0.3785299959999975	0.0275599989999975

0.8315299750000023	0.4438399969999978	0.1386799960000005
0.5921099780000034	0.3641299900000021	0.3374899920000018
0.7838699819999988	0.4287199970000017	0.4476999939999970
0.5030000209999983	0.5009999870000001	0.0000000000000000
0.6989399790000022	0.5663099880000004	0.1111199999999997
0.8948799969999968	0.6316300029999979	0.2222500000000025
0.6938599940000003	0.5646200179999994	0.2500300109999998
0.8951299790000036	0.6326900119999976	0.3632099929999981
0.8019899729999977	0.4339999849999998	0.0277799999999999
0.9979299900000029	0.4993099869999966	0.1388999970000029
0.6007800100000011	0.3676500019999978	0.4821299910000008
0.1305200010000007	0.7101699709999991	0.1664600069999977
0.3264499900000004	0.7754799719999994	0.2775900070000006
0.4345799979999967	0.9781900050000019	0.0553399989999974
0.0343700009999992	0.8433200119999995	0.4014999870000011
0.2219299969999966	0.9049199820000027	0.5234400029999975
0.0274700000000010	0.8424900170000029	0.2498099949999997
0.2264199999999974	0.9067500230000007	0.3641999960000035
0.4218600089999995	0.9805999989999989	0.4759800140000010
0.1356000010000002	0.7118700150000024	0.0275599989999975
0.3315300049999976	0.7771800160000026	0.1386799960000005
0.0891700009999994	0.6960999969999975	0.3365100030000008
0.2953099910000034	0.7634400130000003	0.4585100110000013
0.0030000000000001	0.8343300220000032	0.0000000000000000
0.1989399939999998	0.8996499779999994	0.1111199999999997
0.3948799969999968	0.9649599789999996	0.2222500000000025
0.1938599940000003	0.8979499940000011	0.2500300109999998
0.3947600130000026	0.9629499910000021	0.3637000020000016
0.3019900019999966	0.7673299909999969	0.0277799999999999
0.4979299900000029	0.8326399919999972	0.1388999970000029
0.0858400019999976	0.6878200170000000	0.4962199929999969
0.6305199860000030	0.7101699709999991	0.1664600069999977
0.8264499900000004	0.7754799719999994	0.2775900070000006
0.9345800279999992	0.9781900050000019	0.0553399989999974
0.5293200019999986	0.8432400229999999	0.3982400000000013
0.7281600239999975	0.9091899989999987	0.5350099799999981
0.5274699929999969	0.8424900170000029	0.2498099949999997
0.7275300029999983	0.9097899790000028	0.3632099929999981
0.9229699970000027	0.9737499949999986	0.4751099940000003
0.6355999709999978	0.7118700150000024	0.0275599989999975
0.8315299750000023	0.7771800160000026	0.1386799960000005
0.5910500290000016	0.6973900200000003	0.3371599910000000
0.7851799729999982	0.7644600269999984	0.4493600130000033
0.5030000209999983	0.8343300220000032	0.0000000000000000

0.6989399790000022	0.8996499779999994	0.1111199999999997
0.8948799969999968	0.9649599789999996	0.2222500000000025
0.6938599940000003	0.8979499940000011	0.2500300109999998
0.8982899789999976	0.9644500019999995	0.3621599969999991
0.8019899729999977	0.7673299909999969	0.0277799999999999
0.9979299900000029	0.8326399919999972	0.1388999970000029
0.6016399860000021	0.7026500110000029	0.4812200069999975
0.0182389114019570	0.5144173485327890	0.7296860010464475
0.1442812562641082	0.7107312820441770	0.6194243262725715
0.0490094165896048	0.5211857203301048	0.6129153268804274
0.1960200070000013	0.7266899939999973	0.5159500239999986
0.1835477680348480	0.5704305469507391	0.7317870420157175
0.0962314591509826	0.6033499386518388	0.7123483875105084
0.0970775032761936	0.6158114689693173	0.6430355403673861
0.1854696115728627	0.5656272477272839	0.7820158899975915
0.2423117088372242	0.6402024403630566	0.7163636726558366
0.1859706575685136	0.4814028112096693	0.7129223527250671
0.0960970537300309	0.6943249320515754	0.7306401127285923
0.0133733055156784	0.5150650512217170	0.7742446840835887
0.0545968107388090	0.5328835573346523	0.5681602302923522

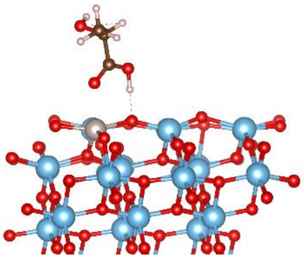

Supplementary Fig. 36b

Ti	O	Ru	C	H
53	123	1	3	6

0.2162500020000024	0.0720800010000033	0.0971200019999969
0.4121899900000017	0.1374000010000032	0.2082500010000032
0.1075700000000026	0.2017599939999997	0.3191800119999968
0.3223899899999978	0.2694700059999988	0.4248200059999974
0.1132100000000023	0.2044000030000035	0.1804700049999965
0.3091399970000026	0.2697100039999967	0.2915900050000033
0.4172700049999989	0.1390900020000032	0.0693399979999967
0.0111699999999999	0.0034199999999984	0.4150600139999980
0.2061800059999968	0.0639299970000025	0.5169799920000031
0.7162500020000024	0.0720800010000033	0.0971200019999969
0.9121900199999970	0.1374000010000032	0.2082500010000032
0.6080600020000020	0.2023700029999986	0.3199799949999971
0.8129900100000000	0.2696099879999991	0.4331600069999979
0.6132100219999970	0.2044000030000035	0.1804700049999965

0.8091400269999980	0.2697100039999967	0.2915900050000033
0.9172700049999989	0.1390900020000032	0.0693399979999967
0.5113999840000005	0.0040400000000034	0.4151499869999995
0.7064099910000010	0.0657000019999998	0.5152699949999970
0.2162500020000024	0.4054200050000034	0.0971200019999969
0.4121899900000017	0.4707300070000002	0.2082500010000032
0.1083699989999971	0.5364099740000015	0.3192299899999966
0.3214699919999973	0.6141999959999964	0.4265199900000027
0.1132100000000023	0.5377399919999988	0.1804700049999965
0.3091399970000026	0.6030499940000027	0.2915900050000033
0.4172700049999989	0.4724200069999966	0.0693399979999967
0.0018999999999991	0.3338199849999981	0.4078600109999968
0.7162500020000024	0.4054200050000034	0.0971200019999969
0.9121900199999970	0.4707300070000002	0.2082500010000032
0.6079300050000001	0.5363500119999998	0.3196099999999973
0.8137900230000028	0.6065099839999988	0.4318799970000029
0.6132100219999970	0.5377399919999988	0.1804700049999965
0.8091400269999980	0.6030499940000027	0.2915900050000033
0.9172700049999989	0.4724200069999966	0.0693399979999967
0.5153499839999967	0.3389999870000011	0.4159300029999997
0.7077400089999983	0.4033299979999967	0.5147299770000018
0.2162500020000024	0.7387499809999980	0.0971200019999969
0.4121899900000017	0.8040599819999983	0.2082500010000032
0.1092500020000031	0.8700299859999987	0.3198699949999977
0.3179000020000018	0.9390599729999991	0.4311099949999999
0.1132100000000023	0.8710700270000018	0.1804700049999965
0.3091399970000026	0.9363800289999986	0.2915900050000033
0.4172700049999989	0.8057600260000015	0.0693399979999967
0.0036199999999980	0.6676599979999978	0.4101600050000016
0.7162500020000024	0.7387499809999980	0.0971200019999969
0.9121900199999970	0.8040599819999983	0.2082500010000032
0.6075699929999985	0.8695999979999982	0.3198499980000022
0.8191800119999968	0.9394699930000030	0.4279299969999997
0.6132100219999970	0.8710700270000018	0.1804700049999965
0.8091400269999980	0.9363800289999986	0.2915900050000033
0.9172700049999989	0.8057600260000015	0.0693399979999967
0.5146399739999978	0.6710900069999965	0.4159600139999995
0.7072200180000010	0.7384600039999967	0.5159699920000023
0.2046100049999993	0.3986899849999972	0.5146800280000008
0.1305200010000007	0.0435100010000014	0.1664600069999977
0.3264499900000004	0.1088199989999978	0.2775900070000006
0.4345799979999967	0.3115299939999971	0.0553399989999974
0.0293400010000013	0.1772899930000023	0.3975299889999988
0.2262199970000012	0.2542400060000034	0.5435600280000017

0.0274700000000010	0.1758199929999975	0.2498099949999997
0.2267699989999983	0.2455500069999985	0.3635900020000022
0.4213500019999969	0.3162199849999965	0.4740999939999995
0.1356000010000002	0.0452000009999978	0.0275599989999975
0.3315300049999976	0.1105099990000014	0.1386799960000005
0.0905399990000006	0.0298299990000004	0.3367600139999993
0.2873699960000025	0.0996899980000023	0.4522100089999981
0.0030000000000001	0.1676699969999973	0.0000000000000000
0.1989399939999998	0.2329799979999976	0.1111199999999997
0.3948799969999968	0.2982900140000027	0.2222500000000025
0.1938599940000003	0.2312899980000012	0.2500300109999998
0.3991700110000025	0.2940100129999976	0.3600299949999979
0.3019900019999966	0.1006600039999981	0.0277799999999999
0.4979299900000029	0.1659799959999972	0.1388999970000029
0.1032600030000026	0.0440200009999998	0.4816800059999977
0.6305199860000030	0.0435100010000014	0.1664600069999977
0.8264499900000004	0.1088199989999978	0.2775900070000006
0.9345800279999992	0.3115299939999971	0.0553399989999974
0.5301700229999966	0.1771599949999967	0.3982200029999987
0.7334100010000029	0.2441799939999996	0.5260000230000017
0.5274699929999969	0.1758199929999975	0.2498099949999997
0.7273899910000026	0.2405399980000027	0.3637399970000033
0.9212499859999994	0.3076300020000033	0.4740799960000004
0.6355999709999978	0.0452000009999978	0.0275599989999975
0.8315299750000023	0.1105099990000014	0.1386799960000005
0.5897600050000023	0.0300099999999972	0.3368000090000010
0.7858499880000025	0.0922100020000016	0.4492900070000019
0.5030000209999983	0.1676699969999973	0.0000000000000000
0.6989399790000022	0.2329799979999976	0.1111199999999997
0.8948799969999968	0.2982900140000027	0.2222500000000025
0.6938599940000003	0.2312899980000012	0.2500300109999998
0.8935300110000028	0.2989200060000030	0.3628000020000002
0.8019899729999977	0.1006600039999981	0.0277799999999999
0.9979299900000029	0.1659799959999972	0.1388999970000029
0.6015099879999966	0.0313599999999994	0.4803699850000029
0.1305200010000007	0.3768399949999974	0.1664600069999977
0.3264499900000004	0.4421499970000013	0.2775900070000006
0.4345799979999967	0.6448600290000002	0.0553399989999974
0.0327900010000022	0.5111200209999964	0.4001100059999985
0.2221799940000011	0.5642099980000026	0.5350300070000031
0.0274700000000010	0.5091599819999999	0.2498099949999997
0.2267799970000013	0.5746200079999966	0.3642700019999978
0.4218499959999988	0.6262800100000021	0.4741500020000018
0.1356000010000002	0.3785299959999975	0.0275599989999975

0.3315300049999976	0.4438399969999978	0.1386799960000005
0.0892499979999997	0.3636200130000020	0.3357999919999983
0.2833000119999980	0.4265899959999970	0.4493899940000006
0.0030000000000001	0.5009999870000001	0.0000000000000000
0.1989399939999998	0.5663099880000004	0.1111199999999997
0.3948799969999968	0.6316300029999979	0.2222500000000025
0.1938599940000003	0.5646200179999994	0.2500300109999998
0.3977900149999982	0.6409800050000030	0.3610199989999998
0.3019900019999966	0.4339999849999998	0.0277799999999999
0.4979299900000029	0.4993099869999966	0.1388999970000029
0.0989599970000015	0.3597100080000004	0.4823699889999986
0.6305199860000030	0.3768399949999974	0.1664600069999977
0.8264499900000004	0.4421499970000013	0.2775900070000006
0.9345800279999992	0.6448600290000002	0.0553399989999974
0.5294799799999979	0.5098099710000028	0.3965699970000003
0.7324299810000028	0.5783500080000010	0.5280699730000009
0.5274699929999969	0.5091599819999999	0.2498099949999997
0.7275300029999983	0.5776100160000013	0.3634499910000031
0.9201700090000031	0.6401600239999965	0.4747500120000012
0.6355999709999978	0.3785299959999975	0.0275599989999975
0.8315299750000023	0.4438399969999978	0.1386799960000005
0.5921099780000034	0.3641299900000021	0.3374899920000018
0.7838699819999988	0.4287199970000017	0.4476999939999970
0.5030000209999983	0.5009999870000001	0.0000000000000000
0.6989399790000022	0.5663099880000004	0.1111199999999997
0.8948799969999968	0.6316300029999979	0.2222500000000025
0.6938599940000003	0.5646200179999994	0.2500300109999998
0.8951299790000036	0.6326900119999976	0.3632099929999981
0.8019899729999977	0.4339999849999998	0.0277799999999999
0.9979299900000029	0.4993099869999966	0.1388999970000029
0.6007800100000011	0.3676500019999978	0.4821299910000008
0.1305200010000007	0.7101699709999991	0.1664600069999977
0.3264499900000004	0.7754799719999994	0.2775900070000006
0.4345799979999967	0.9781900050000019	0.0553399989999974
0.0343700009999992	0.8433200119999995	0.4014999870000011
0.2219299969999966	0.9049199820000027	0.5234400029999975
0.0274700000000010	0.8424900170000029	0.2498099949999997
0.2264199999999974	0.9067500230000007	0.3641999960000035
0.4218600089999995	0.9805999989999989	0.4759800140000010
0.1356000010000002	0.7118700150000024	0.0275599989999975
0.3315300049999976	0.7771800160000026	0.1386799960000005
0.0891700009999994	0.6960999969999975	0.3365100030000008
0.2953099910000034	0.7634400130000003	0.4585100110000013
0.0030000000000001	0.8343300220000032	0.0000000000000000

0.1989399939999998	0.8996499779999994	0.1111199999999997
0.3948799969999968	0.9649599789999996	0.2222500000000025
0.1938599940000003	0.8979499940000011	0.2500300109999998
0.3947600130000026	0.9629499910000021	0.3637000020000016
0.3019900019999966	0.7673299909999969	0.0277799999999999
0.4979299900000029	0.8326399919999972	0.1388999970000029
0.0858400019999976	0.6878200170000000	0.4962199929999969
0.6305199860000030	0.7101699709999991	0.1664600069999977
0.8264499900000004	0.7754799719999994	0.2775900070000006
0.9345800279999992	0.9781900050000019	0.0553399989999974
0.5293200019999986	0.8432400229999999	0.3982400000000013
0.7281600239999975	0.9091899989999987	0.5350099799999981
0.5274699929999969	0.8424900170000029	0.2498099949999997
0.7275300029999983	0.9097899790000028	0.3632099929999981
0.9229699970000027	0.9737499949999986	0.4751099940000003
0.6355999709999978	0.7118700150000024	0.0275599989999975
0.8315299750000023	0.7771800160000026	0.1386799960000005
0.5910500290000016	0.6973900200000003	0.3371599910000000
0.7851799729999982	0.7644600269999984	0.4493600130000033
0.5030000209999983	0.8343300220000032	0.0000000000000000
0.6989399790000022	0.8996499779999994	0.1111199999999997
0.8948799969999968	0.9649599789999996	0.2222500000000025
0.6938599940000003	0.8979499940000011	0.2500300109999998
0.8982899789999976	0.9644500019999995	0.3621599969999991
0.8019899729999977	0.7673299909999969	0.0277799999999999
0.9979299900000029	0.8326399919999972	0.1388999970000029
0.6016399860000021	0.7026500110000029	0.4812200069999975
0.2154512770771450	0.7934785471379497	0.7478769859541177
0.1479325766340681	0.7311912405828169	0.6199864233323289
0.2112062498816930	0.5813877188146712	0.6524937540744659
0.1960200070000013	0.7266899939999973	0.5159500239999986
0.0559179841428351	0.7106875497317170	0.7380915810757406
0.1481959584936008	0.6885158586070650	0.7293257754172421
0.1686335563161608	0.6666250598676910	0.6616262542601425
0.0448236144048600	0.7230661238069520	0.7870935205347684
0.0039811936737740	0.6323225264838360	0.7215982788339108
0.0500217106250502	0.7924129961737917	0.7131057440826739
0.1548954438251259	0.6069266419865459	0.7545574320965859
0.2640964575712285	0.7673436556854665	0.7680348338611082
0.2211737399468074	0.5736074239846699	0.6057633164894479

Supplementary Fig. 36c

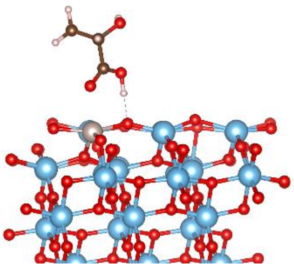

Ti	O	Ru	C	H
53	123	1	3	6

0.2162500020000024	0.0720800010000033	0.0971200019999969
0.4121899900000017	0.1374000010000032	0.2082500010000032
0.1075700000000026	0.2017599939999997	0.3191800119999968
0.3223899899999978	0.2694700059999988	0.4248200059999974
0.1132100000000023	0.2044000030000035	0.1804700049999965
0.3091399970000026	0.2697100039999967	0.2915900050000033
0.4172700049999989	0.1390900020000032	0.0693399979999967
0.0111699999999999	0.0034199999999984	0.4150600139999980
0.2061800059999968	0.0639299970000025	0.5169799920000031
0.7162500020000024	0.0720800010000033	0.0971200019999969
0.9121900199999970	0.1374000010000032	0.2082500010000032
0.6080600020000020	0.2023700029999986	0.3199799949999971
0.8129900100000000	0.2696099879999991	0.4331600069999979
0.6132100219999970	0.2044000030000035	0.1804700049999965
0.8091400269999980	0.2697100039999967	0.2915900050000033
0.9172700049999989	0.1390900020000032	0.0693399979999967
0.5113999840000005	0.0040400000000034	0.4151499869999995
0.7064099910000010	0.0657000019999998	0.5152699949999970
0.2162500020000024	0.4054200050000034	0.0971200019999969
0.4121899900000017	0.4707300070000002	0.2082500010000032
0.1083699989999971	0.5364099740000015	0.3192299899999966
0.3214699919999973	0.6141999959999964	0.4265199900000027
0.1132100000000023	0.5377399919999988	0.1804700049999965
0.3091399970000026	0.6030499940000027	0.2915900050000033
0.4172700049999989	0.4724200069999966	0.0693399979999967
0.0018999999999991	0.3338199849999981	0.4078600109999968
0.7162500020000024	0.4054200050000034	0.0971200019999969
0.9121900199999970	0.4707300070000002	0.2082500010000032
0.6079300050000001	0.5363500119999998	0.3196099999999973
0.8137900230000028	0.6065099839999988	0.4318799970000029
0.6132100219999970	0.5377399919999988	0.1804700049999965
0.8091400269999980	0.6030499940000027	0.2915900050000033
0.9172700049999989	0.4724200069999966	0.0693399979999967
0.5153499839999967	0.3389999870000011	0.4159300029999997
0.7077400089999983	0.4033299979999967	0.5147299770000018

0.2162500020000024	0.7387499809999980	0.0971200019999969
0.4121899900000017	0.8040599819999983	0.2082500010000032
0.1092500020000031	0.8700299859999987	0.3198699949999977
0.3179000020000018	0.9390599729999991	0.4311099949999999
0.1132100000000023	0.8710700270000018	0.1804700049999965
0.3091399970000026	0.9363800289999986	0.2915900050000033
0.4172700049999989	0.8057600260000015	0.0693399979999967
0.0036199999999980	0.6676599979999978	0.4101600050000016
0.7162500020000024	0.7387499809999980	0.0971200019999969
0.9121900199999970	0.8040599819999983	0.2082500010000032
0.6075699929999985	0.8695999979999982	0.3198499980000022
0.8191800119999968	0.9394699930000030	0.4279299969999997
0.6132100219999970	0.8710700270000018	0.1804700049999965
0.8091400269999980	0.9363800289999986	0.2915900050000033
0.9172700049999989	0.8057600260000015	0.0693399979999967
0.5146399739999978	0.6710900069999965	0.4159600139999995
0.7072200180000010	0.7384600039999967	0.5159699920000023
0.2046100049999993	0.3986899849999972	0.5146800280000008
0.1305200010000007	0.0435100010000014	0.1664600069999977
0.3264499900000004	0.1088199989999978	0.2775900070000006
0.4345799979999967	0.3115299939999971	0.0553399989999974
0.0293400010000013	0.1772899930000023	0.3975299889999988
0.2262199970000012	0.2542400060000034	0.5435600280000017
0.0274700000000010	0.1758199929999975	0.2498099949999997
0.2267699989999983	0.2455500069999985	0.3635900020000022
0.4213500019999969	0.3162199849999965	0.4740999939999995
0.1356000010000002	0.0452000009999978	0.0275599989999975
0.3315300049999976	0.1105099990000014	0.1386799960000005
0.0905399990000006	0.0298299990000004	0.3367600139999993
0.2873699960000025	0.0996899980000023	0.4522100089999981
0.0030000000000001	0.1676699969999973	0.0000000000000000
0.1989399939999998	0.2329799979999976	0.1111199999999997
0.3948799969999968	0.2982900140000027	0.2222500000000025
0.1938599940000003	0.2312899980000012	0.2500300109999998
0.3991700110000025	0.2940100129999976	0.3600299949999979
0.3019900019999966	0.1006600039999981	0.0277799999999999
0.4979299900000029	0.1659799959999972	0.1388999970000029
0.1032600030000026	0.0440200009999998	0.4816800059999977
0.6305199860000030	0.0435100010000014	0.1664600069999977
0.8264499900000004	0.1088199989999978	0.2775900070000006
0.9345800279999992	0.3115299939999971	0.0553399989999974
0.5301700229999966	0.1771599949999967	0.3982200029999987
0.7334100010000029	0.2441799939999996	0.5260000230000017
0.5274699929999969	0.1758199929999975	0.2498099949999997

0.7273899910000026	0.2405399980000027	0.3637399970000033
0.9212499859999994	0.3076300020000033	0.4740799960000004
0.6355999709999978	0.0452000009999978	0.0275599989999975
0.8315299750000023	0.1105099990000014	0.1386799960000005
0.5897600050000023	0.0300099999999972	0.3368000090000010
0.7858499880000025	0.0922100020000016	0.4492900070000019
0.5030000209999983	0.1676699969999973	0.0000000000000000
0.6989399790000022	0.2329799979999976	0.1111199999999997
0.8948799969999968	0.2982900140000027	0.2222500000000025
0.6938599940000003	0.2312899980000012	0.2500300109999998
0.8935300110000028	0.2989200060000030	0.3628000020000002
0.8019899729999977	0.1006600039999981	0.0277799999999999
0.9979299900000029	0.1659799959999972	0.1388999970000029
0.6015099879999966	0.0313599999999994	0.4803699850000029
0.1305200010000007	0.3768399949999974	0.1664600069999977
0.3264499900000004	0.4421499970000013	0.2775900070000006
0.4345799979999967	0.6448600290000002	0.0553399989999974
0.0327900010000022	0.5111200209999964	0.4001100059999985
0.2221799940000011	0.5642099980000026	0.5350300070000031
0.0274700000000010	0.5091599819999999	0.2498099949999997
0.2267799970000013	0.5746200079999966	0.3642700019999978
0.4218499959999988	0.6262800100000021	0.4741500020000018
0.1356000010000002	0.3785299959999975	0.0275599989999975
0.3315300049999976	0.4438399969999978	0.1386799960000005
0.0892499979999997	0.3636200130000020	0.3357999919999983
0.2833000119999980	0.4265899959999970	0.4493899940000006
0.0030000000000001	0.5009999870000001	0.0000000000000000
0.1989399939999998	0.5663099880000004	0.1111199999999997
0.3948799969999968	0.6316300029999979	0.2222500000000025
0.1938599940000003	0.5646200179999994	0.2500300109999998
0.3977900149999982	0.6409800050000030	0.3610199989999998
0.3019900019999966	0.4339999849999998	0.0277799999999999
0.4979299900000029	0.4993099869999966	0.1388999970000029
0.0989599970000015	0.3597100080000004	0.4823699889999986
0.6305199860000030	0.3768399949999974	0.1664600069999977
0.8264499900000004	0.4421499970000013	0.2775900070000006
0.9345800279999992	0.6448600290000002	0.0553399989999974
0.5294799799999979	0.5098099710000028	0.3965699970000003
0.7324299810000028	0.5783500080000010	0.5280699730000009
0.5274699929999969	0.5091599819999999	0.2498099949999997
0.7275300029999983	0.5776100160000013	0.3634499910000031
0.9201700090000031	0.6401600239999965	0.4747500120000012
0.6355999709999978	0.3785299959999975	0.0275599989999975
0.8315299750000023	0.4438399969999978	0.1386799960000005

0.5921099780000034	0.3641299900000021	0.3374899920000018
0.7838699819999988	0.4287199970000017	0.4476999939999970
0.5030000209999983	0.5009999870000001	0.0000000000000000
0.6989399790000022	0.5663099880000004	0.1111199999999997
0.8948799969999968	0.6316300029999979	0.2222500000000025
0.6938599940000003	0.5646200179999994	0.2500300109999998
0.8951299790000036	0.6326900119999976	0.3632099929999981
0.8019899729999977	0.4339999849999998	0.0277799999999999
0.9979299900000029	0.4993099869999966	0.1388999970000029
0.6007800100000011	0.3676500019999978	0.4821299910000008
0.1305200010000007	0.7101699709999991	0.1664600069999977
0.3264499900000004	0.7754799719999994	0.2775900070000006
0.4345799979999967	0.9781900050000019	0.0553399989999974
0.0343700009999992	0.8433200119999995	0.4014999870000011
0.2219299969999966	0.9049199820000027	0.5234400029999975
0.0274700000000010	0.8424900170000029	0.2498099949999997
0.2264199999999974	0.9067500230000007	0.3641999960000035
0.4218600089999995	0.9805999989999989	0.4759800140000010
0.1356000010000002	0.7118700150000024	0.0275599989999975
0.3315300049999976	0.7771800160000026	0.1386799960000005
0.0891700009999994	0.6960999969999975	0.3365100030000008
0.2953099910000034	0.7634400130000003	0.4585100110000013
0.0030000000000001	0.8343300220000032	0.0000000000000000
0.1989399939999998	0.8996499779999994	0.1111199999999997
0.3948799969999968	0.9649599789999996	0.2222500000000025
0.1938599940000003	0.8979499940000011	0.2500300109999998
0.3947600130000026	0.9629499910000021	0.3637000020000016
0.3019900019999966	0.7673299909999969	0.0277799999999999
0.4979299900000029	0.8326399919999972	0.1388999970000029
0.0858400019999976	0.6878200170000000	0.4962199929999969
0.6305199860000030	0.7101699709999991	0.1664600069999977
0.8264499900000004	0.7754799719999994	0.2775900070000006
0.9345800279999992	0.9781900050000019	0.0553399989999974
0.5293200019999986	0.8432400229999999	0.3982400000000013
0.7281600239999975	0.9091899989999987	0.5350099799999981
0.5274699929999969	0.8424900170000029	0.2498099949999997
0.7275300029999983	0.9097899790000028	0.3632099929999981
0.9229699970000027	0.9737499949999986	0.4751099940000003
0.6355999709999978	0.7118700150000024	0.0275599989999975
0.8315299750000023	0.7771800160000026	0.1386799960000005
0.5910500290000016	0.6973900200000003	0.3371599910000000
0.7851799729999982	0.7644600269999984	0.4493600130000033
0.5030000209999983	0.8343300220000032	0.0000000000000000
0.6989399790000022	0.8996499779999994	0.1111199999999997

0.8948799969999968	0.9649599789999996	0.2222500000000025
0.6938599940000003	0.8979499940000011	0.2500300109999998
0.8982899789999976	0.9644500019999995	0.3621599969999991
0.8019899729999977	0.7673299909999969	0.0277799999999999
0.9979299900000029	0.8326399919999972	0.1388899970000029
0.6016399860000021	0.7026500110000029	0.4812200069999975
0.1719048979396522	0.5997141738956592	0.7675537927582897
0.1578211637027883	0.7358570371422788	0.6218375087368230
0.2305520661875472	0.5947240858112296	0.6522086835781873
0.1960200070000013	0.7266899939999973	0.5159500239999986
0.2250558957758350	0.8212795093472353	0.7508427774332549
0.1651554375458758	0.6984170644880756	0.7304151991664398
0.1849122875723285	0.6755701480105952	0.6628180029100961
0.2098593067263193	0.8368262568765340	0.7990012610689553
0.2129028331402407	0.8958416851412102	0.7224273469946333
0.2961679951913885	0.8206585041072736	0.7471626422999993
0.0944463113558073	0.7015239404360779	0.7315741592217243
0.2340559765741780	0.6062107105347284	0.7792640022184278
0.2335510563729362	0.5820983517912420	0.6050568441388183

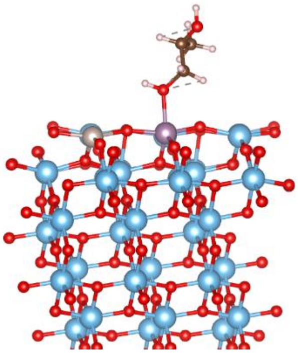

Supplementary Fig. 40a

Ti	O	Mo	Ru	C	H
52	122	1	1	3	8

0.2162500020000024	0.0720800010000033	0.0971200019999969
0.4121899900000017	0.1374000010000032	0.2082500010000032
0.1087316738803690	0.2025000578209675	0.3186470103089363
0.3226488345489658	0.2726255500877841	0.4250309194305790
0.1132100000000023	0.2044000030000035	0.1804700049999965
0.3091399970000026	0.2697100039999967	0.2915900050000033
0.4172700049999989	0.1390900020000032	0.0693399979999967
0.0117596095382155	0.0038908929711291	0.4125963707274865
0.2093507786221963	0.0659934420775792	0.5096354869553806
0.7162500020000024	0.0720800010000033	0.0971200019999969
0.9121900199999970	0.1374000010000032	0.2082500010000032
0.6085114839590372	0.2025605488315657	0.3189843940552503
0.8154790474056102	0.2710172362001745	0.4311409247871857

0.6132100219999970	0.2044000030000035	0.1804700049999965
0.8091400269999980	0.2697100039999967	0.2915900050000033
0.9172700049999989	0.1390900020000032	0.0693399979999967
0.5128296813213872	0.0044196142395368	0.4125747200273406
0.7084989442253703	0.0671970711469129	0.5115787722847883
0.2162500020000024	0.4054200050000034	0.0971200019999969
0.4121899900000017	0.4707300070000002	0.2082500010000032
0.1093201152152007	0.5365072143810112	0.3186310543164009
0.3222534393303053	0.6092958405283136	0.4262083956630162
0.1132100000000023	0.5377399919999988	0.1804700049999965
0.3091399970000026	0.6030499940000027	0.2915900050000033
0.4172700049999989	0.4724200069999966	0.0693399979999967
0.0036113097994062	0.3347919624059425	0.4065461873832785
0.7162500020000024	0.4054200050000034	0.0971200019999969
0.9121900199999970	0.4707300070000002	0.2082500010000032
0.6084567277235577	0.5362041165214111	0.3188528172168040
0.8147674808389016	0.6065234731032391	0.4306829364702491
0.6132100219999970	0.5377399919999988	0.1804700049999965
0.8091400269999980	0.6030499940000027	0.2915900050000033
0.9172700049999989	0.4724200069999966	0.0693399979999967
0.5144734291462608	0.3381853266015508	0.4130804408836202
0.7084992307267663	0.4031975472632287	0.5108027333952128
0.2162500020000024	0.7387499809999980	0.0971200019999969
0.4121899900000017	0.8040599819999983	0.2082500010000032
0.1095176859924420	0.8700941549259481	0.3189611688391060
0.3210382579285957	0.9398191497528056	0.4280479720492371
0.1132100000000023	0.8710700270000018	0.1804700049999965
0.3091399970000026	0.9363800289999986	0.2915900050000033
0.4172700049999989	0.8057600260000015	0.0693399979999967
0.0037603447561476	0.6677714824830471	0.4073745133345515
0.7162500020000024	0.7387499809999980	0.0971200019999969
0.9121900199999970	0.8040599819999983	0.2082500010000032
0.6081854526682035	0.8695302564750880	0.3190168187468437
0.8207954425342187	0.9406051511768354	0.4265661861300764
0.6132100219999970	0.8710700270000018	0.1804700049999965
0.8091400269999980	0.9363800289999986	0.2915900050000033
0.9172700049999989	0.8057600260000015	0.0693399979999967
0.5143476069085871	0.6708603619624277	0.4130963571163991
0.7086632613123727	0.7394249747015643	0.5118149023547591
0.1305200010000007	0.0435100010000014	0.1664600069999977
0.3264499900000004	0.1088199989999978	0.2775900070000006
0.4345799979999967	0.3115299939999971	0.0553399989999974
0.0310219674097903	0.1784923643943045	0.3971992445882864
0.2303090780583414	0.2454328533977984	0.5304858984862720

0.0274700000000010	0.1758199929999975	0.2498099949999997
0.2270907691094361	0.2470247158786033	0.3630505943413034
0.4231063652486825	0.3119046267117087	0.4732194628992644
0.1356000010000002	0.0452000009999978	0.0275599989999975
0.3315300049999976	0.1105099990000014	0.1386799960000005
0.0924198231397878	0.0307098649986656	0.3357480023392693
0.2891874472770813	0.0986718052224086	0.4446711856910356
0.0030000000000001	0.1676699969999973	0.0000000000000000
0.1989399939999998	0.2329799979999976	0.1111199999999997
0.3948799969999968	0.2982900140000027	0.2222500000000025
0.1938599940000003	0.2312899980000012	0.2500300109999998
0.3993140088252136	0.2982267547794996	0.3593935709859101
0.3019900019999966	0.1006600039999981	0.0277799999999999
0.4979299900000029	0.1659799959999972	0.1388999970000029
0.1036167337324320	0.0387623356539002	0.4779425805220410
0.6305199860000030	0.0435100010000014	0.1664600069999977
0.8264499900000004	0.1088199989999978	0.2775900070000006
0.9345800279999992	0.3115299939999971	0.0553399989999974
0.5294753719322393	0.1768868037595504	0.3957067520844241
0.7352705536845801	0.2450696556637706	0.5230057059777746
0.5274699929999969	0.1758199929999975	0.2498099949999997
0.7283761362848533	0.2409065273607375	0.3633518408783546
0.9234071156504707	0.3097330872567191	0.4728413732936354
0.6355999709999978	0.0452000009999978	0.0275599989999975
0.8315299750000023	0.1105099990000014	0.1386799960000005
0.5921453735620923	0.0306500765636459	0.3355756614037231
0.7887041279141414	0.0943112612310110	0.4464227909259900
0.5030000209999983	0.1676699969999973	0.0000000000000000
0.6989399790000022	0.2329799979999976	0.1111199999999997
0.8948799969999968	0.2982900140000027	0.2222500000000025
0.6938599940000003	0.2312899980000012	0.2500300109999998
0.8959814723099229	0.2998649441454201	0.3611896807134349
0.8019899729999977	0.1006600039999981	0.0277799999999999
0.9979299900000029	0.1659799959999972	0.1388999970000029
0.6037316746944107	0.0330722491782112	0.4768529847885291
0.1305200010000007	0.3768399949999974	0.1664600069999977
0.3264499900000004	0.4421499970000013	0.2775900070000006
0.4345799979999967	0.6448600290000002	0.0553399989999974
0.0348627913166675	0.5118275238995622	0.3999740670781671
0.2237071164978145	0.5744655850707850	0.5221520350458305
0.0274700000000010	0.5091599819999999	0.2498099949999997
0.2273420141525963	0.5746708848444473	0.3639255499919711
0.4235619734688388	0.6284864690793500	0.4732797969676766
0.1356000010000002	0.3785299959999975	0.0275599989999975

0.3315300049999976	0.4438399969999978	0.1386799960000005
0.0904311969925735	0.3642565648677217	0.3349181457953932
0.2853069732071052	0.4266951354060454	0.4495812273277037
0.0030000000000001	0.5009999870000001	0.0000000000000000
0.1989399939999998	0.5663099880000004	0.1111199999999997
0.3948799969999968	0.6316300029999979	0.2222500000000025
0.1938599940000003	0.5646200179999994	0.2500300109999998
0.3984468109050020	0.6368929592969337	0.3600811531205779
0.3019900019999966	0.4339999849999998	0.0277799999999999
0.4979299900000029	0.4993099869999966	0.1388999970000029
0.1002646560674065	0.3608724513638118	0.4846999617025497
0.6305199860000030	0.3768399949999974	0.1664600069999977
0.8264499900000004	0.4421499970000013	0.2775900070000006
0.9345800279999992	0.6448600290000002	0.0553399989999974
0.5295185804080416	0.5095094684195889	0.3953012754611862
0.7343534325555292	0.5790349814429657	0.5231920443902758
0.5274699929999969	0.5091599819999999	0.2498099949999997
0.7277545055500180	0.5778186496876008	0.3631165963392889
0.9214395661439693	0.6391183387314642	0.4729916058270298
0.6355999709999978	0.3785299959999975	0.0275599989999975
0.8315299750000023	0.4438399969999978	0.1386799960000005
0.5937101758814076	0.3644985433976451	0.3360843732518353
0.7853054599384438	0.4286838273253866	0.4443610422158700
0.5030000209999983	0.5009999870000001	0.0000000000000000
0.6989399790000022	0.5663099880000004	0.1111199999999997
0.8948799969999968	0.6316300029999979	0.2222500000000025
0.6938599940000003	0.5646200179999994	0.2500300109999998
0.8958063417187786	0.6317285578246161	0.3613417813668859
0.8019899729999977	0.4339999849999998	0.0277799999999999
0.9979299900000029	0.4993099869999966	0.1388999970000029
0.6016617990783433	0.3670438679569751	0.4788803927161134
0.1305200010000007	0.7101699709999991	0.1664600069999977
0.3264499900000004	0.7754799719999994	0.2775900070000006
0.4345799979999967	0.9781900050000019	0.0553399989999974
0.0327496008764982	0.8428026664146477	0.3986440377249013
0.2288420359895610	0.9071676628565438	0.5183341028137614
0.0274700000000010	0.8424900170000029	0.2498099949999997
0.2270257357813123	0.9046863531882759	0.3635611451705172
0.4241597705474059	0.9811504532214599	0.4738470870709410
0.1356000010000002	0.7118700150000024	0.0275599989999975
0.3315300049999976	0.7771800160000026	0.1386799960000005
0.0904620407760525	0.6961904675635514	0.3354234293013260
0.2987179956810173	0.7643968630138085	0.4539105740158761
0.0030000000000001	0.8343300220000032	0.0000000000000000

0.1989399939999998	0.8996499779999994	0.1111199999999997
0.3948799969999968	0.9649599789999996	0.2222500000000025
0.1938599940000003	0.8979499940000011	0.2500300109999998
0.3974534554333676	0.9638692732463431	0.3607976256311692
0.3019900019999966	0.7673299909999969	0.0277799999999999
0.4979299900000029	0.8326399919999972	0.1388999970000029
0.0865598668598019	0.6979627111227900	0.4914163651456866
0.6305199860000030	0.7101699709999991	0.1664600069999977
0.8264499900000004	0.7754799719999994	0.2775900070000006
0.9345800279999992	0.9781900050000019	0.0553399989999974
0.5286868144755753	0.8427124627515036	0.3961832218942327
0.7309016316600954	0.9106601876173898	0.5304700120901262
0.5274699929999969	0.8424900170000029	0.2498099949999997
0.7279057426207854	0.9099077903955897	0.3629774984971068
0.9243975676729521	0.9748739316061922	0.4735271991108029
0.6355999709999978	0.7118700150000024	0.0275599989999975
0.8315299750000023	0.7771800160000026	0.1386799960000005
0.5922953756939933	0.6975786099302663	0.3358782380357204
0.7877527755901343	0.7654288185852041	0.4462088371577956
0.5030000209999983	0.8343300220000032	0.0000000000000000
0.6989399790000022	0.8996499779999994	0.1111199999999997
0.8948799969999968	0.9649599789999996	0.2222500000000025
0.6938599940000003	0.8979499940000011	0.2500300109999998
0.8993331255436960	0.9658062631727083	0.3604268603516321
0.8019899729999977	0.7673299909999969	0.0277799999999999
0.9979299900000029	0.8326399919999972	0.1388999970000029
0.6031169434990220	0.7038088371805065	0.4777277309537011
0.1259578220551793	0.2579610417788235	0.7587204221370080
0.1950309503987574	0.4114205647285150	0.6146449662055837
0.2056131589215662	0.4005473893970420	0.5163295927442034
0.1972883167292719	0.7315674922817837	0.5079193752747071
0.2793003323863426	0.2868108186749844	0.7188435888764636
0.1931646068565902	0.3302057675275925	0.7202929217978773
0.1457580500313594	0.3193984847480393	0.6582436997355864
0.3127851361742494	0.3004364018973669	0.7636519040608737
0.3265867069466036	0.3371085849665299	0.6845549360154848
0.2639460138602298	0.1890510701157191	0.7079741525210425
0.2083406518174757	0.4273716742717820	0.7345147712589906
0.1465420474552114	0.2635555801854058	0.8011304198718950
0.0777647747653565	0.3325002789326945	0.6633090929297526
0.1429266466990427	0.2314485192329927	0.6363888724372132
0.1973234033914160	0.4935325931683339	0.6295444660211105

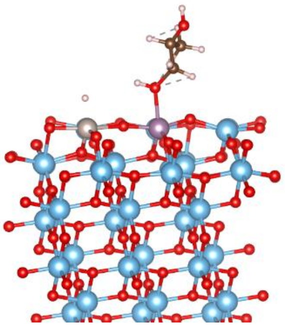

Supplementary Fig. 40b

Ti	O	Mo	Ru	C	H
52	122	1	1	3	9

0.2162500020000024	0.0720800010000033	0.0971200019999969
0.4121899900000017	0.1374000010000032	0.2082500010000032
0.1085391511729181	0.2022386724016830	0.3185196609510364
0.3256752354025058	0.2744940556810179	0.4232447746354812
0.1132100000000023	0.2044000030000035	0.1804700049999965
0.3091399970000026	0.2697100039999967	0.2915900050000033
0.4172700049999989	0.1390900020000032	0.0693399979999967
0.0109721392998493	0.0032894267580500	0.4118511342961653
0.2077696804723225	0.0716918559688727	0.5080572549630882
0.7162500020000024	0.0720800010000033	0.0971200019999969
0.9121900199999970	0.1374000010000032	0.2082500010000032
0.6087466364398194	0.2024751022676584	0.3190679166815928
0.8154212179822120	0.2712694835735100	0.4313892164746057
0.6132100219999970	0.2044000030000035	0.1804700049999965
0.8091400269999980	0.2697100039999967	0.2915900050000033
0.9172700049999989	0.1390900020000032	0.0693399979999967
0.5118034380534157	0.0041104512894773	0.4124035641968350
0.7080811848670929	0.0669843256202348	0.5110133397148720
0.2162500020000024	0.4054200050000034	0.0971200019999969
0.4121899900000017	0.4707300070000002	0.2082500010000032
0.1091430188891774	0.5370218768122662	0.3187906486079032
0.3215675667183621	0.6067694626698071	0.4269162714080356
0.1132100000000023	0.5377399919999988	0.1804700049999965
0.3091399970000026	0.6030499940000027	0.2915900050000033
0.4172700049999989	0.4724200069999966	0.0693399979999967
0.0032313560051639	0.3342777061548315	0.4055392481492912
0.7162500020000024	0.4054200050000034	0.0971200019999969
0.9121900199999970	0.4707300070000002	0.2082500010000032
0.6084347353458960	0.5365132785217857	0.3188306256789735
0.8168604640269086	0.6073997683103410	0.4297793753644367
0.6132100219999970	0.5377399919999988	0.1804700049999965
0.8091400269999980	0.6030499940000027	0.2915900050000033
0.9172700049999989	0.4724200069999966	0.0693399979999967
0.5167338691457430	0.3390836210781579	0.4133176370437993

0.7099000292768350	0.4045524364127219	0.5114328747355824
0.2162500020000024	0.7387499809999980	0.0971200019999969
0.4121899900000017	0.8040599819999983	0.2082500010000032
0.1095239282055867	0.8696787050862046	0.3190996705098183
0.3196011080633370	0.9434354449494525	0.4288832054651627
0.1132100000000023	0.8710700270000018	0.1804700049999965
0.3091399970000026	0.9363800289999986	0.2915900050000033
0.4172700049999989	0.8057600260000015	0.0693399979999967
0.0058794025230899	0.6683759187130748	0.4097237722200522
0.7162500020000024	0.7387499809999980	0.0971200019999969
0.9121900199999970	0.8040599819999983	0.2082500010000032
0.6083903860758761	0.8698077497521632	0.3190735525025443
0.8200977749732745	0.9395634978005662	0.4269574003970018
0.6132100219999970	0.8710700270000018	0.1804700049999965
0.8091400269999980	0.9363800289999986	0.2915900050000033
0.9172700049999989	0.8057600260000015	0.0693399979999967
0.5141487074016375	0.6711420092789111	0.4132175146259114
0.7084380245060080	0.7382370317125940	0.5119424252340034
0.1305200010000007	0.0435100010000014	0.1664600069999977
0.3264499900000004	0.1088199989999978	0.2775900070000006
0.4345799979999967	0.3115299939999971	0.0553399989999974
0.0262113739057028	0.1761658790341943	0.3933285247029845
0.2307152476827534	0.2458859090775741	0.5353358685808447
0.0274700000000010	0.1758199929999975	0.2498099949999997
0.2277604455120385	0.2476631608441997	0.3629228250077305
0.4248085525260023	0.3126290088189955	0.4727456087772544
0.1356000010000002	0.0452000009999978	0.0275599989999975
0.3315300049999976	0.1105099990000014	0.1386799960000005
0.0935806244964154	0.0305368663843855	0.3357056037595505
0.2898952798389676	0.1026215224931906	0.4437142696574836
0.0030000000000001	0.1676699969999973	0.0000000000000000
0.1989399939999998	0.2329799979999976	0.1111199999999997
0.3948799969999968	0.2982900140000027	0.2222500000000025
0.1938599940000003	0.2312899980000012	0.2500300109999998
0.4016915533147121	0.2997004890973456	0.3579845727138148
0.3019900019999966	0.1006600039999981	0.0277799999999999
0.4979299900000029	0.1659799959999972	0.1388899970000029
0.1021874978406068	0.0492720984907269	0.4761265898790344
0.6305199860000030	0.0435100010000014	0.1664600069999977
0.8264499900000004	0.1088199989999978	0.2775900070000006
0.9345800279999992	0.3115299939999971	0.0553399989999974
0.5298566624774868	0.1769662272752658	0.3958658378606584
0.7356535214049612	0.2451094060854149	0.5226132584811283
0.5274699929999969	0.1758199929999975	0.2498099949999997

0.7280288411786655	0.2411515518832551	0.3633842716707972
0.9235887961981122	0.3081739733402707	0.4724589307105984
0.6355999709999978	0.0452000009999978	0.0275599989999975
0.8315299750000023	0.1105099990000014	0.1386799960000005
0.5917247577352016	0.0304224186085910	0.3356164177049616
0.7881806993692050	0.0940175257875301	0.4458533511287694
0.5030000209999983	0.1676699969999973	0.0000000000000000
0.6989399790000022	0.2329799979999976	0.1111199999999997
0.8948799969999968	0.2982900140000027	0.2222500000000025
0.6938599940000003	0.2312899980000012	0.2500300109999998
0.8950525049445884	0.3018086555389088	0.3609386485653296
0.8019899729999977	0.1006600039999981	0.0277799999999999
0.9979299900000029	0.1659799959999972	0.1388999970000029
0.6029480447033294	0.0326869286375146	0.4767758333813659
0.1305200010000007	0.3768399949999974	0.1664600069999977
0.3264499900000004	0.4421499970000013	0.2775900070000006
0.4345799979999967	0.6448600290000002	0.0553399989999974
0.0343319215428490	0.5112713537424016	0.3996955638840818
0.2168672509135463	0.5637849909585216	0.5188286718481232
0.0274700000000010	0.5091599819999999	0.2498099949999997
0.2273347263368501	0.5763925851619037	0.3641713096590631
0.4228547643725710	0.6306993460723572	0.4738318910521490
0.1356000010000002	0.3785299959999975	0.0275599989999975
0.3315300049999976	0.4438399969999978	0.1386799960000005
0.0916959989257565	0.3649026942781429	0.3349684022736490
0.2866379277996014	0.4248128939143810	0.4490681071554254
0.0030000000000001	0.5009999870000001	0.0000000000000000
0.1989399939999998	0.5663099880000004	0.1111199999999997
0.3948799969999968	0.6316300029999979	0.2222500000000025
0.1938599940000003	0.5646200179999994	0.2500300109999998
0.3977608655026436	0.6351035003387479	0.3607114545359116
0.3019900019999966	0.4339999849999998	0.0277799999999999
0.4979299900000029	0.4993099869999966	0.1388999970000029
0.1003080330295467	0.3473405414047344	0.4792407812885679
0.6305199860000030	0.3768399949999974	0.1664600069999977
0.8264499900000004	0.4421499970000013	0.2775900070000006
0.9345800279999992	0.6448600290000002	0.0553399989999974
0.5299485781133644	0.5101352356545868	0.3954043835279056
0.7340910557815054	0.5790674527917244	0.5254232793577310
0.5274699929999969	0.5091599819999999	0.2498099949999997
0.7284901474840212	0.5779040196344239	0.3631716082858840
0.9227887171265096	0.6415076948746561	0.4739438613652191
0.6355999709999978	0.3785299959999975	0.0275599989999975
0.8315299750000023	0.4438399969999978	0.1386799960000005

0.5944756168433709	0.3650201381228314	0.3359923751749025
0.7870454269788694	0.4303212834989356	0.4451308975368611
0.5030000209999983	0.5009999870000001	0.0000000000000000
0.6989399790000022	0.5663099880000004	0.1111199999999997
0.8948799969999968	0.6316300029999979	0.2222500000000025
0.6938599940000003	0.5646200179999994	0.2500300109999998
0.8977147557781039	0.6327388539545634	0.3618176264573359
0.8019899729999977	0.4339999849999998	0.0277799999999999
0.9979299900000029	0.4993099869999966	0.1388999970000029
0.6034223421949008	0.3684568402063203	0.4788038810368187
0.1305200010000007	0.7101699709999991	0.1664600069999977
0.3264499900000004	0.7754799719999994	0.2775900070000006
0.4345799979999967	0.9781900050000019	0.0553399989999974
0.0337902414206566	0.8433835008815453	0.3992351364708572
0.2263629620850729	0.9154612107780012	0.5143314175444663
0.0274700000000010	0.8424900170000029	0.2498099949999997
0.2271311284831036	0.9033087452971560	0.3637157162158914
0.4231911504229095	0.9798712924377769	0.4740831568298576
0.1356000010000002	0.7118700150000024	0.0275599989999975
0.3315300049999976	0.7771800160000026	0.1386799960000005
0.0904546422054114	0.6962577172038587	0.3359320642312484
0.2964222982812577	0.7638687671702153	0.4559387141027387
0.0030000000000001	0.8343300220000032	0.0000000000000000
0.1989399939999998	0.8996499779999994	0.1111199999999997
0.3948799969999968	0.9649599789999996	0.2222500000000025
0.1938599940000003	0.8979499940000011	0.2500300109999998
0.3965816338638443	0.9655955527651687	0.3613163291385400
0.3019900019999966	0.7673299909999969	0.0277799999999999
0.4979299900000029	0.8326399919999972	0.1388999970000029
0.0920276505816550	0.6933180968521078	0.4930889650070991
0.6305199860000030	0.7101699709999991	0.1664600069999977
0.8264499900000004	0.7754799719999994	0.2775900070000006
0.9345800279999992	0.9781900050000019	0.0553399989999974
0.5277637956873035	0.8424640493043761	0.3960563234612832
0.7307605165069383	0.9103053303509322	0.5294070172625480
0.5274699929999969	0.8424900170000029	0.2498099949999997
0.7273722308908283	0.9094538574016944	0.3628436867126349
0.9237863147850105	0.9734121284567471	0.4732895549707358
0.6355999709999978	0.7118700150000024	0.0275599989999975
0.8315299750000023	0.7771800160000026	0.1386799960000005
0.5921031451611476	0.6976417296392194	0.3360471254597174
0.7872680838770468	0.7640184988250716	0.4461777312113898
0.5030000209999983	0.8343300220000032	0.0000000000000000
0.6989399790000022	0.8996499779999994	0.1111199999999997

0.8948799969999968	0.9649599789999996	0.2222500000000025
0.6938599940000003	0.8979499940000011	0.2500300109999998
0.8982455504186762	0.9633997400951833	0.3603551101935425
0.8019899729999977	0.7673299909999969	0.0277799999999999
0.9979299900000029	0.8326399919999972	0.1388999970000029
0.6028114396817877	0.7026327069499232	0.4777791271367226
0.1285217420639043	0.2765161576508128	0.7606419663348408
0.1821164478352317	0.4131278271627017	0.6111122280305981
0.2033241021693496	0.3957415911474277	0.5138783093515954
0.2032617139497404	0.7368304766680970	0.5133427272209107
0.2748253651754614	0.2909533470815209	0.7117608205243611
0.1919127069267343	0.3409429839131630	0.7173810447701716
0.1369009204728466	0.3269836511983148	0.6583315570707928
0.3141064492265639	0.3060822035862359	0.7543104758518760
0.3184044201191928	0.3355147303791503	0.6741847145058277
0.2545786865616834	0.1923409277299437	0.7030083682488798
0.2122467455670097	0.4394748267320739	0.7289719393914864
0.1545151477880376	0.2851175428777716	0.8016384350544422
0.0709227729814370	0.3444936645989282	0.6669757295589893
0.1285799261426527	0.2363958605196450	0.6380250899515728
0.1831091905755884	0.4968031264279100	0.6226325539014066
0.2181751886005722	0.7461196530073916	0.5859718399955320

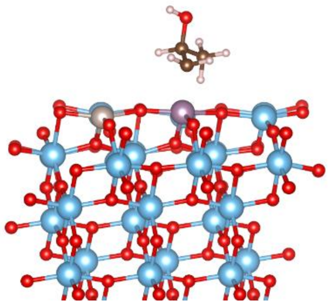

Supplementary Fig. 40c

Ti	O	Mo	Ru	C	H
52	121	1	1	3	7

0.2162500020000024	0.0720800010000033	0.0971200019999969
0.4121899900000017	0.1374000010000032	0.2082500010000032
0.1095586300222826	0.2032697742326720	0.3198519766893657
0.3192685891392509	0.2741482182351587	0.4286642502013557
0.1132100000000023	0.2044000030000035	0.1804700049999965
0.3091399970000026	0.2697100039999967	0.2915900050000033
0.4172700049999989	0.1390900020000032	0.0693399979999967
0.0109536506673976	0.0037839791868302	0.4124419840028337
0.2092025849516084	0.0669040397601058	0.5057394438862185
0.7162500020000024	0.0720800010000033	0.0971200019999969
0.9121900199999970	0.1374000010000032	0.2082500010000032

0.6086523661486906	0.2026526098933866	0.3195670340140340
0.8140477248478316	0.2703555888512227	0.4310740872010607
0.6132100219999970	0.2044000030000035	0.1804700049999965
0.8091400269999980	0.2697100039999967	0.2915900050000033
0.9172700049999989	0.1390900020000032	0.0693399979999967
0.5118117647759322	0.0038870086927716	0.4118909164698698
0.7069807104866866	0.0664490244760194	0.5111462456402294
0.2162500020000024	0.4054200050000034	0.0971200019999969
0.4121899900000017	0.4707300070000002	0.2082500010000032
0.1099559531502185	0.5358668541998540	0.3196142591283391
0.3236606057998960	0.6066131183866705	0.4262683343737939
0.1132100000000023	0.5377399919999988	0.1804700049999965
0.3091399970000026	0.6030499940000027	0.2915900050000033
0.4172700049999989	0.4724200069999966	0.0693399979999967
0.0032098695626487	0.3345109283814626	0.4086902105030636
0.7162500020000024	0.4054200050000034	0.0971200019999969
0.9121900199999970	0.4707300070000002	0.2082500010000032
0.6087174827464361	0.5359595667074285	0.3197440785472150
0.8139359496967804	0.6049127991809832	0.4314707835759236
0.6132100219999970	0.5377399919999988	0.1804700049999965
0.8091400269999980	0.6030499940000027	0.2915900050000033
0.9172700049999989	0.4724200069999966	0.0693399979999967
0.5107460655314620	0.3363971173979400	0.4117868217968602
0.7058919973082510	0.4015931410496662	0.5102942451094443
0.2162500020000024	0.7387499809999980	0.0971200019999969
0.4121899900000017	0.8040599819999983	0.2082500010000032
0.1095662117319547	0.8698449146431685	0.3197768015452807
0.3204493037839372	0.9375315919960892	0.4281764875072379
0.1132100000000023	0.8710700270000018	0.1804700049999965
0.3091399970000026	0.9363800289999986	0.2915900050000033
0.4172700049999989	0.8057600260000015	0.0693399979999967
0.0027741417710497	0.6669320947416421	0.4067887979795481
0.7162500020000024	0.7387499809999980	0.0971200019999969
0.9121900199999970	0.8040599819999983	0.2082500010000032
0.6085751327920930	0.8695805767327854	0.3195680150180178
0.8199579760585807	0.9403212711641153	0.4270460002549232
0.6132100219999970	0.8710700270000018	0.1804700049999965
0.8091400269999980	0.9363800289999986	0.2915900050000033
0.9172700049999989	0.8057600260000015	0.0693399979999967
0.5146159622513128	0.6714508926144790	0.4127043618512666
0.7089121723589540	0.7389412643137887	0.5115859469437043
0.1305200010000007	0.0435100010000014	0.1664600069999977
0.3264499900000004	0.1088199989999978	0.2775900070000006
0.4345799979999967	0.3115299939999971	0.0553399989999974

0.0309649035281972	0.1785404820927151	0.3979305527130388
0.2294463481451286	0.2417455589764757	0.5146608754048511
0.0274700000000010	0.1758199929999975	0.2498099949999997
0.2261720420966917	0.2483884225919223	0.3625409519070503
0.4227353258169989	0.3028022910672509	0.4736826716472208
0.1356000010000002	0.0452000009999978	0.0275599989999975
0.3315300049999976	0.1105099990000014	0.1386799960000005
0.0922627063333698	0.0307703107197299	0.3358484449264287
0.2878633714145680	0.0949363997527311	0.4400132687929855
0.0030000000000001	0.1676699969999973	0.0000000000000000
0.1989399939999998	0.2329799979999976	0.1111199999999997
0.3948799969999968	0.2982900140000027	0.2222500000000025
0.1938599940000003	0.2312899980000012	0.2500300109999998
0.3964625496567570	0.3015148114766736	0.3607638626858795
0.3019900019999966	0.1006600039999981	0.0277799999999999
0.4979299900000029	0.1659799959999972	0.1388999970000029
0.1012532232007603	0.0282776739543920	0.4767310773950728
0.6305199860000030	0.0435100010000014	0.1664600069999977
0.8264499900000004	0.1088199989999978	0.2775900070000006
0.9345800279999992	0.3115299939999971	0.0553399989999974
0.5282828722960776	0.1759657908751417	0.3947538901480508
0.7328875305230050	0.2436891655252102	0.5225088336187000
0.5274699929999969	0.1758199929999975	0.2498099949999997
0.7273110022754516	0.2406840691901069	0.3625407125903028
0.9202450322747772	0.3078887432688607	0.4733973190742743
0.6355999709999978	0.0452000009999978	0.0275599989999975
0.8315299750000023	0.1105099990000014	0.1386799960000005
0.5924123976149263	0.0307537384306130	0.3351018129810974
0.7865283277686018	0.0934265915036712	0.4456221226226050
0.5030000209999983	0.1676699969999973	0.0000000000000000
0.6989399790000022	0.2329799979999976	0.1111199999999997
0.8948799969999968	0.2982900140000027	0.2222500000000025
0.6938599940000003	0.2312899980000012	0.2500300109999998
0.8954229830130312	0.2997413537609063	0.3617362006652968
0.8019899729999977	0.1006600039999981	0.0277799999999999
0.9979299900000029	0.1659799959999972	0.1388999970000029
0.6022549044819427	0.0316365966310839	0.4763481680337553
0.1305200010000007	0.3768399949999974	0.1664600069999977
0.3264499900000004	0.4421499970000013	0.2775900070000006
0.4345799979999967	0.6448600290000002	0.0553399989999974
0.0350031019518335	0.5113664718743584	0.4007023326611631
0.2166347975951547	0.5749682309296068	0.5204018301029476
0.0274700000000010	0.5091599819999999	0.2498099949999997
0.2273896122197114	0.5749758984620424	0.3632399427397643

0.4256354859606112	0.6408897445418202	0.4733620346539837
0.1356000010000002	0.3785299959999975	0.0275599989999975
0.3315300049999976	0.4438399969999978	0.1386799960000005
0.0889053986203991	0.3637498031837541	0.3354606486883551
0.2920468124264090	0.4331418249034870	0.4529913065183435
0.0030000000000001	0.5009999870000001	0.0000000000000000
0.1989399939999998	0.5663099880000004	0.1111199999999997
0.3948799969999968	0.6316300029999979	0.2222500000000025
0.1938599940000003	0.5646200179999994	0.2500300109999998
0.3990137246790443	0.6326226061413270	0.3594741823102882
0.3019900019999966	0.4339999849999998	0.0277799999999999
0.4979299900000029	0.4993099869999966	0.1388999970000029
0.0896872096161173	0.3580668481286225	0.4909756746535131
0.6305199860000030	0.3768399949999974	0.1664600069999977
0.8264499900000004	0.4421499970000013	0.2775900070000006
0.9345800279999992	0.6448600290000002	0.0553399989999974
0.5292218621400749	0.5093171958169685	0.3955191463361277
0.7342572602834658	0.5782726611921170	0.5222353906271205
0.5274699929999969	0.5091599819999999	0.2498099949999997
0.7273094758788337	0.5772952392144800	0.3627367664894025
0.9206502137431511	0.6363543398167322	0.4728422494536230
0.6355999709999978	0.3785299959999975	0.0275599989999975
0.8315299750000023	0.4438399969999978	0.1386799960000005
0.5927815672050815	0.3640349627863880	0.3355463297742085
0.7833555749948438	0.4272869768298385	0.4439777816275063
0.5030000209999983	0.5009999870000001	0.0000000000000000
0.6989399790000022	0.5663099880000004	0.1111199999999997
0.8948799969999968	0.6316300029999979	0.2222500000000025
0.6938599940000003	0.5646200179999994	0.2500300109999998
0.8946688965002909	0.6299296452741714	0.3612934660606763
0.8019899729999977	0.4339999849999998	0.0277799999999999
0.9979299900000029	0.4993099869999966	0.1388999970000029
0.5998272943057278	0.3668517021978344	0.4777388467660601
0.1305200010000007	0.7101699709999991	0.1664600069999977
0.3264499900000004	0.7754799719999994	0.2775900070000006
0.4345799979999967	0.9781900050000019	0.0553399989999974
0.0294626674016411	0.8411372685375420	0.3966127639997170
0.2332547182563169	0.9078382483186710	0.5165566647066167
0.0274700000000010	0.8424900170000029	0.2498099949999997
0.2260730879741380	0.9016408704045965	0.3626766146015725
0.4237108994680962	0.9781994922349407	0.4734307968785074
0.1356000010000002	0.7118700150000024	0.0275599989999975
0.3315300049999976	0.7771800160000026	0.1386799960000005
0.0896508294640598	0.6949891634870088	0.3353016591316970

0.2948235317772240	0.7608657142454786	0.4515746028653327
0.0030000000000001	0.8343300220000032	0.0000000000000000
0.1989399939999998	0.8996499779999994	0.1111199999999997
0.3948799969999968	0.9649599789999996	0.2222500000000025
0.1938599940000003	0.8979499940000011	0.2500300109999998
0.3969466448192691	0.9627275273243039	0.3603250404560622
0.3019900019999966	0.7673299909999969	0.0277799999999999
0.4979299900000029	0.8326399919999972	0.1388999970000029
0.0847009303688089	0.7049311170406469	0.4904619418174508
0.6305199860000030	0.7101699709999991	0.1664600069999977
0.8264499900000004	0.7754799719999994	0.2775900070000006
0.9345800279999992	0.9781900050000019	0.0553399989999974
0.5286212103993095	0.8428344512955364	0.3949644517056037
0.7308050727992402	0.9104505831885009	0.5294204733869520
0.5274699929999969	0.8424900170000029	0.2498099949999997
0.7273156922319608	0.9089676801230759	0.3625520321041151
0.9232626606475611	0.9755560778245231	0.4734562149417215
0.6355999709999978	0.7118700150000024	0.0275599989999975
0.8315299750000023	0.7771800160000026	0.1386799960000005
0.5928781611159296	0.6975015995506335	0.3352573979421639
0.7879855507971734	0.7648466923146719	0.4459347050843174
0.5030000209999983	0.8343300220000032	0.0000000000000000
0.6989399790000022	0.8996499779999994	0.1111199999999997
0.8948799969999968	0.9649599789999996	0.2222500000000025
0.6938599940000003	0.8979499940000011	0.2500300109999998
0.8980513331852639	0.9661953621223198	0.3603875593505119
0.8019899729999977	0.7673299909999969	0.0277799999999999
0.9979299900000029	0.8326399919999972	0.1388999970000029
0.6039720989953196	0.7032526301255614	0.4769392465682477
0.2733871680972461	0.3882610901902230	0.7133281951823509
0.1991701088850474	0.4029641613925030	0.5168381807741874
0.1952519739660926	0.7339842837491551	0.5066147181037980
0.3486594474485165	0.3183742847919244	0.6322573182441334
0.2922668458336335	0.4066160561793590	0.6491285570331644
0.2026114525544586	0.3845584526460187	0.6145523115534305
0.4073177725366176	0.3341282651209589	0.6625216640158245
0.3716048638251013	0.3286787169740558	0.5844780919634641
0.3097235415491811	0.2240213521579628	0.6396719837788276
0.3314334864557840	0.5010848315448373	0.6408272461072406
0.2496028092868876	0.4541114929127980	0.7295229672239159
0.1641933631362160	0.4496629766420365	0.6302226875592080
0.1625278680880525	0.2914787440548878	0.6241680108416544

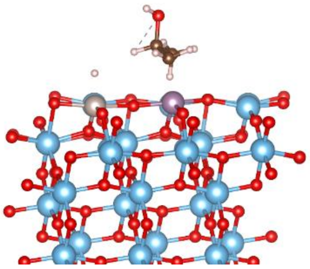

Supplementary Fig. 40d

Ti O Mo Ru C H

52 121 1 1 3 8

0.2162500020000024	0.0720800010000033	0.0971200019999969
0.4121899900000017	0.1374000010000032	0.2082500010000032
0.1092209414220425	0.2031921833508657	0.3192703498221265
0.3239104969887923	0.2751619422033015	0.4254189991897166
0.1132100000000023	0.2044000030000035	0.1804700049999965
0.3091399970000026	0.2697100039999967	0.2915900050000033
0.4172700049999989	0.1390900020000032	0.0693399979999967
0.0101880946042950	0.0033546806910525	0.4116302078264079
0.2077469911178515	0.0674100153705992	0.5058811488405356
0.7162500020000024	0.0720800010000033	0.0971200019999969
0.9121900199999970	0.1374000010000032	0.2082500010000032
0.6086027598724353	0.2026174409739925	0.3191416881741904
0.8140490581895469	0.2704443166792501	0.4307404063073246
0.6132100219999970	0.2044000030000035	0.1804700049999965
0.8091400269999980	0.2697100039999967	0.2915900050000033
0.9172700049999989	0.1390900020000032	0.0693399979999967
0.5125485016860384	0.0041827841152057	0.4120320126563838
0.7073301154402855	0.0668577729590214	0.5113915442649087
0.2162500020000024	0.4054200050000034	0.0971200019999969
0.4121899900000017	0.4707300070000002	0.2082500010000032
0.1099530153692477	0.5361601306064271	0.3193338787106751
0.3218745976969232	0.6012986228247902	0.4276412047630339
0.1132100000000023	0.5377399919999988	0.1804700049999965
0.3091399970000026	0.6030499940000027	0.2915900050000033
0.4172700049999989	0.4724200069999966	0.0693399979999967
0.0030610913132776	0.3343697515694642	0.4086105379964296
0.7162500020000024	0.4054200050000034	0.0971200019999969
0.9121900199999970	0.4707300070000002	0.2082500010000032
0.6085751638091736	0.5363420024998248	0.3193150975306606
0.8143135908280990	0.6057980825712578	0.4315354017323550
0.6132100219999970	0.5377399919999988	0.1804700049999965
0.8091400269999980	0.6030499940000027	0.2915900050000033
0.9172700049999989	0.4724200069999966	0.0693399979999967
0.5146928710637221	0.3378445665916217	0.4127485306796776
0.7081653860641871	0.4026686182598386	0.5112182202232428

0.2162500020000024	0.7387499809999980	0.0971200019999969
0.4121899900000017	0.8040599819999983	0.2082500010000032
0.1093957562195863	0.8695930362840274	0.3194117565042415
0.3209146916875863	0.9428485029753714	0.4279154201859345
0.1132100000000023	0.8710700270000018	0.1804700049999965
0.3091399970000026	0.9363800289999986	0.2915900050000033
0.4172700049999989	0.8057600260000015	0.0693399979999967
0.0025230377373363	0.6666744317111639	0.4068083101600637
0.7162500020000024	0.7387499809999980	0.0971200019999969
0.9121900199999970	0.8040599819999983	0.2082500010000032
0.6085231281877546	0.8695457557436453	0.3191576970825998
0.8194721993967540	0.9401424785984215	0.4271559441537446
0.6132100219999970	0.8710700270000018	0.1804700049999965
0.8091400269999980	0.9363800289999986	0.2915900050000033
0.9172700049999989	0.8057600260000015	0.0693399979999967
0.5134101723057610	0.6713394191160268	0.4125159328537839
0.7080618107258676	0.7388989949689944	0.5115338246480580
0.1305200010000007	0.0435100010000014	0.1664600069999977
0.3264499900000004	0.1088199989999978	0.2775900070000006
0.4345799979999967	0.3115299939999971	0.0553399989999974
0.0297533573612846	0.1781464467327834	0.3967346763470102
0.2284029669328786	0.2416254795731960	0.5230239373457998
0.0274700000000010	0.1758199929999975	0.2498099949999997
0.2272048917638469	0.2471868328259747	0.3625367877351046
0.4249429335152859	0.3030194021744648	0.4731269696162190
0.1356000010000002	0.0452000009999978	0.0275599989999975
0.3315300049999976	0.1105099990000014	0.1386799960000005
0.0925398321679995	0.0306109636472697	0.3357116755869952
0.2882359786475757	0.0985337672765340	0.4407851313697149
0.0030000000000001	0.1676699969999973	0.0000000000000000
0.1989399939999998	0.2329799979999976	0.1111199999999997
0.3948799969999968	0.2982900140000027	0.2222500000000025
0.1938599940000003	0.2312899980000012	0.2500300109999998
0.4000207436630375	0.3024307217640452	0.3591107077257137
0.3019900019999966	0.1006600039999981	0.0277799999999999
0.4979299900000029	0.1659799959999972	0.1388999970000029
0.1006221449245901	0.0335871087003515	0.4759424568548292
0.6305199860000030	0.0435100010000014	0.1664600069999977
0.8264499900000004	0.1088199989999978	0.2775900070000006
0.9345800279999992	0.3115299939999971	0.0553399989999974
0.5298551639297681	0.1765624067907783	0.3952751584955572
0.7337169502458470	0.2440990849807417	0.5231244989764241
0.5274699929999969	0.1758199929999975	0.2498099949999997
0.7274559967784560	0.2412374415165481	0.3627171984531272

0.9201044228703524	0.3082798088245955	0.4732692067882061
0.6355999709999978	0.0452000009999978	0.0275599989999975
0.8315299750000023	0.1105099990000014	0.1386799960000005
0.5924478728538816	0.0306254681443748	0.3352936401619447
0.7865625185109916	0.0936576357963267	0.4457432682501921
0.5030000209999983	0.1676699969999973	0.0000000000000000
0.6989399790000022	0.2329799979999976	0.1111199999999997
0.8948799969999968	0.2982900140000027	0.2222500000000025
0.6938599940000003	0.2312899980000012	0.2500300109999998
0.8955863489300173	0.3002268857789906	0.3615873411707155
0.8019899729999977	0.1006600039999981	0.0277799999999999
0.9979299900000029	0.1659799959999972	0.1388999970000029
0.6024465029308053	0.0318903709900375	0.4767228293093266
0.1305200010000007	0.3768399949999974	0.1664600069999977
0.3264499900000004	0.4421499970000013	0.2775900070000006
0.4345799979999967	0.6448600290000002	0.0553399989999974
0.0352416120977998	0.5114586153037357	0.4010777396132553
0.2168766728402758	0.5675509368957775	0.5095980883457505
0.0274700000000010	0.5091599819999999	0.2498099949999997
0.2274879210743527	0.5780826091488360	0.3635186153384038
0.4241892889900796	0.6414826440752922	0.4738926310494797
0.1356000010000002	0.3785299959999975	0.0275599989999975
0.3315300049999976	0.4438399969999978	0.1386799960000005
0.0903369199283422	0.3643867384996326	0.3358829730155093
0.2922338849371192	0.4272067716177281	0.4520485765194391
0.0030000000000001	0.5009999870000001	0.0000000000000000
0.1989399939999998	0.5663099880000004	0.1111199999999997
0.3948799969999968	0.6316300029999979	0.2222500000000025
0.1938599940000003	0.5646200179999994	0.2500300109999998
0.3978683628873433	0.6291648919972213	0.3604925691846168
0.3019900019999966	0.4339999849999998	0.0277799999999999
0.4979299900000029	0.4993099869999966	0.1388999970000029
0.0873772410273413	0.3515342063293191	0.4924899633149644
0.6305199860000030	0.3768399949999974	0.1664600069999977
0.8264499900000004	0.4421499970000013	0.2775900070000006
0.9345800279999992	0.6448600290000002	0.0553399989999974
0.5304179315833648	0.5100125741915452	0.3961658612477443
0.7350733698076674	0.5792262614891216	0.5226227260945631
0.5274699929999969	0.5091599819999999	0.2498099949999997
0.7280611423175828	0.5780409247659455	0.3630963075962635
0.9219605297555448	0.6398636175014495	0.4730746503067417
0.6355999709999978	0.3785299959999975	0.0275599989999975
0.8315299750000023	0.4438399969999978	0.1386799960000005
0.5938969251013980	0.3647370156144393	0.3356357027758142

0.7846703806346320	0.4283929044859553	0.4444950457831264
0.5030000209999983	0.5009999870000001	0.0000000000000000
0.6989399790000022	0.5663099880000004	0.1111199999999997
0.8948799969999968	0.6316300029999979	0.2222500000000025
0.6938599940000003	0.5646200179999994	0.2500300109999998
0.8953271242498478	0.6295327663163309	0.3614252048213951
0.8019899729999977	0.4339999849999998	0.0277799999999999
0.9979299900000029	0.4993099869999966	0.1388999970000029
0.6018753651847080	0.3677726044599605	0.4788336740969603
0.1305200010000007	0.7101699709999991	0.1664600069999977
0.3264499900000004	0.7754799719999994	0.2775900070000006
0.4345799979999967	0.9781900050000019	0.0553399989999974
0.0306494560916594	0.8413901079984184	0.3973340404821308
0.2315230220686993	0.9129751983368696	0.5153666820648557
0.0274700000000010	0.8424900170000029	0.2498099949999997
0.2265990895015375	0.9011395527537518	0.3632442455515330
0.4237319019138420	0.9783749861062028	0.4735366129971528
0.1356000010000002	0.7118700150000024	0.0275599989999975
0.3315300049999976	0.7771800160000026	0.1386799960000005
0.0895185243163803	0.6951627538814223	0.3353218795140707
0.2957835404932027	0.7632332640082600	0.4539295298649959
0.0030000000000001	0.8343300220000032	0.0000000000000000
0.1989399939999998	0.8996499779999994	0.1111199999999997
0.3948799969999968	0.9649599789999996	0.2222500000000025
0.1938599940000003	0.8979499940000011	0.2500300109999998
0.3973565914880674	0.9659134557019662	0.3603759113045611
0.3019900019999966	0.7673299909999969	0.0277799999999999
0.4979299900000029	0.8326399919999972	0.1388999970000029
0.0907458802484263	0.7039765096179192	0.4925247277017104
0.6305199860000030	0.7101699709999991	0.1664600069999977
0.8264499900000004	0.7754799719999994	0.2775900070000006
0.9345800279999992	0.9781900050000019	0.0553399989999974
0.5276353435266088	0.8425560078234302	0.3949714228561108
0.7308200308127215	0.9105530314295599	0.5292813045097713
0.5274699929999969	0.8424900170000029	0.2498099949999997
0.7272036732754024	0.9090445455048360	0.3625679953165127
0.9229950104492760	0.9747281145399538	0.4732035552529890
0.6355999709999978	0.7118700150000024	0.0275599989999975
0.8315299750000023	0.7771800160000026	0.1386799960000005
0.5922164379309576	0.6974385219536878	0.3355287708311147
0.7871373970043691	0.7646977054635512	0.4458506840541988
0.5030000209999983	0.8343300220000032	0.0000000000000000
0.6989399790000022	0.8996499779999994	0.1111199999999997
0.8948799969999968	0.9649599789999996	0.2222500000000025

0.6938599940000003	0.8979499940000011	0.2500300109999998
0.8977477212751547	0.9654584144587852	0.3603203337579484
0.8019899729999977	0.7673299909999969	0.0277799999999999
0.9979299900000029	0.8326399919999972	0.1388999970000029
0.6029038687070341	0.7031761197971115	0.4772085622039465
0.2927761710537204	0.4583482995220864	0.7074784538033611
0.1978993950122739	0.3954953750695746	0.5165928275079054
0.2013302392677253	0.7356023310857202	0.5091461239731331
0.3687219776034316	0.3999812579208358	0.6232221630868990
0.2988669094528753	0.4675733850221412	0.6420749397955077
0.2055708070842028	0.4136282198661564	0.6143213526959037
0.4308180102155789	0.4369851899276011	0.6487893640841984
0.3840488331764979	0.4087450663130259	0.5739356398770722
0.3453511697169156	0.3026829819638973	0.6343872381351108
0.3217239611281366	0.5641736516473584	0.6280451994410380
0.2558470381524311	0.5101506422447126	0.7233124442101488
0.1587964076072433	0.4704612902767247	0.6256095240283044
0.1781248531453336	0.3213695087726577	0.6328018566921979
0.2195658091864639	0.7367959142963224	0.5811079315904533

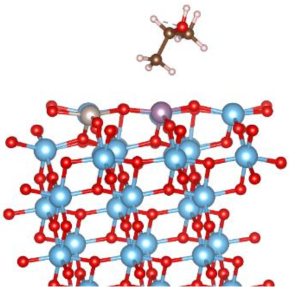

Supplementary Fig. 40e

Ti	O	Mo	Ru	C	H
52	121	1	1	3	8

0.2162500020000024	0.0720800010000033	0.0971200019999969
0.4121899900000017	0.1374000010000032	0.2082500010000032
0.1085931992317214	0.2024156184825995	0.3185862014662020
0.3229417433027227	0.2713666270662597	0.4247553416379158
0.1132100000000023	0.2044000030000035	0.1804700049999965
0.3091399970000026	0.2697100039999967	0.2915900050000033
0.4172700049999989	0.1390900020000032	0.0693399979999967
0.0123492300301127	0.0038850953959322	0.4128151595976717
0.2100275558457382	0.0657911317499839	0.5104309115810467
0.7162500020000024	0.0720800010000033	0.0971200019999969
0.9121900199999970	0.1374000010000032	0.2082500010000032
0.6085913839847052	0.2024794651902127	0.3189825147159567
0.8157012018700890	0.2705838626332598	0.4312328797895050
0.6132100219999970	0.2044000030000035	0.1804700049999965

0.8091400269999980	0.2697100039999967	0.2915900050000033
0.9172700049999989	0.1390900020000032	0.0693399979999967
0.5130960339796202	0.0043469085100468	0.4126313396538193
0.7089795685681595	0.0666082132314030	0.5115358434108501
0.2162500020000024	0.4054200050000034	0.0971200019999969
0.4121899900000017	0.4707300070000002	0.2082500010000032
0.1092316390620896	0.5363897052455902	0.3185371259504473
0.3228985238062790	0.6108047486315080	0.4256343013153815
0.1132100000000023	0.5377399919999988	0.1804700049999965
0.3091399970000026	0.6030499940000027	0.2915900050000033
0.4172700049999989	0.4724200069999966	0.0693399979999967
0.0036623150663268	0.3346696609085157	0.4064695390480372
0.7162500020000024	0.4054200050000034	0.0971200019999969
0.9121900199999970	0.4707300070000002	0.2082500010000032
0.6085906051318738	0.5361626711175401	0.3187975229038693
0.8154503542293416	0.6062884333542015	0.4304987990148733
0.6132100219999970	0.5377399919999988	0.1804700049999965
0.8091400269999980	0.6030499940000027	0.2915900050000033
0.9172700049999989	0.4724200069999966	0.0693399979999967
0.5149835433196104	0.3384100455216234	0.4131974905958894
0.7090389702408173	0.4029371667592518	0.5107640800661796
0.2162500020000024	0.7387499809999980	0.0971200019999969
0.4121899900000017	0.8040599819999983	0.2082500010000032
0.1095875719583394	0.8700983613669092	0.3189667654946212
0.3212915589276897	0.9396488025949254	0.4280946940472809
0.1132100000000023	0.8710700270000018	0.1804700049999965
0.3091399970000026	0.9363800289999986	0.2915900050000033
0.4172700049999989	0.8057600260000015	0.0693399979999967
0.0044017963628823	0.6678416345710697	0.4075992567294128
0.7162500020000024	0.7387499809999980	0.0971200019999969
0.9121900199999970	0.8040599819999983	0.2082500010000032
0.6082502611139262	0.8694848010263984	0.3189967826219572
0.8213706399146811	0.9401854275488052	0.4264471824211450
0.6132100219999970	0.8710700270000018	0.1804700049999965
0.8091400269999980	0.9363800289999986	0.2915900050000033
0.9172700049999989	0.8057600260000015	0.0693399979999967
0.5150491468756248	0.6709024875364358	0.4133100940159010
0.7093092037528974	0.7389777948285849	0.5118474416036742
0.1305200010000007	0.0435100010000014	0.1664600069999977
0.3264499900000004	0.1088199989999978	0.2775900070000006
0.4345799979999967	0.3115299939999971	0.0553399989999974
0.0311175733723382	0.1783987811990287	0.3970957744889021
0.2334509045679136	0.2470439840834684	0.5308298754312882
0.0274700000000010	0.1758199929999975	0.2498099949999997

0.2272479744995319	0.2471614418790780	0.3631870019395091
0.4231965260438961	0.3143553335229927	0.4730390157528981
0.1356000010000002	0.0452000009999978	0.0275599989999975
0.3315300049999976	0.1105099990000014	0.1386799960000005
0.0926117775540131	0.0306958164935108	0.3356746144805476
0.2898676927447349	0.0984200274013433	0.4452468433149231
0.0030000000000001	0.1676699969999973	0.0000000000000000
0.1989399939999998	0.2329799979999976	0.1111199999999997
0.3948799969999968	0.2982900140000027	0.2222500000000025
0.1938599940000003	0.2312899980000012	0.2500300109999998
0.3995891146566727	0.2967063102208602	0.3592325155027687
0.3019900019999966	0.1006600039999981	0.0277799999999999
0.4979299900000029	0.1659799959999972	0.1388999970000029
0.1049455453626712	0.0404677830975438	0.4778794270289750
0.6305199860000030	0.0435100010000014	0.1664600069999977
0.8264499900000004	0.1088199989999978	0.2775900070000006
0.9345800279999992	0.3115299939999971	0.0553399989999974
0.5298817842592055	0.1768960188596874	0.3959172598217796
0.7357647768949758	0.2446863731792539	0.5225387810270155
0.5274699929999969	0.1758199929999975	0.2498099949999997
0.7285346235312080	0.2403186425004186	0.3633945711362539
0.9238500686140987	0.3094209995936972	0.4727683047271617
0.6355999709999978	0.0452000009999978	0.0275599989999975
0.8315299750000023	0.1105099990000014	0.1386799960000005
0.5922229054619179	0.0305355441078614	0.3354730335671969
0.7892960401710166	0.0937845232302638	0.4464032514547350
0.5030000209999983	0.1676699969999973	0.0000000000000000
0.6989399790000022	0.2329799979999976	0.1111199999999997
0.8948799969999968	0.2982900140000027	0.2222500000000025
0.6938599940000003	0.2312899980000012	0.2500300109999998
0.8961483622312104	0.3000706423476774	0.3611249323214719
0.8019899729999977	0.1006600039999981	0.0277799999999999
0.9979299900000029	0.1659799959999972	0.1388999970000029
0.6042091266521470	0.0324669342950743	0.4768117292997564
0.1305200010000007	0.3768399949999974	0.1664600069999977
0.3264499900000004	0.4421499970000013	0.2775900070000006
0.4345799979999967	0.6448600290000002	0.0553399989999974
0.0352912463696147	0.5117433525650988	0.4000121180495517
0.2240250913170029	0.5732074787653049	0.5229595565249313
0.0274700000000010	0.5091599819999999	0.2498099949999997
0.2275856026504347	0.5747310856952269	0.3639628958419805
0.4237635550540945	0.6271737197927779	0.4730738025494978
0.1356000010000002	0.3785299959999975	0.0275599989999975
0.3315300049999976	0.4438399969999978	0.1386799960000005

0.0907444172570580	0.3642897804270088	0.3348440494693276
0.2852740845706275	0.4271100622344854	0.4486620867874587
0.0030000000000001	0.5009999870000001	0.0000000000000000
0.1989399939999998	0.5663099880000004	0.1111199999999997
0.3948799969999968	0.6316300029999979	0.2222500000000025
0.1938599940000003	0.5646200179999994	0.2500300109999998
0.3990501384441695	0.6380210655188450	0.3597538273178110
0.3019900019999966	0.4339999849999998	0.0277799999999999
0.4979299900000029	0.4993099869999966	0.1388999970000029
0.1017611877003885	0.3599913139415238	0.4843908033698465
0.6305199860000030	0.3768399949999974	0.1664600069999977
0.8264499900000004	0.4421499970000013	0.2775900070000006
0.9345800279999992	0.6448600290000002	0.0553399989999974
0.5296633532108261	0.5094164911030353	0.3951684517187515
0.7349136323070090	0.5786388680564234	0.5232610639528551
0.5274699929999969	0.5091599819999999	0.2498099949999997
0.7279915592029516	0.5777482846809190	0.3631080252137525
0.9220035536601160	0.6388288123422913	0.4730060683336348
0.6355999709999978	0.3785299959999975	0.0275599989999975
0.8315299750000023	0.4438399969999978	0.1386799960000005
0.5940015199241218	0.3644686609362376	0.3361193197171444
0.7856787799435621	0.4284730227806285	0.4442213882695300
0.5030000209999983	0.5009999870000001	0.0000000000000000
0.6989399790000022	0.5663099880000004	0.1111199999999997
0.8948799969999968	0.6316300029999979	0.2222500000000025
0.6938599940000003	0.5646200179999994	0.2500300109999998
0.8963419331945066	0.6320092864890879	0.3612778149548275
0.8019899729999977	0.4339999849999998	0.0277799999999999
0.9979299900000029	0.4993099869999966	0.1388999970000029
0.6020569810799209	0.3667279213241388	0.4789199169514597
0.1305200010000007	0.7101699709999991	0.1664600069999977
0.3264499900000004	0.7754799719999994	0.2775900070000006
0.4345799979999967	0.9781900050000019	0.0553399989999974
0.0335877892529933	0.8429602128544329	0.3989477070455658
0.2288872382036705	0.9067104979920606	0.5185274654840998
0.0274700000000010	0.8424900170000029	0.2498099949999997
0.2272212084065079	0.9046984185887894	0.3636363711357840
0.4245884125816367	0.9812174509770819	0.4738373857904090
0.1356000010000002	0.7118700150000024	0.0275599989999975
0.3315300049999976	0.7771800160000026	0.1386799960000005
0.0906488005167203	0.6961840585250925	0.3353610770920671
0.2995241357820248	0.7646270078515898	0.4541661112654834
0.0030000000000001	0.8343300220000032	0.0000000000000000
0.1989399939999998	0.8996499779999994	0.1111199999999997

0.3948799969999968	0.9649599789999996	0.2222500000000025
0.1938599940000003	0.8979499940000011	0.2500300109999998
0.3976069895195204	0.9635538504921070	0.3607980295339860
0.3019900019999966	0.7673299909999969	0.0277799999999999
0.4979299900000029	0.8326399919999972	0.1388999970000029
0.0876635088123600	0.6970607512062263	0.4914193722268072
0.6305199860000030	0.7101699709999991	0.1664600069999977
0.8264499900000004	0.7754799719999994	0.2775900070000006
0.9345800279999992	0.9781900050000019	0.0553399989999974
0.5290039247994283	0.8425956948036816	0.3962692099820109
0.7313534786071627	0.9101252263182652	0.5303980176966504
0.5274699929999969	0.8424900170000029	0.2498099949999997
0.7282210158108933	0.9097606974082806	0.3629972775211138
0.9249868580126032	0.9737485572234369	0.4735452146538297
0.6355999709999978	0.7118700150000024	0.0275599989999975
0.8315299750000023	0.7771800160000026	0.1386799960000005
0.5926297895589392	0.6975432545253019	0.3358951762840151
0.7881686592449237	0.7649889897305437	0.4461229574326400
0.5030000209999983	0.8343300220000032	0.0000000000000000
0.6989399790000022	0.8996499779999994	0.1111199999999997
0.8948799969999968	0.9649599789999996	0.2222500000000025
0.6938599940000003	0.8979499940000011	0.2500300109999998
0.8998434705299742	0.9657865313140528	0.3603780629219596
0.8019899729999977	0.7673299909999969	0.0277799999999999
0.9979299900000029	0.8326399919999972	0.1388999970000029
0.6035944635970915	0.7032161007656763	0.4778434388771029
0.1152643816127475	0.3017082485365412	0.7155526841913205
0.2074441121077147	0.4004941753120829	0.5154764839219376
0.1982786054664423	0.7306817251554589	0.5085410702202783
0.2671518522753860	0.2744090498643654	0.6998194251787641
0.2058603597950996	0.3615364253926446	0.7011065983660556
0.1999269166794936	0.4235101134483849	0.6389796712332689
0.2688413867650237	0.2328495810118689	0.7451471618639860
0.3361772246968496	0.3225140028536309	0.6879634668247999
0.2429631827200058	0.2011667579959936	0.6663242714308968
0.2322095531194843	0.4352862015623603	0.7343873222850633
0.1106869027065035	0.2822996022186511	0.7591548157023541
0.1556000470489069	0.3500298290856610	0.6108521381414277
0.2684267083361990	0.4579859386109654	0.6205190202235424
0.1675946703454216	0.4992529247704980	0.6427309810123911

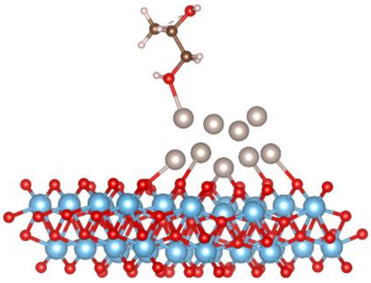

Supplementary Fig. 41a

Ti	O	Ru	C	H
32	66	9	3	8

0.0463498786224766	0.1243923137569312	0.1768173641709963
0.1702752075045776	0.0016876798958911	0.2889042592467466
0.2961085159409480	0.1240173236437326	0.1762005762307477
0.4208677260113971	0.0015286662397483	0.2907065425943183
0.0459940571505533	0.3733235910944137	0.1674079311118626
0.1699905000613932	0.2540773311154575	0.2828977159751644
0.2953927277950317	0.3737886497275423	0.1676978609147962
0.4160500118417559	0.2534705634675964	0.2816514753869746
0.5463083359181304	0.1254266985574531	0.1804436496013695
0.6703779459355513	0.0025835633582283	0.2927819870627188
0.7955484709167302	0.1256361285678719	0.1813640396401882
0.9199454014497687	0.0015986947973821	0.2912383270986912
0.5454671304877861	0.3723641846978772	0.1671897336250078
0.6707244171118240	0.2603726295867378	0.2898899352367743
0.7960676804313553	0.3724948574778080	0.1674041706705648
0.9229918781006534	0.2545376670529980	0.2836362322451183
0.0460334847587134	0.6301796670678143	0.1670853109744503
0.1725346945937763	0.5018022023770565	0.2768379481357495
0.2953627760697333	0.6297006367320056	0.1677686241648744
0.4174955809082631	0.5015201381645916	0.2727889236179740
0.0465808009954831	0.8789083745204898	0.1767200927598730
0.1707270315477650	0.7492268953899694	0.2830529579797721
0.2960147802118918	0.8788921082783469	0.1763754423268797
0.4179903323217311	0.7492495903774676	0.2822076928405426
0.5456641866607329	0.6304642140711840	0.1670814960006796
0.6712799670723767	0.5018627862167034	0.2661350946887093
0.7961213191009686	0.6304092404283053	0.1668422055893496
0.9285613613089774	0.5019432850474114	0.2710987267737412
0.5464356039763658	0.8783787895835425	0.1802962676967173
0.6705742240611798	0.7449602321307427	0.2875225534668197
0.7958734762153639	0.8781908782737085	0.1807637125289429
0.9228802376690221	0.7486858666142477	0.2832200211120291
0.1703773121669730	0.1271869278981799	0.2572934046961879
0.9184565256660706	0.1234907321946569	0.1268970197042021
0.0447608343072514	0.0016205947604327	0.2102739974632360

0.0466495014471310	0.0018976414137718	0.3426228761794888
0.4192179684497838	0.1263842593911135	0.2585592057649423
0.1706189952049934	0.1213509830486026	0.1237712344302495
0.2957904309547599	0.0014557707652457	0.2096947500927402
0.2943660665163023	0.0016676730404745	0.3421990303319058
0.1709888592901565	0.3772200717921502	0.2469960648877021
0.9209046234990621	0.3669919605876478	0.1147960067637001
0.0466070494463224	0.2501284752626757	0.2036568138121859
0.0470615737243814	0.2602966315020995	0.3369600779960940
0.4202692952428523	0.3763058055427298	0.2463864635325258
0.1709341319990077	0.3700830066147625	0.1147601303883681
0.2947435005350427	0.2500435871496648	0.2032686213101206
0.2932839685649290	0.2592560807516218	0.3363253214967187
0.6711728934375595	0.1294251473330403	0.2644991305006174
0.4233533338422790	0.1226590069853779	0.1257839999674674
0.5482824346200526	0.0017643234053225	0.2133040803391106
0.5439563527506130	0.0018585862513600	0.3449262193759157
0.9209159416762215	0.1274803364906637	0.2605723366379867
0.6714009055590557	0.1233310817600431	0.1284263894378420
0.7942462751556861	0.0017841841777574	0.2136357859078884
0.7967846864851916	0.0018500672702034	0.3452474152094803
0.6694029364351077	0.3754887789893850	0.2450693963795824
0.4203209083193786	0.3677136355010562	0.1149275408589544
0.5458015041842879	0.2504572665961504	0.2077921288412918
0.5355613809418207	0.2568064772737186	0.3434371437129104
0.9226272802049038	0.3764520428801620	0.2459701599863442
0.6709264819029122	0.3660149736836491	0.1148899505621275
0.7955412100993382	0.2509912109339358	0.2092579429805789
0.8052081070165218	0.2668027515748798	0.3448162208293997
0.1712138079619115	0.6263284382807524	0.2468633697564130
0.9211278937699959	0.6357964614959165	0.1143550994590666
0.0464811777682998	0.5017944596021418	0.1936195701622615
0.0472716553639190	0.5021420473703113	0.3289852329750815
0.4211495646858296	0.6268962770614622	0.2466726897242871
0.1713174221234160	0.6334225791127098	0.1146286873036805
0.2955658113842715	0.5017674716279116	0.1947742507234128
0.2960922752997899	0.5019231754013741	0.3294317918269358
0.1704975583747691	0.8760559686693355	0.2572568216497840
0.9188593280224830	0.8805105851677868	0.1265734247597613
0.0467654069118942	0.7532949789308848	0.2033812096881213
0.0469672479626025	0.7432292406797896	0.3365435321948432
0.4202293578026768	0.8764334252262852	0.2591069193193585
0.1709460098901557	0.8817558754205098	0.1237132194860924
0.2954906675836500	0.7531584547036533	0.2035074702278352

0.2942298820987042	0.7437313032727537	0.3363477573463560
0.6704648761289518	0.6279948339127602	0.2446780320000835
0.4205182686520131	0.6354859822841503	0.1150685621878987
0.5444063717477374	0.5015656005883194	0.1914753535104745
0.5400842386288678	0.5003414139702191	0.3262923146903696
0.9227384669495506	0.6272080410383183	0.2452488277918089
0.6708003134693906	0.6364784042951339	0.1144773431860183
0.7968293222590804	0.5015047411569373	0.1902705220332390
0.8044182614817270	0.5021488025769434	0.3229318662388815
0.6710785066424801	0.8739919235410023	0.2639930556917508
0.4233444265897984	0.8805897741019909	0.1259863772815706
0.5466511327339343	0.7528833986257337	0.2077032216920833
0.5380142002672145	0.7416329187756016	0.3441056839458231
0.9204758900509193	0.8758485751582912	0.2602663835601807
0.6713053725554944	0.8803670190946377	0.1281274210001453
0.7953718070918228	0.7526232260975367	0.2080434063346857
0.8036764999356302	0.7363042030407337	0.3436805018480958
0.6625659969173102	0.3734212097432778	0.7947221840321640
0.6348283479670916	0.3007111210770165	0.6183562666964226
0.6556463945834137	0.3491837072388269	0.5153744396591489
0.5808036589016036	0.3515429387856130	0.4073800203161551
0.7816103649470513	0.3597722008822244	0.4253430411866340
0.6001384495815207	0.4961694404675172	0.4998992577559110
0.6599208925468562	0.6416116659824055	0.5198706671867371
0.8020390867192253	0.5030074628706898	0.4822532584635433
0.5758451827387137	0.6485336789053119	0.4142866444070504
0.6780612302308320	0.4997691082950647	0.3866292149645241
0.7757704892585386	0.6429506706481474	0.4229160750706950
0.5028479891427897	0.3065153812372415	0.7477322573536737
0.6267592796800473	0.3155722523906221	0.7421148666021047
0.6638715624384057	0.3567235611485818	0.6757643249106970
0.4810002564329464	0.2732606976909551	0.7947351928127042
0.4694217316788384	0.2677456013084407	0.7058166821306487
0.4645860926058138	0.3728542042976859	0.7473818938577085
0.6655569160945931	0.2489813974781209	0.7474679560715282
0.7409931698415951	0.3653387581111729	0.8023023469820608
0.6219204890757519	0.4203219429146080	0.6667593247381648
0.7532986925866099	0.3682219331706422	0.6757187631674124
0.6584577534432573	0.2395659939401800	0.6267196901908940

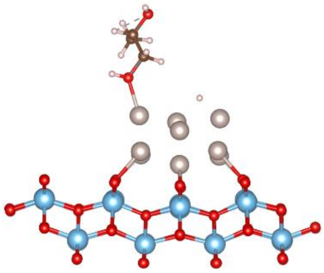

Supplementary Fig. 41b

Ti	O	Ru	C	H
32	66	9	3	9

0.0433905095138585	0.1232684258338156	0.1768226546366942
0.1680115118410606	0.0008603769830012	0.2892686571828069
0.2929196911773139	0.1236334875919200	0.1768579402070150
0.4184583468272591	0.0010214089042886	0.2911396075776015
0.0435102098177148	0.3721715617127273	0.1671643934948550
0.1688721789872068	0.2533545133594107	0.2832520399216414
0.2928733537706061	0.3725858779785998	0.1677271189439260
0.4164870642639667	0.2533066300881076	0.2829403715977335
0.5435129203364928	0.1238853909566819	0.1807418999414367
0.6680939884581302	0.0002305898884840	0.2930744748568377
0.7931184191320754	0.1242097137507467	0.1808223539390655
0.9176741033194388	0.0005869715610512	0.2913746599681086
0.5428624640336781	0.3718231272605531	0.1672550799556133
0.6667166575557056	0.2575958996077168	0.2879555624650730
0.7934429320705401	0.3716166710846758	0.1670739400375687
0.9211869821502937	0.2533449349564913	0.2826481793101586
0.0434349941912603	0.6293390549273150	0.1668341508550446
0.1702146711347927	0.5008727252289904	0.2764739878436170
0.2928092438931935	0.6285731128681783	0.1673356885777517
0.4150174123368990	0.5006603563724300	0.2725442338256384
0.0439690744750105	0.8780953733547430	0.1769513992793640
0.1676500516482894	0.7481632982947786	0.2826793559467392
0.2931083117184379	0.8786580414130345	0.1763597122360457
0.4136931456459195	0.7488104276644372	0.2811836098848537
0.5427766907109511	0.6301700656655215	0.1670060481303311
0.6688231285553515	0.5007596642296452	0.2658538375158266
0.7936834951785174	0.6299018784252429	0.1669535456462766
0.9264199337291616	0.5011224190271790	0.2705691278471995
0.5434924291815243	0.8771454967590657	0.1805548684212977
0.6691681641675008	0.7422467405557519	0.2889887675123379
0.7929939679270478	0.8769684522417531	0.1815423967823453
0.9206443305807379	0.7473961456043352	0.2836989126691239
0.1678899481569300	0.1264035640999364	0.2577079441623654
0.9159923010652997	0.1213859024365805	0.1264398601116877
0.0422477660609153	0.0006189554064887	0.2106395924813705

0.0442088900503572	0.0009492646987870	0.3428864791828558
0.4180507097909645	0.1260873203265235	0.2600309043660648
0.1681958771393221	0.1204695212474633	0.1240777824839351
0.2933413886620349	0.0009651513433226	0.2103038282966827
0.2919956549944441	0.0007905297391717	0.3426612618663332
0.1684580807354249	0.3762514217421553	0.2468964234893286
0.9183976520137609	0.3661448469511098	0.1145765637338752
0.0438222918389030	0.2490480807336657	0.2034454454924073
0.0444227176369499	0.2595373823630547	0.3365025603381965
0.4180220069641480	0.3755601710487040	0.2464500470011067
0.1685299979238402	0.3685538009779954	0.1146893030498327
0.2927781165128063	0.2492505834629084	0.2039576194654792
0.2917606488435400	0.2588307509720557	0.3366875111304886
0.6678705117849294	0.1284887949081746	0.2644317916315223
0.4203870886912663	0.1217802993514372	0.1265505210316268
0.5453118835983641	0.0005413045743564	0.2138220919139578
0.5416428441580861	0.0005108562482420	0.3452682724086623
0.9185735404211397	0.1262477991888106	0.2598689745134382
0.6682772677053888	0.1215568835791481	0.1284619101147021
0.7913674602131607	0.0006113930111822	0.2141068305683230
0.7944549927877244	0.0011559447894173	0.3454426539567252
0.6677771025032951	0.3744020697579349	0.2451867735629365
0.4179158774079769	0.3665249091478124	0.1149918271772394
0.5437404053003877	0.2495466091251624	0.2082501137308036
0.5351549963155436	0.2648401428066998	0.3449064267199948
0.9193428155382695	0.3755512975727793	0.2458223013865403
0.6681092919442251	0.3652984408447013	0.1147869659633115
0.7928193624593076	0.2495648416179145	0.2078678866980210
0.8015066628186962	0.2627323309903926	0.3429718354506233
0.1686792371354027	0.6252934593293366	0.2465490182004164
0.9183197007840614	0.6356274700791272	0.1142504297219997
0.0440255455457669	0.5007434740150221	0.1933515483236002
0.0448365975699731	0.5011696721524698	0.3285847286306822
0.4178552120166885	0.6259871797981468	0.2461196607003782
0.1686084558575366	0.6324825503136757	0.1143375887352454
0.2929419514066673	0.5006402998972078	0.1944899389482541
0.2936455300293557	0.5009898218486933	0.3291721879554140
0.1679034347037849	0.8752373082285078	0.2575068763253471
0.9159271013541839	0.8793917657698894	0.1272253497442208
0.0444528656098856	0.7524973680148315	0.2035692719625181
0.0447939844874884	0.7413845569706214	0.3367743500949986
0.4173755196529957	0.8756195769309396	0.2593463150618348
0.1681104260309419	0.8814807834414914	0.1237517268218407
0.2925002591796827	0.7525152964886265	0.2031112513601442

0.2912143424578291	0.7423226123273680	0.3359583447357555
0.6671985913748643	0.6270824163896658	0.2450740815407156
0.4178914354710865	0.6347281689863936	0.1146867378932547
0.5420056281671103	0.5008077384608842	0.1913756897576530
0.5375214437684163	0.5010099106359360	0.3260939176069552
0.9210715603436647	0.6264007966578428	0.2449720250906781
0.6683148588382545	0.6365927122484359	0.1147710712362815
0.7939793888284998	0.5010619025395633	0.1898768797299575
0.8020954932425483	0.5037879850419146	0.3223310514150343
0.6688297350948129	0.8724667129396411	0.2651614781529886
0.4206162257696339	0.8802230772690083	0.1261492191503429
0.5432581312969399	0.7520470952656509	0.2075357924744227
0.5330910843325991	0.7390912965565176	0.3425966621443531
0.9179904058685388	0.8745236922031454	0.2611536575651527
0.6687629202374238	0.8793078436042553	0.1287216801554492
0.7928064012927586	0.7515248384572938	0.2093277476334930
0.8031314809965752	0.7325720053807664	0.3451202460490220
0.6582738431978311	0.3832632486199727	0.8019279850016873
0.6915982219766871	0.3101168394761837	0.6262393037860999
0.6820386529004685	0.3508455899391450	0.5216734356434307
0.5829686017235962	0.3567926856101967	0.4139737123977014
0.7829217096562999	0.3554651570398401	0.4229041377229699
0.6056284895949877	0.4896842890432077	0.5033887244576103
0.6492020774753505	0.6487562498001481	0.5133557642560964
0.8046123208919579	0.4997929062506932	0.4802940642513698
0.5745689723013663	0.6461299948229791	0.4101739629741240
0.6768100424604092	0.4995978839041736	0.3862973890521366
0.7783661306614097	0.6411132177788403	0.4248038797427256
0.5167226006884131	0.3214989740637821	0.7341017999426870
0.6399456317691956	0.3269757502112435	0.7452302580531979
0.7011511984654956	0.3670912022772415	0.6854270654293126
0.4770321981576520	0.2887478199050788	0.7767789616355025
0.4977734284828944	0.2837918350988407	0.6884494542549395
0.4824491612418823	0.3889799214162161	0.7291058490893355
0.6729436338453869	0.2591377384468005	0.7547623293643622
0.7320670336370777	0.3734690727635334	0.8193215326663059
0.6648878287436105	0.4310588217108131	0.6706117971665958
0.7888765967891829	0.3769372729942912	0.6969485357232689
0.7168209199296771	0.2500378915251752	0.6367040409267352
0.5822792762958686	0.5736643168440004	0.5701006290530197

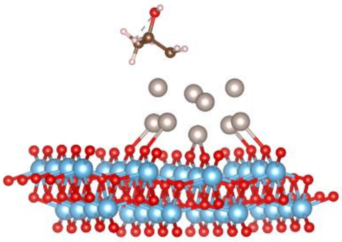

Supplementary Fig. 41c

Ti	O	Ru	C	H
32	65	9	3	7

0.0460071151400567	0.1234319293059808	0.1783301975291050
0.1703102245522704	0.0010810654778388	0.2911402491619955
0.2950798650934665	0.1237584335226891	0.1785485890013586
0.4208321805445528	0.0010379177335290	0.2929185099209530
0.0457271476334175	0.3724845419367306	0.1684733717415828
0.1716681029172797	0.2535723390775838	0.2848005497396526
0.2951674770151299	0.3730822269145935	0.1697075010744558
0.4194267082671391	0.2534083578100600	0.2845847112572439
0.5456355288538256	0.1241995869630498	0.1820454404193479
0.6705148822930802	0.0008613264511105	0.2945252657021258
0.7956920099385063	0.1243942380302678	0.1820474378968401
0.9201145794443176	0.0009019809322819	0.2928573240731200
0.5451402055237079	0.3721151377382351	0.1690273537954957
0.6698648726739399	0.2578162568742465	0.2887515532598277
0.7958905303192425	0.3720415221931715	0.1683248513281421
0.9238113247033545	0.2537361162710425	0.2838665809496903
0.0457635422611459	0.6294392463163161	0.1689736987619717
0.1725852309389767	0.5009034378363775	0.2784986031280782
0.2952708828735995	0.6287177489490906	0.1698710660074246
0.4180097540971716	0.5007437868234952	0.2752483934556448
0.0459417561368887	0.8781818898172506	0.1788639042084393
0.1707762555872667	0.7484383713945851	0.2851252066092231
0.2954111409857619	0.8784448884992060	0.1786375178372402
0.4178637526631178	0.7487268058412788	0.2840657870887301
0.5451362879674914	0.6298441942511362	0.1692331151483189
0.6706209156882003	0.5007362262210693	0.2686903563725892
0.7956254051006381	0.6295615902095352	0.1687427832888896
0.9284845534574371	0.5007668919700583	0.2722120278252706
0.5456341802050643	0.8775970215098212	0.1820996834783356
0.6711614315119989	0.7438720426378433	0.2893835504147163
0.7955056673001709	0.8773518840869667	0.1825596858967375
0.9231852838205333	0.7476911805689817	0.2851177206066824
0.1704147531417603	0.1265881432386071	0.2592985465676281
0.9186497382928499	0.1212487493680404	0.1279325108459105
0.0445923601891425	0.0009140911795687	0.2124120431354962

0.0464015585979140	0.0015189801888690	0.3445879782586270
0.4211340866764801	0.1266956857333274	0.2619006849788302
0.1708006836586129	0.1204244991416251	0.1256553872651640
0.2960520890274559	0.0011576164253240	0.2122345294221746
0.2944764573046063	0.0011321427454840	0.3444554835213307
0.1707689946959978	0.3764087776388134	0.2486465659275667
0.9208423549898324	0.3666005632080586	0.1157818193106653
0.0466891766660270	0.2490855125185304	0.2046616552700126
0.0468596422905091	0.2598949865013643	0.3377553035457606
0.4200626799639557	0.3755714703903942	0.2484992234179052
0.1712964292237030	0.3692596271604681	0.1164597421371939
0.2950523388001627	0.2494083371769491	0.2055896226670389
0.2945447274178604	0.2592312909434545	0.3382681128846116
0.6707011610746729	0.1291626137602993	0.2661292518896034
0.4224854386893235	0.1218876618334895	0.1281511804050156
0.5477306913526954	0.0010365369564321	0.2153155503997492
0.5442640897253128	0.0010325562968161	0.3467898361618233
0.9205738119175690	0.1268320312438427	0.2615627053451645
0.6705601041607826	0.1219372846285282	0.1299766276558418
0.7936084431934352	0.0009621578661235	0.2154419766437449
0.7967218652430068	0.0013479197041199	0.3467682655064260
0.6702516997605416	0.3744973265601630	0.2466753137797763
0.4201644431115963	0.3673989424224211	0.1169707539309161
0.5460502860106617	0.2497615732263997	0.2095139897482379
0.5382823396196614	0.2664913179735472	0.3464908351365688
0.9227738273549442	0.3753445111295362	0.2465286888575140
0.6700974928517522	0.3660298546747854	0.1164151319770715
0.7953628287497156	0.2498219621233272	0.2089576033422071
0.8047909771253898	0.2667003880556481	0.3441483380954286
0.1706921756610521	0.6255800456426106	0.2489955887049974
0.9207217657041095	0.6353260499605192	0.1164010598022065
0.0463979653430186	0.5010327205718520	0.1950566570851037
0.0468885421494785	0.5006135203148245	0.3303200796425471
0.4203471487620046	0.6260561051693372	0.2486471760250996
0.1710991322683446	0.6331193889446662	0.1168331859407382
0.2951335127119739	0.5009473125143152	0.1968389076932358
0.2958660407839480	0.5008760857141547	0.3313388934860038
0.1702709931169969	0.8754828014288405	0.2596948550874074
0.9185535448531383	0.8797939438044826	0.1284860491387831
0.0464364538499742	0.7526236440250467	0.2056380969582409
0.0468398081523630	0.7420035010703359	0.3386428544610364
0.4203890915778781	0.8758190905438398	0.2614550737903070
0.1705762507979429	0.8812579741308678	0.1260810675964672
0.2951762268831030	0.7526018512084686	0.2057273633148484

0.2943455685714003	0.7430128663495346	0.3383651290708904
0.6698836889679642	0.6271221781370915	0.2469733731831473
0.4201092186995671	0.6344054260337789	0.1170941742566617
0.5437644312361116	0.5008357665586494	0.1940990114831887
0.5397963416454596	0.4998971348408007	0.3292751702733148
0.9220772790421202	0.6263484117507193	0.2473369360840158
0.6701429695625677	0.6357632536336362	0.1166286367974469
0.7962654581745996	0.5008932546715757	0.1918221962268407
0.8043654625315039	0.4991752480134213	0.3240633251542884
0.6707964011781343	0.8730892207430327	0.2661925689307691
0.4225308614496180	0.8801346848126386	0.1280478348512704
0.5458049343961413	0.7522191519530267	0.2094689324379469
0.5373444857045657	0.7377121170457610	0.3453796398685359
0.9204250770380511	0.8750829667691276	0.2622331494900608
0.6707428352182403	0.8796391969651324	0.1301891106130345
0.7948499113662909	0.7519179092108591	0.2100722480477144
0.8035694047252598	0.7352602254526389	0.3456286532808520
0.6263968391103816	0.3444145234365746	0.7324942441915723
0.6821347881291649	0.3446338362210025	0.5213490366327241
0.5847295955070211	0.3497365613270997	0.4216366161266310
0.7878066290833015	0.3576940927804458	0.4243772660763328
0.6116547819925392	0.4979158123191457	0.5044996904631391
0.6647353211443898	0.6458822479936184	0.5205853948405729
0.8093437043307569	0.4997362995937589	0.4808946676091804
0.5773000737061318	0.6457355989743887	0.4162499466720763
0.6796306141178773	0.4997548634167238	0.3891087231354135
0.7794320804691834	0.6437185154698400	0.4259736267976192
0.5007194339677375	0.3080580831361852	0.6460461594004776
0.6215942377284714	0.3211793036043640	0.6624719621413503
0.6754514216457822	0.3939272681314614	0.6200166106621113
0.4639112882854571	0.2583018252641968	0.6794536987844781
0.4908106371249849	0.2855570823512597	0.5939614328824023
0.4560505440623348	0.3713077737806846	0.6518860922792920
0.6657545749090876	0.2573006160914147	0.6548907570471625
0.7032702281580583	0.3485910162585470	0.7458039229461024
0.6360834180629024	0.4588276596172662	0.6304047929944044
0.7638804999548479	0.4012887210666873	0.6317304295297119

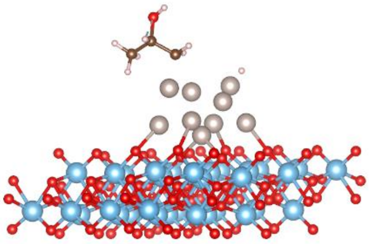

Supplementary Fig. 41d

Ti	O	Ru	C	H
32	65	9	3	8

0.0534134948270272	0.1217977075552435	0.1806366926667007
0.1778636808379487	-0.0011044853021215	0.2929815314954907
0.3023852298074033	0.1212062600250616	0.1802307168723480
0.4281923758565240	-0.0015368453343131	0.2946970642461784
0.0527723345454553	0.3704977840634144	0.1709652805786671
0.1776888545837402	0.2515472325542652	0.2867260785948951
0.3022739083851626	0.3711806513888605	0.1711635679293399
0.4245273279999837	0.2510307736816946	0.2850773530925438
0.5524623922042817	0.1222685790812468	0.1835063408904017
0.6778365931500158	-0.0008657103450971	0.2965264497740586
0.8029421523702670	0.1224378763079241	0.1843757663357548
0.9274486937283525	-0.0006920364433536	0.2947610420125396
0.5522744206245497	0.3698162858008096	0.1706512307581531
0.6790266626044871	0.2554771448088742	0.2906923097904633
0.8027205628660428	0.3702437471189273	0.1706506102667404
0.9298529224958022	0.2525329425274888	0.2874050508334136
0.0528699755400370	0.6272762440319173	0.1710364631393092
0.1783602900696726	0.4988849503527871	0.2805553573630614
0.3024136328574786	0.6271113852813331	0.1717421035928491
0.4230975283672569	0.4988671442916942	0.2764263069607292
0.0530121909430935	0.8764397983367153	0.1804446865649293
0.1788689849208542	0.7463727769147587	0.2872672654234173
0.3023165717717161	0.8757183636294686	0.1808628626173260
0.4272271174879195	0.7454768843734748	0.2878471984799101
0.5526078182418364	0.6275738610206063	0.1711126014149849
0.6770325892676016	0.4989498601408459	0.2702245500549684
0.8027329915772241	0.6277800531638548	0.1706149960822185
0.9342007498004008	0.4988478859032919	0.2747794340893939
0.5527595909119548	0.8755702960000677	0.1843273382477335
0.6771850414333503	0.7429241829638558	0.2907368406357209
0.8032063271432058	0.8756480750112063	0.1837581734094454
0.9310176138470765	0.7465375064514246	0.2858447318477222
0.1778480553642381	0.1244855669377755	0.2612130365046409
0.9259035701089195	0.1199786383841540	0.1303554401067995
0.0521969936060853	-0.0008308801217461	0.2142307295353904

0.0538678426770446	-0.0011718624108464	0.3462932266791611
0.4278571885213250	0.1243597846359452	0.2629529512574631
0.1776898045621937	0.1185539514895297	0.1275971070849760
0.3036926348289000	-0.0013810717213258	0.2141349089246398
0.3019453073176167	-0.0009247837861370	0.3462975875751756
0.1780587228612321	0.3743156869915900	0.2507605020118194
0.9278692302949119	0.3647232367019001	0.1183396768439839
0.0536600286362665	0.2473190653688331	0.2074127529596182
0.0540882713725664	0.2578871303079656	0.3404524972099042
0.4268114426884281	0.3734872783042480	0.2497772568990714
0.1777830793484010	0.3671909634078015	0.1184558616552814
0.3020169951854595	0.2472793984786846	0.2069596084664424
0.3016281110401419	0.2563816455189172	0.3398050035993658
0.6785305552064251	0.1272050984923301	0.2684349712517474
0.4295041469583242	0.1198039275383001	0.1294774955865806
0.5548812260955106	-0.0009487212791419	0.2171067396811001
0.5515817792772074	-0.0004805693966140	0.3486706091645972
0.9272282066205352	0.1252972569302868	0.2643854331888632
0.6781701339865300	0.1203648083052319	0.1320623257432358
0.8007910077169850	-0.0009576271460504	0.2173103570959273
0.8040989601409885	-0.0011439364364903	0.3487607966069554
0.6768183424630316	0.3723201742921696	0.2484350218078111
0.4271465420857248	0.3657124542520348	0.1184368197872227
0.5529543621194841	0.2475541837396959	0.2105812672941264
0.5444625377480384	0.2628554596230462	0.3461200067135115
0.9295906165119642	0.3734112625248446	0.2492290192296615
0.6775650440526452	0.3641034445993301	0.1182320178428187
0.8023456356670320	0.2479785427602316	0.2121026517337712
0.8112383834360004	0.2673852463844945	0.3484401950605101
0.1777503900318033	0.6235175827473429	0.2511357701248639
0.9280404636012585	0.6328189853161147	0.1183876760829532
0.0534055371720164	0.4988705632763850	0.1977337912135917
0.0531523593562682	0.4984885424899856	0.3326542858381830
0.4271069739459920	0.6239822461558876	0.2505423175428421
0.1781177927884891	0.6307628095593090	0.1187985761017230
0.3021050657532884	0.4989938022895317	0.1984883506323940
0.3022200773270401	0.4986247815185987	0.3331979065531895
0.1780257945038365	0.8732475136904555	0.2614306588114738
0.9261000136836877	0.8787312342805722	0.1296234957137698
0.0534907948679260	0.7506416687106506	0.2071089015079988
0.0540563936249531	0.7407944634746298	0.3400492442428587
0.4288362789388073	0.8729076549201845	0.2643747186482431
0.1781089804206848	0.8789640607408707	0.1278877149343440
0.3026058623071039	0.7502585307101857	0.2082282166371238

0.3022750814783820	0.7408421683004462	0.3408530356190833
0.6781220826510541	0.6253657598256811	0.2486503552861867
0.4273392027464395	0.6327953617903047	0.1190931929350678
0.5510860995605700	0.4988688251288800	0.1955184067738992
0.5452076234117414	0.4978922402648434	0.3302675339816710
0.9288894890409228	0.6243130514881905	0.2495040727771421
0.6773395035944861	0.6335871579197648	0.1183493026006091
0.8035265149332840	0.4987402392046988	0.1943175439341260
0.8098895308089999	0.4968131791598006	0.3268664602213956
0.6775026499744140	0.8711553209630569	0.2682171385560681
0.4296804201298927	0.8775833855631980	0.1304224247824300
0.5536035204416693	0.7499360981049674	0.2121291917630389
0.5476109345337744	0.7294394838597168	0.3485279152298008
0.9282459122875970	0.8735864920229947	0.2629642756846017
0.6776452597030288	0.8775463763881828	0.1321354953000666
0.8025249933831240	0.7502869591925676	0.2108830492691009
0.8105535104318538	0.7361586576406431	0.3461362025312920
0.5270532500087128	0.3689471643925177	0.7163582597324868
0.6656178517611072	0.3393112360642229	0.5205291825290956
0.5862100129580378	0.3508535268068227	0.4184523534441643
0.7896214732674832	0.3555621949409383	0.4302386824935197
0.6125537331310232	0.4985293885985071	0.5101293285360859
0.6876770842624128	0.6396972666687833	0.5268196305162992
0.8112046718378449	0.5016840324428261	0.4832415326895705
0.5864503099812349	0.6347842201897691	0.4228051708328082
0.6831896626090803	0.4944587520899518	0.3901979183093006
0.7844801626849364	0.6445894122567167	0.4247242590975941
0.4438851585383461	0.3222837058869635	0.6151971941642604
0.5531792611071822	0.3357409141879483	0.6506420333340112
0.6306350414272779	0.3997407922014625	0.6137938707659256
0.3906425495485715	0.2781318809119517	0.6446324089760206
0.4565282356679035	0.2922480686476908	0.5654758007676786
0.4018530361679776	0.3865352681479535	0.6083283242600266
0.5950212413420213	0.2703738998667235	0.6556445859877914
0.5941101860204977	0.3710194981111842	0.7429416642880902
0.5959516193379120	0.4688186797638617	0.6182675929960776
0.7125258112788480	0.4029286211043165	0.6376228625548116
0.6504792080593510	0.7291944129930581	0.5686281917296335

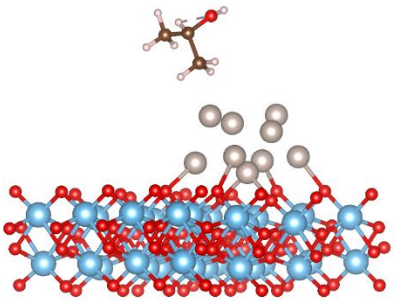

Supplementary Fig. 41e

Ti	O	Ru	C	H
32	65	9	3	8

0.0469256573408364	0.1270531693887844	0.1745753184067792
0.1710871395013795	0.0045005865785096	0.2869164732705397
0.2968208451548213	0.1269240220038584	0.1741433860605860
0.4216436013653274	0.0045976734616476	0.2886098548274887
0.0464242030266982	0.3761055599464954	0.1648456934835328
0.1710997959178832	0.2569788267983397	0.2806129753496895
0.2958039699429361	0.3766037503901492	0.1654069979229801
0.4172749074093957	0.2566255031993460	0.2796228807981869
0.5469805132720096	0.1284488288355032	0.1782169199319746
0.6710905094495183	0.0055044361347445	0.2905177389882900
0.7962236864818262	0.1286077197549117	0.1788346075263961
0.9207714961321783	0.0045818893800156	0.2890271827961519
0.5458754198973896	0.3755269577456641	0.1649662877692586
0.6711471994161272	0.2630585685849277	0.2870940279644264
0.7963962183494724	0.3755608469460594	0.1649180953070115
0.9242417945259893	0.2574536099846418	0.2807960243025605
0.0464722252624407	0.6331437392528715	0.1648065998660526
0.1729900125161962	0.5045150051520394	0.2744346143167040
0.2958600472094942	0.6325086557675025	0.1655574039957463
0.4181515033626281	0.5044784078680100	0.2705992196904912
0.0469380104513282	0.8817502566530697	0.1746576497916996
0.1711405721529643	0.7519036657491069	0.2809724294098871
0.2965051010211059	0.8818006906181377	0.1743548289385158
0.4182597488366932	0.7522315563513559	0.2799559657573448
0.5460494472911281	0.6336741764995395	0.1648307215779495
0.6716317243313745	0.5050080896053314	0.2638088223458592
0.7965147071460402	0.6336618519429543	0.1645696846753614
0.9290331329182562	0.5048353099790009	0.2684944273936752
0.5467633553322179	0.8814225331125877	0.1780380253992342
0.6711926929248543	0.7480396832862009	0.2853771853778835
0.7963808980072757	0.8812612528541920	0.1785296865307452
0.9233517215802955	0.7515777581070237	0.2811392437879818
0.1711027025212196	0.1300654495744397	0.2552713373171433
0.9192011972331779	0.1260148901338159	0.1245114832627402

0.0455595025517368	0.0044012802728308	0.2082794913102747
0.0473130054347426	0.0046398961344536	0.3405387803128452
0.4202978844964673	0.1295040761533267	0.2565811884135021
0.1714157794019917	0.1237435481691884	0.1217328866288581
0.2965429867315001	0.0043741364649116	0.2076763394817950
0.2951849237236034	0.0046492343111186	0.3401522871786208
0.1716368401272718	0.3800045081283678	0.2444206515541850
0.9213031312097082	0.3697345933599701	0.1123525064587722
0.0473071617828107	0.2529100273739293	0.2011374932275332
0.0478289219146743	0.2638231354450448	0.3344025090383319
0.4211638729857972	0.3792777116363494	0.2441818058399410
0.1715888058739197	0.3724387974494771	0.1123806375915954
0.2958111982991137	0.2529491925196171	0.2011812254015689
0.2941811733072079	0.2624609248680521	0.3341637191936226
0.6716876412145051	0.1325720794169089	0.2621051674334809
0.4240215478048892	0.1258378249601670	0.1236829087155396
0.5487434737384670	0.0048156667474704	0.2111031989578666
0.5447726373438375	0.0050752495088173	0.3427418286231652
0.9217563295783398	0.1303802940394634	0.2580418285402848
0.6719223365196196	0.1266974768681530	0.1260789037129449
0.7947528853645747	0.0048293659434476	0.2113984678041037
0.7974770896276102	0.0051402450087952	0.3429121658755104
0.6703442119858305	0.3785889912733995	0.2428070466934150
0.4209372417680504	0.3704745449347157	0.1127395768276422
0.5467631019254132	0.2536043717812114	0.2057246144907005
0.5369903961539952	0.2611464131473403	0.3416263239990844
0.9231175550702529	0.3793736214242319	0.2433502435052562
0.6711977206008527	0.3691400921731961	0.1125515655114640
0.7960784643578609	0.2539057318522907	0.2064590596557307
0.8061389396049717	0.2692936907780596	0.3416386580494032
0.1716437020798272	0.6290720055863758	0.2445871168798548
0.9215444755239864	0.6396048309768936	0.1121714709258560
0.0468987884391793	0.5046215299485554	0.1911112499379669
0.0475835144845130	0.5046515662034644	0.3264511571913701
0.4216865128997704	0.6297859369045267	0.2443485794237968
0.1718260408940611	0.6362067235851427	0.1124886655642513
0.2963241921684173	0.5045627650411973	0.1924164410920843
0.2965014224417566	0.5045790590243221	0.3270496945125925
0.1712631374248340	0.8788062257076132	0.2554292635022263
0.9194723217303814	0.8835742798931425	0.1243724940492350
0.0473007041843062	0.7561793706081547	0.2013711382924499
0.0473509776144271	0.7457001610844096	0.3345128548404007
0.4209234039561106	0.8793963534323718	0.2569740542548707
0.1714411350219792	0.8844720554943909	0.1217717358453206

0.2960056072934384	0.7559898902834297	0.2014689459018650
0.2947213140894262	0.7461660558803259	0.3342212840636710
0.6708042745399145	0.6311435432729642	0.2424678307760813
0.4209016674019224	0.6387521160964235	0.1128479194212191
0.5449715250230721	0.5046500385259223	0.1892075678427504
0.5407082671539761	0.5037711998044387	0.3241193440937795
0.9231924125565030	0.6301559544680715	0.2429666184498322
0.6711714216240281	0.6400968612033708	0.1122899226548175
0.7972086178458369	0.5046944811771035	0.1877362977612250
0.8047941537814349	0.5054710960653163	0.3203671881386108
0.6715645868892259	0.8771558898460042	0.2619042729787851
0.4236762891220387	0.8836714471649608	0.1238024746905735
0.5469584379675093	0.7559637649319795	0.2054164553841072
0.5382836340612972	0.7446663613050686	0.3417572891471179
0.9212356931600637	0.8788174324384007	0.2580597864548697
0.6718123755083987	0.8833539289547855	0.1259872204726459
0.7958390992414077	0.7557561622761978	0.2059553697365528
0.8040400011525027	0.7390853552928148	0.3416157864992810
0.6484336321542807	0.3703990298049165	0.7460338753338662
0.6630284054778119	0.3537566188568240	0.5148901899306334
0.5831095809218679	0.3540131432123148	0.4067842196056967
0.7834047993000773	0.3626870487372026	0.4218873955771881
0.6025527118258379	0.4993495723209869	0.4980889022850942
0.6591460668163218	0.6456694334384540	0.5168712313635943
0.8050698253131447	0.5053414189319947	0.4790936245463760
0.5756857240543864	0.6513357036370222	0.4115328045002977
0.6790457777274000	0.5030079159912697	0.3845424832168734
0.7765409999861970	0.6455993521053798	0.4210387223856307
0.4914771686472987	0.2968676914359349	0.7022054784864427
0.6160926803336113	0.2952048979363037	0.7065170031820021
0.6706479171078714	0.2993168395959844	0.6371011241161643
0.4559275590664105	0.2940193763764899	0.7525680342405492
0.4608055895418164	0.2399074005403796	0.6731867804508873
0.4636467466161047	0.3587275544640995	0.6780824258785978
0.6428700818980796	0.2327317771784053	0.7309934408334502
0.7249179779839828	0.3634631319226193	0.7593373941501667
0.6485353690766279	0.3675755056011438	0.6174418739480502
0.6396259418757546	0.2444928128459696	0.6052035824879513
0.7606533192174948	0.2946877088031168	0.6401849825325161
